# Annulative π-extension of BODIPYs made easy *via* gold(i)-catalyzed cycloisomerization†

**DOI:** 10.1039/d0sc01054e

**Published:** 2020-06-12

**Authors:** Jorge Labella, Gonzalo Durán-Sampedro, M. Victoria Martínez-Díaz, Tomás Torres

**Affiliations:** Departamento de Química Orgánica, Universidad Autónoma de Madrid 28049 Madrid Spain tomas.torres@uam.es victoria.martinez@uam.es; Institute for Advanced Research in Chemical Sciences (IAdChem), Universidad Autónoma de Madrid 28049 Madrid Spain; IMDEA-Nanociencia, Campus de Cantoblanco 28049 Madrid Spain

## Abstract

Here we report gold(i)-catalyzed cycloisomerization as a new powerful synthetic tool for the preparation of π-extended BODIPY derivatives. The catalytic system PPh^F^_3_AuCl/AgSbF_6_ enables the synthesis of [*b*]-[2,1]naphtho-fused-BODIPYs (**2a–2c**) under mild conditions, in excellent yields and short reaction times. The reaction is totally regioselective to the 6-*endo*-dig product and for the α-position of the BODIPY, which is both the kinetically and thermodynamically favored pathway, as supported by the free energy profile calculated by means of Density Functional Theory (DFT). Moreover, this methodology also allows the synthesis of two new families of [*b*]-aryl-fused-BODIPYs, namely, [3,4]phenanthro- (**2e** and **2f**) and [1,2]naphtho-fused (**2g**) BODIPYs. Their molecular and electronic structures were established by NMR and UV-vis spectroscopies as well as single-crystal X-ray diffraction analysis. As can be noted from the X-ray structures, **2a**, **2e** and **2g** present interesting structural differences at both the molecular and packing level. Interestingly, despite being isomers, the UV/vis spectra of **2a** and **2g** revealed significant differences in their electronic structures. The origin of this finding was studied by Time-Dependent DFT calculations. Calculated DFT Nuclear Independent Chemical Shift (NICS(0)) values also supported the different electronic structures of **2a** and **2g**.

## Abstract 2

Here we report gold(i)-catalyzed cycloisomerization as a new powerful synthetic tool for the preparation of π-extended BODIPY derivatives.

## Graphical abstract

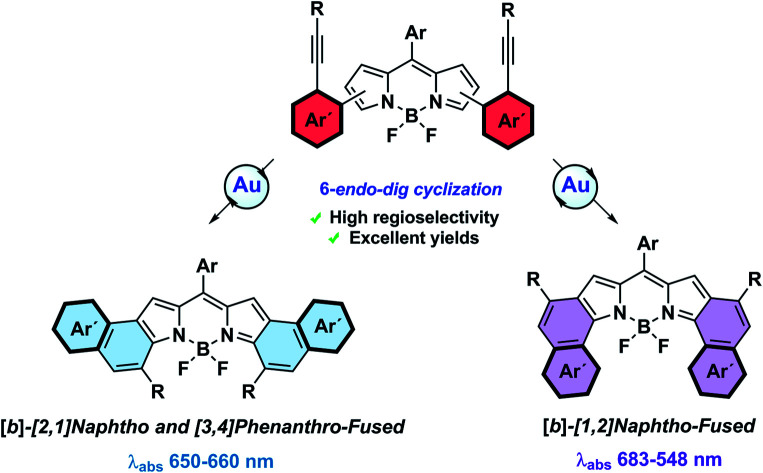

## Introduction

Gold(i)-catalyzed activation of alkynes has emerged over the last decade as a powerful tool for the construction of molecular complexity from readily accessible starting materials.^1^ Due to relativistic effects, gold(i) exhibits a high affinity for the alkyne π-bonds and forms η^2^-alkyne complexes, which are susceptible to react with a variety of heteroatom- or carbon-based nucleophiles under mild reaction conditions.^2^ More recently, this kind of catalysis has gained increased attention in the field of molecular materials, allowing for the efficient synthesis of novel highly conjugated systems, such as enantiopure helicenes,^3^ polycyclic aromatic hydrocarbons (PAHs)^4^ or other large heteroaromatic derivatives,^5^ with absorption bands shifted to red.

**Scheme 1:**
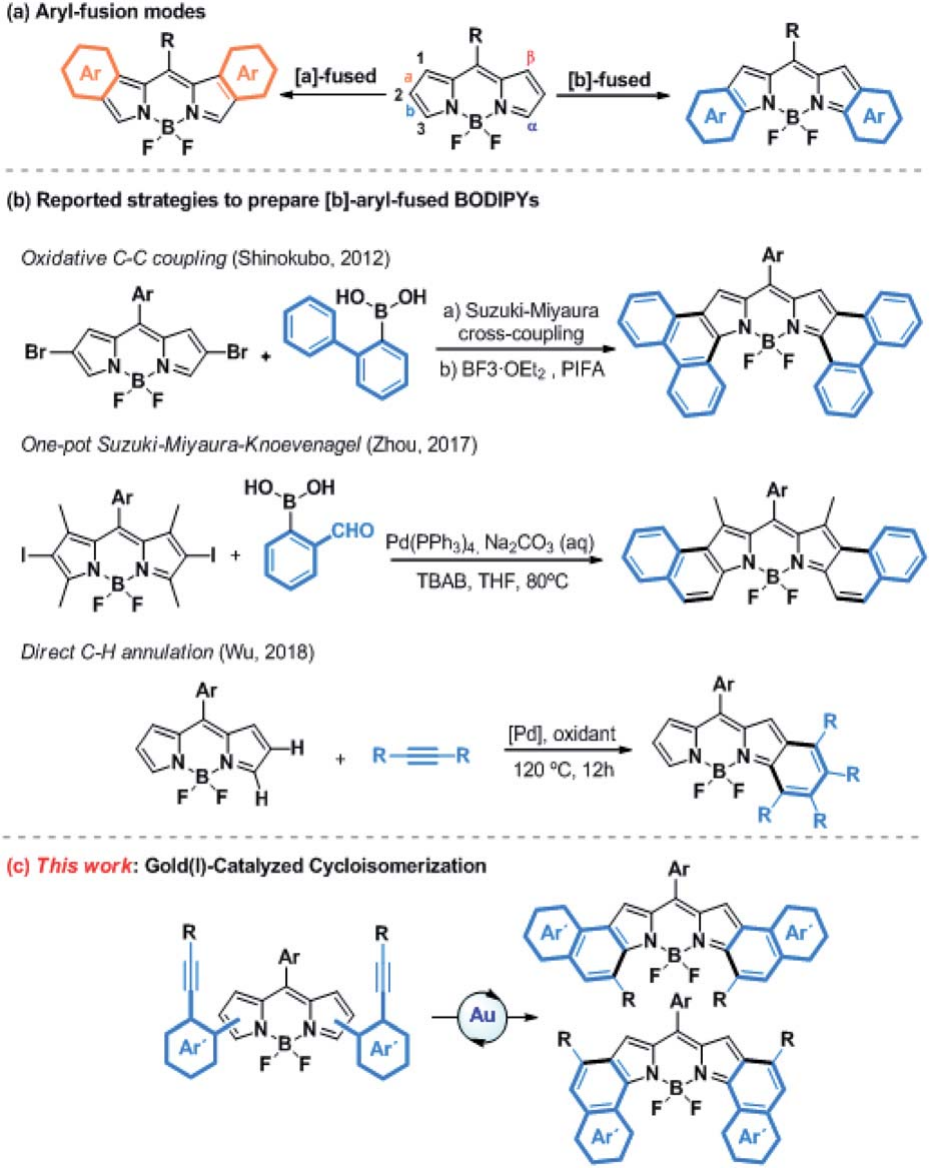
(a) Chemical structures of [*a*]- and [*b*]-aryl-fused BODIPYs. (b) Reported post-functionalization reactions of the BODIPY core to prepare [*b*]-aryl-fused BODIPYs. (c) Strategy described in this work.

Porphyrinoids are well-known chromophores that are nowadays widely used in a plethora of applied fields.^6^ Boron-dipyrromethenes (BODIPYs) and their derivatives, which are related to porphyrinoids and present unique redox and photophysical properties (*e.g.*, excellent photostability, intense absorption profiles, high quantum yields, and small Stokes shifts), hold a privileged position.^7^ These properties have skillfully been used in a wide range of applications, such as photovoltaic cells,^8^ photodynamic therapy (PDT),^9^ OLEDs^10^ or probes for bioimaging,^11^ just to name a few. In this context, absorption and emission in the Near-Infrared Region (NIR) is highly desirable. Consequently, much effort has been devoted to developing synthetic methodologies to extend the π-system of BODIPYs. Among them, the fusion at the [*a*]- or [*b*]-bond (Scheme 1a) with aromatic rings such as benzene, naphthalene or phenanthrene is particularly promising.^12^ Despite the ring fusion at the [*b*]-position results in a higher bathochromic shift and more stability,^13^ the [*b*]-aryl-fused-BODIPYs have so far been less explored compared to the [*a*]-ones due to their more challenging synthesis. However, over the last few years, the post-functionalization of the BODIPY core by annulation has gained increased attention as a new efficient synthetic strategy (Scheme 1b). In this regard, several chemical transformations such as oxidative aromatic coupling,^14^ one-pot Suzuki–Miyaura–Knoevenagel reaction^15^ or direct C–H palladium catalyzed annulation,^16^ have been recently reported to prepare [*b*]-phenanthro and [*b*]-naphtho-fused-BODIPYs. However, these methodologies, although elegant, are quite limited in terms of scope, yield, group tolerance and availability of the starting material. Therefore, the efficient and flexible synthesis of [*b*]-aryl-fused-BODIPYs remains a synthetic challenge.

Considering the aforementioned features of gold(i)-activation of alkynes, we envisaged the synthesis of [*b*]-aryl-fused-BODIPYs using such catalysis as a key step. To do that, the well-known metal-catalyzed 6-*endo*-dig cyclization of *ortho*-alkynyl-biaryl compounds was selected as the model reaction (Scheme 1c).^17^ Besides the preparation of [*b*]-aryl-fused-BODIPYs, this study would provide a new tool for BODIPY functionalization, where the pyrrole rings act as a nucleophile toward gold-activated alkynes, giving rise to further investigations of other gold-catalyzed reactions.

Herein, we report the efficient, versatile and mild synthesis of [*b*]-naphtho and [*b*]-phenanthro-fused-BODIPYs using gold(i) catalyzed cyclization as a key step. This method, where several factors such as catalytic conditions and scope have been studied, showed excellent regioselectivity for the 6-*endo*-dig cyclization product and for the α-position of the BODIPY, whose origin has been investigated by Density Functional Theory (DFT) calculations. Moreover, two new families of BODIPYs, [3,4]-phenanthro- and [1,2]-naphtho-fused BODIPYs, have been synthesized using this methodology and characterized by nuclear magnetic resonance (NMR) spectroscopy, mass spectrometry, and UV-vis spectroscopy as well as single crystal X-ray diffraction analysis. Finally, the structural and electronic differences between these [*b*]-aryl-fused BODIPYs are discussed, considering the experimental results and supported by DFT and time-dependent (TD)-DFT calculations.

## Results and discussion

### Optimization and scope of gold-catalyzed cycloisomerization

The 2,6-bis(2-(propynyl)phenyl)-BODIPY **1a**, which was prepared from the 2,6-dibromo derivative by Suzuki cross-coupling (see the ESI†), was chosen as the model substrate to evaluate the gold(i)-catalyzed cycloisomerization for forming the [*b*]-naphtho-fused BODIPY **2a** (Table 1). This study was initiated by testing different transition metals, such as In(iii), Pt(ii) and Au(iii), that have been previously used in this kind of cycloisomerization (Entries 1–3), however, the reaction led to a complex mixture of colored products. Therefore a milder catalyst, such as the well-known PPh_3_AuCl **A** was used. **A** initially failed in showing catalytic activity at reflux in toluene or DCE. Gratifyingly, the *in situ* formation of a cationic gold(i)-complex using 10% of AgSbF_6_ as a chloride scavenger dramatically increased the activity of **A**. In fact, under these conditions, after 25 min at 25 °C, **2a** was obtained as a major product in 85% yield (Entry 6). Interestingly, when the perfluorinated catalyst **B** was employed instead of **A**, the reaction was quantitatively completed after 15 min (Entry 7). This result can be easily explained considering previous studies by Alcarazo *et al.* on the influence of ligand π-acidity in the metal-catalyzed cycloisomerization of *ortho*-alkynylated biaryls, where they revealed that electron-poor ancillary ligands substantially enhance the π-acidity of metals and hence their ability to activate alkynes toward nucleophilic attack, which is the rate-determining step.^18^ Thus, the commercially available PPh^F^_3_AuCl catalyst **B** was selected for further optimization. Toluene as solvent led to longer reaction times and lower yield, probably due to the poorer stabilization of the charged intermediates formed during the reaction (Entry 8).^17^ Next, the effect of the counterion was also investigated. Chloride scavengers AgOTf (Entry 9) and AgSbF_6_ gave similar results in terms of yield, albeit the reaction time was slightly shorter in the latter case, as expected given the lower basic character of SbF_6_^−^ anions.^19^ Lowering the catalyst loading to 5 mol% (Entry 10) resulted in incomplete conversion of **1a**. As expected, the control experiment (Entry 11) confirms that the cationic gold(i) complex is the catalytically active system.

**Table 1:** Optimization of the metal-catalyzed cycloisomerization of **1a**^a^

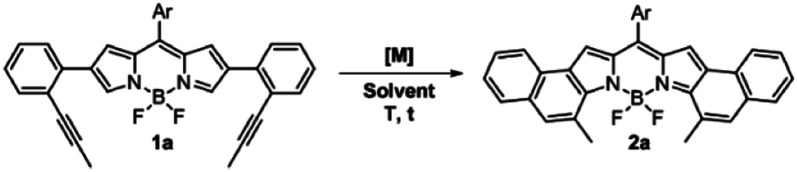						
Entry	[M] (10%)	AgX (10%)	Solvent	*T* (°C)	*t* (min)	Yield (%)
1	PtCl_2_	—	DCE	rt → 85	480	—b
2	InCl_3_	—	DCE	rt → 85	1440	—b
3	AuCl_3_	—	DCE	rt	1440	—b
4	PPh_3_AuCl (A)	—	DCE	rt → 85	1440	0
5	PPh_3_AuCl (A)	—	Toluene	rt → 115	1440	0
6	PPh_3_AuCl (A)	AgSbF_6_	DCE	rt	25	85
**7**	**PPh** ^**F**^_**3**_ **AuCl (B)**	**AgSbF**_**6**_	**DCE**	**rt**	**15**	**>95**
8	PPh^F^_3_AuCl (B)	AgSbF_6_	Toluene	rt	480	52
9	PPh^F^_3_AuCl (B)	AgOTf	DCE	rt	25	92
10	PPh^F^_3_AuCl (B)^c^	AgSbF_6_^c^	DCE	rt	1440	74
11	—	AgSbF_6_	DCE	rt	1440	0

a Yields were determined after purification using a chromatography column.

b Complex mixture of colored products.

c 5% of catalyst loading. Ar = mesityl-.

**Scheme 2:**
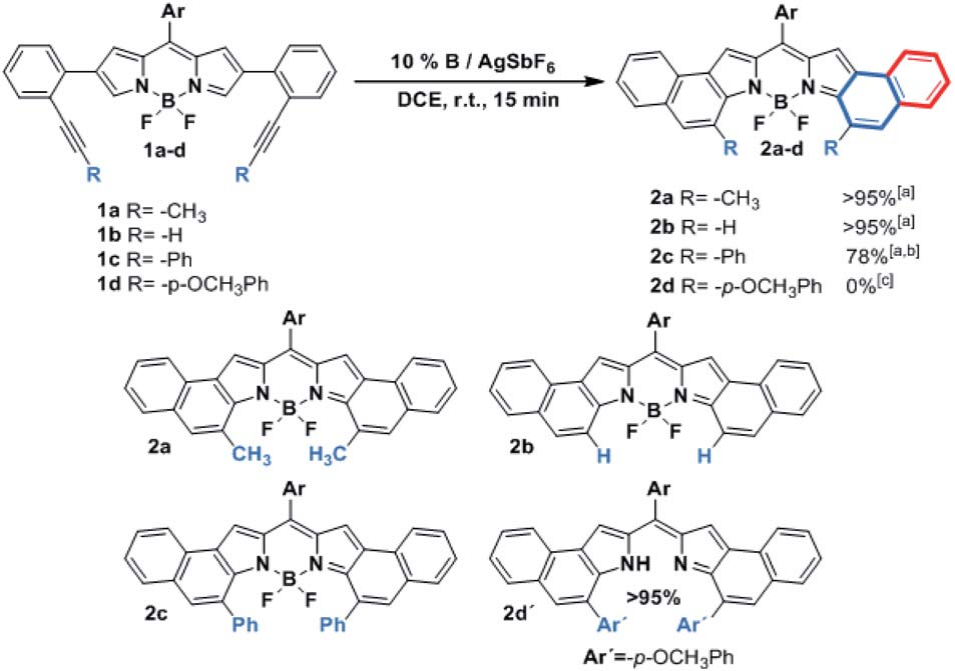
Scope of the cycloisomerization of **1** to form **2** with different substituted alkynes. [a] Yields were determined after purification using a chromatography column. [b] 20% PPh_3_^F^AuCl/AgSbF_6_, 40 °C, DCE and 1 h. [c] not detected, and **2d′** was quantitatively obtained.

As can be seen from the structure of **2a**, the methyl groups at the central benzene ring (Scheme 2, blue benzene) are close and point to the BF_2_ moiety, so the cycloisomerization could be hindered with bulkier substituents. Moreover, taking the previous work on [*b*]-naphtho-fused-BODIPYs into account,^15^ it was expected that the central benzene ring might have a relevant impact on the electronic properties owing to its involvement in both the HOMO and the LUMO of the molecule, unlike the outer benzene ring (Scheme 2, red benzene) which is only involved in the HOMO. For these reasons, we considered it relevant to evaluate the scope of the cycloisomerization with different substituents on the alkyne (Scheme 2).

Under the optimized conditions, cycloisomerization was first proved with –H as the substituent. As the model reaction, the cyclization product **2b** was quantitatively isolated. Subsequently, the reaction with a bulkier group such as phenyl was carried out. Remarkably, in this case 20% of catalyst loading, 1 h and 40 °C were needed for the total disappearance of the starting material, affording **2c** in 78% yield. This finding points out to a notable steric hindrance during the cyclization. In the interesting case of *para*-methoxyphenyl as the substituent, the expected BODIPY **2d** was not detected. Instead, the free dipyrromethene **2d′** was the isolated single product. Such results can be rationalized by analysing the X-ray structure of **2d′** (see the ESI†). The steric hindrance between the methoxy moieties results in a slight deviation in the planarity of the dipyrromethene core that enables deborylation (see Fig. S6.1†).

It is important to highlight the pronounced regioselectivity of the reaction. First, only the 6-*endo-dig* cyclization is detected, over the also conceivable 5-*exo-dig* mode. These results are in total agreement with other reported cationic gold(i)-catalyzed cycloisomerization reactions,^3,5^ where total 6-*endo*-dig selectivity is also observed. In addition, the reaction is completely regioselective to the α-position of the BODIPY over the β-one.

### DFT calculations of the reaction mechanism: α-regioselectivity

**Fig. 1:**
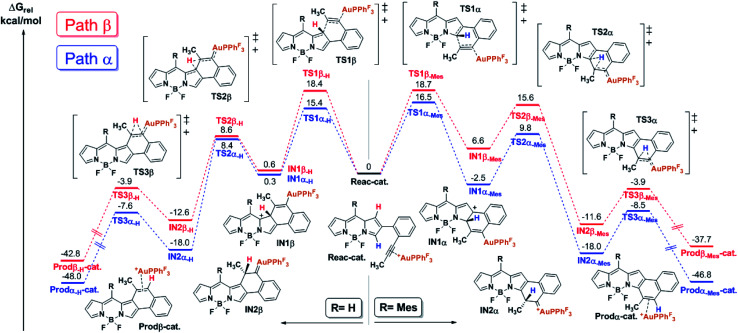
Free energy profiles (kcal mol^−1^) for the cycloisomerization of 2-(2-(propynyl)phenyl)-BODIPY (model substrate for this study). The red and blue lines represent the calculated profiles for the β- and α-paths, respectively. Such calculated pathways were elucidated with H- (left side) and Mes- (right side) as *meso* substituents.

In order to shed light on the origin of this remarkable regioselectivity, the reaction pathway of the cycloisomerization at both the α- and β-positions was quantitatively explored by means of DFT calculations (see the ESI† for computational details). Fig. 1 depicts the calculated free energy profile of such pathways for the selected model BODIPY (*i.e.*, 2-(2-(propynyl)phenyl)-BODIPY). Furthermore, in order to evaluate the effect of the *meso* substituent, the free energy profile was computed with both H- and Mes- at the *meso* position (left and right side of Fig. 1, respectively).

The first step of the reaction involves the intramolecular attack of the pyrrole ring by the activated alkyne to form the intermediates **IN1α-H/Mes** and **IN1β-H/Mes** through the transition states **TS1α-H/Mes** and **TS1β-H/Mes**, respectively. Importantly, **TS1α-Mes** lies 2.2 kcal mol^−1^ lower than **TS1β-Mes** and **TS1α-H** lies 3.0 kcal mol^−1^ lower than **TS1β-H,** suggesting that, regardless of the *meso* substituent, the nucleophilic attack at the α-position is more favorable. Considering that such an attack is governed by orbital interactions (soft acid and base),^2^ this result can be rationalized by inspection of the HOMO of the complex **Reac-cat** (Fig. S6.5†) where, although the α-carbon contributes considerably, the β-carbon is barely involved. Our calculations indicate that this step exhibits the highest energy barrier for both paths and therefore it is expected to have a strong influence on the kinetics of the whole process. Interestingly, if **IN1α-Mes** is 9.1 kcal mol^−1^ more stable than **IN1β-Mes** then the α-attack is exergonic (−2.5 kcal mol^−1^) while the β-attack is endergonic (6.6 kcal mol^−1^). However, in the case of **IN1α/β-H**, both intermediates have similar energy and both processes are slightly endergonic. The next step along the reaction coordinates involves a 1,2-H shift through **TS2α/β-H/Mes** that does not depend much on the path considered and leads to intermediates **IN2α/β-H** and **IN2α/β-Mes** which are respectively 20–26 kcal mol^−1^ and 15–18 kcal mol^−1^ more stable than their precursors, and the energies of the following transition states **TS3α/β-H/Mes** are much lower than those of **TS2α/β-H/Mes**. Thus, the formation of Au-carbenes **IN2α/β-H/Mes** is likely to be irreversible. Finally, a second very exergonic 1,2-H shift that requires moderate activation (barriers of 8–11 kcal mol^−1^) and is not affected by the path or substituent considered, leads to naphtho-fused-BODIPY-Au π-complexes **Prodα/β-H/Mes-cat**, which after dissociation regenerate the catalyst and deliver the products.

With these result in hand, it can be concluded that, due to the electronic differences between the α- and β-carbons, the nucleophilic attack (rate-determining step) is kinetically favored in the case of the α-cyclization and does not arise from steric interactions with the *meso* substituent. In the case of Mes-at the *meso* position, the β-attack is thermodynamically unfavored and such an equilibrium should be shifted to the starting materials which, in turn, evolve into the thermodynamically favored **IN1α-Mes**. In summary, the α-fused BODIPY is both the kinetic and thermodynamic product, resulting in a theoretical regioselectivity which is in good agreement with the experimentally observed selectivity.

### Synthesis of [3,4]phenanthro- and [1,2]naphtho-fused BODIPYs

**Scheme 3:**
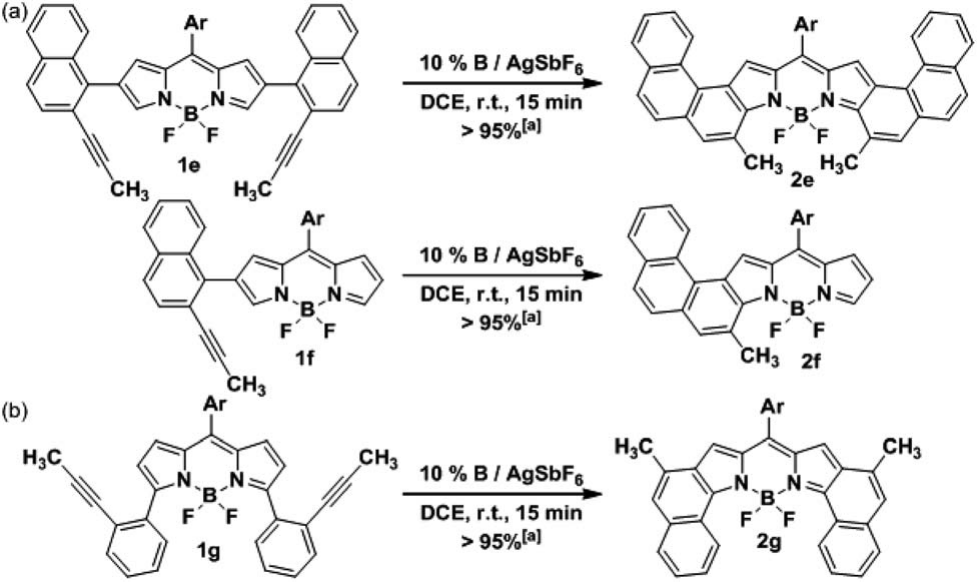
Synthesis of (a) [3,4]phenanthro-fused-BODIPYs **2e** and **2f**, and (b) [1,2]naphtho-fused-BODIPY **2g**. [a] Yields were determined after purification using a chromatography column.

In order to assess the versatility of our methodology, the synthesis of other fused-BODIPY families was targeted. Firstly, we expanded further the conjugation of **2a** by attaching another benzene ring at the [*h*]-bond of the naphthalene fragment. To this end, the precursor **1e** was assembled similarly by Suzuki cross-coupling (see ESI†) and then, was subjected to cycloisomerization under the previously optimized conditions (Scheme 3a). The [3,4]phenanthro-fused BODIPY **2e** was isolated also in a quantitative yield, being the first example reported of this highly conjugated family. Moreover, **2e** comprises the highest number of consecutively fused benzene rings found in a BODIPY derivative. Interestingly, despite the higher steric hindrance expected during the cyclization compared with **2a**, the activity of our catalytic system did not decrease. Additionally, the mono-fused counterpart of **2e** and **2f** was successfully prepared from **1f** following a similar synthetic route (Scheme 3a).

Next, we envisioned that the gold(i)-catalyzed cyclization of **1g** would afford the [1,2]naphtho-fused-BODIPY **2g** (Scheme 3b), which is the isomer of **2a**. Surprisingly, this family of BODIPY has remained unreported to date. Initial efforts by the Burgess group yielded only the mono-[1,2]naphtho-fused BODIPY after oxidation of the hydrogenated counterpart with DDQ.^20^ Semiempirical calculations suggested that the second oxidation is hindered by steric interactions. More recently, a mono-[1,2]naphtho-fused BODIPY was serendipitously reported by Wu *et al.* in an attempt to prepare [2,1]naphtho BODIPYs *via* the Scholl reaction.^21^ Again, the [1,2]-fusion at both pyrrole rings was not achieved. The precursor **1g** was prepared also by Suzuki cross-coupling (see the ESI†) using the reported 3,5-dichloro-BODIPY as the starting material. Finally, the cycloisomerization of **1g** was carried out leading to **2g** in a quantitative yield. As expected, the reaction time is shorter than that in the case of **2a** (5 min *vs.* 15 min). This result can be rationalized considering that the 2,6-carbons are better nucleophiles than 3,5-carbons, which usually behave as electrophiles.^7^ It should be noted that the synthesis of three different [*b*]-aryl-fused BODIPYs was readily achieved by just changing the position or the design of the boronic acid.

### Characterization and theoretical studies of [1,2], and [2,1]naphtho- and [3,4]phenanthro-fused BODIPYs

#### NMR profiles

The NMR spectra of **2a–2f** present characteristic signals allowing their unequivocal identification. For example, in the case of ^1^H-NMR, the singlet corresponding to the α-protons disappears after the cyclization. Moreover, the signal corresponding to the closest proton to the BODIPY core appears quite deshielded as a result of the diatropic ring current of the pyrrole and benzene rings. Similarly, **2g** also shows these downfield signals. More interestingly, in the case of **2a**, **2e** and **2f**, the carbons of the methyl groups, which appear as a triplet in the ^13^C-NMR, exhibit an unusual through-space coupling with the fluorine atoms (*J*_C–F_ = 11 Hz, see Fig. S5.25, S5.36 and S5.39†).^22^

#### X-ray structures and packing

**Fig. 2:**
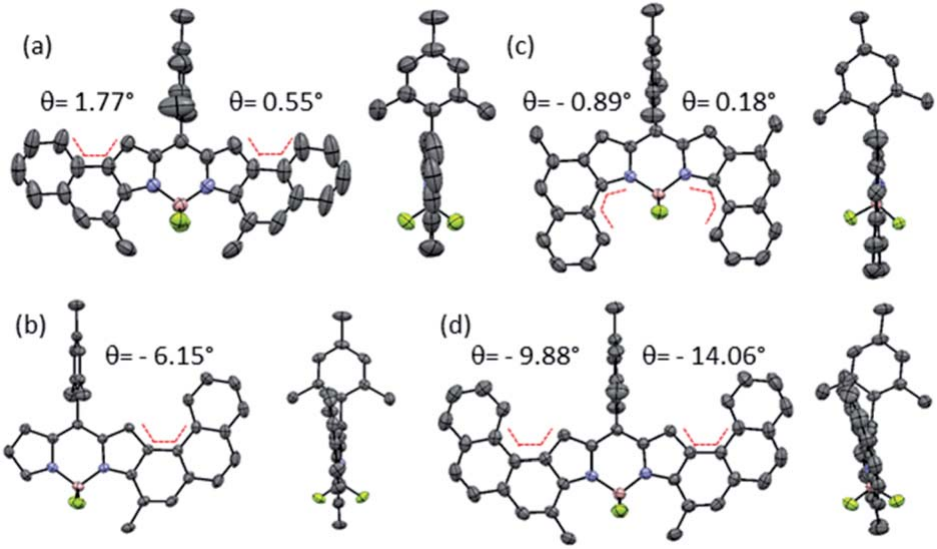
Front (right) and side (left) view X-ray structures of (a) **2a**, (b) **2f**, (c) **2g**, and (d) the most tensioned unit of **2e** in the unit cell. Atom's colour code: carbon in grey, nitrogen in blue, iodine in violet, boron in pink, fluorine in green and the angle *θ* in red.

Single crystals of **2a**, **2e**, **2f** and **2g** suitable for X-ray analysis were obtained by slow evaporation of a CHCl_3_/hexane mixture (Fig. 2). The non-planarity of **2a**, **2e**, **2f** and **2g**, defined using the torsion angle (*θ*) between the four atoms embedded in the bay-region, is shown in Fig. 2. In the case of **2a** and **2g**, the structure is nearly planar (*θ* = 1.77–0.55°). In contrast, the diffraction analysis of **2e** and **2f** revealed a slight deviation from planarity (*θ* = 6.15–14.06°) arising from the steric interaction of the phenanthrene skeleton. These structural features point out to an effective π-conjugation of these systems. As expected, the *meso*-mesityl group is in a nearly perpendicular orientation in all cases. Despite being isomers, **2a** and **2g** present significant and interesting differences in the crystal packing (see S3.3–6†). The crystal of **2a** shows highly unidirectional oriented packing governed by π–π interactions, which are reinforced by Csp^3^–H⋯H–Csp^2^ interactions, and where the molecules approach each other in a parallel manner. In the case of **2g**, the molecules are organized in dimers linked together through dipole–dipole B–F interactions. These dimers interact perpendicularly with the neighboring dimers by means C–H⋯π and C–H⋯F–B interactions and then, in contrast to its isomer, there is no directional preference during the packing. As its lower counterpart **2a**, **2e** also exhibits a unidirectional oriented packing where the π–π interactions also play an essential role. Interestingly, these π–π interactions are established between two phenanthrene moieties or between a phenanthrene fragment and a pyrrole fragment, depending on the unit of the unit cell considered. Additionally, other interactions such as C–H⋯F–B or Csp^3^–H⋯H–Csp^2^ reinforce this molecular arrangement. This orientational organization of **2a** and **2e** could be interesting for the application of these derivatives as organic semiconductors.^23^ Surprisingly, in the crystal structure of **2f** the molecules are arranged in a perpendicular manner each establishing mainly C–H⋯π interactions instead of π–π interactions suggesting that the aromatic ring fusion at both pyrrole rings is needed to knit a directional solid state π-network.

#### UV/vis absorption spectra and theoretical analysis of the electronic structure

**Fig. 3:**
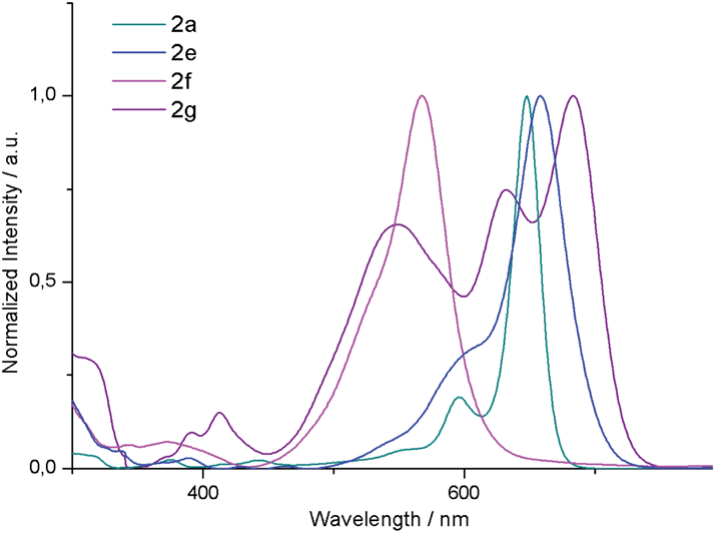
UV/Vis absorption spectra of **2a**, **2e**, **2f** and **2g** in dichloromethane.

The UV/Vis absorption spectra of **2a**, **2e**, **2f** and **2g** are shown in Fig. 3. The cyclization product **2a** exhibits an intense Q band at 650 nm, which is remarkably red-shifted compared to **1a**, indicating an effective π-conjugation effect induced by the fused naphthalene. Notably, this absorption presents a large molar extinction coefficient (more than 170 000 M^−1^ cm^−1^). **2b** and **2c** display similar absorption spectra also reaching the maximum at 650 nm. Interestingly, **2e** exhibits a Q band bathochromically shifted only by 10 nm relative to that of **2a**. From these results, it can be concluded that the absorption maxima of these derivatives are not significantly affected by substitution of the central or outer benzene rings. It is worth noting that the absorption profile of **2g** is substantially different from that of its isomer **2a**. In this case, the absorption spectrum presents three intense bands at 683, 632 and 548 nm which do not show a clear different trend as a function of the solvent polarity. Moreover, it should be noted that the molar absorption coefficient of these bands (50 000, 32 000 and 25 000 M^−1^ cm^−1^, respectively) decreases drastically compared to **2a**, which may be attributed to the decreased transition dipole moment, which in the case of BODIPYs, is polarized along the longer molecular axis.^24^ Considering the emission properties, the quantum yields of these systems fall below 0.1 as a result of the [*b*]-ring fusion.^15^

**Fig. 4:**
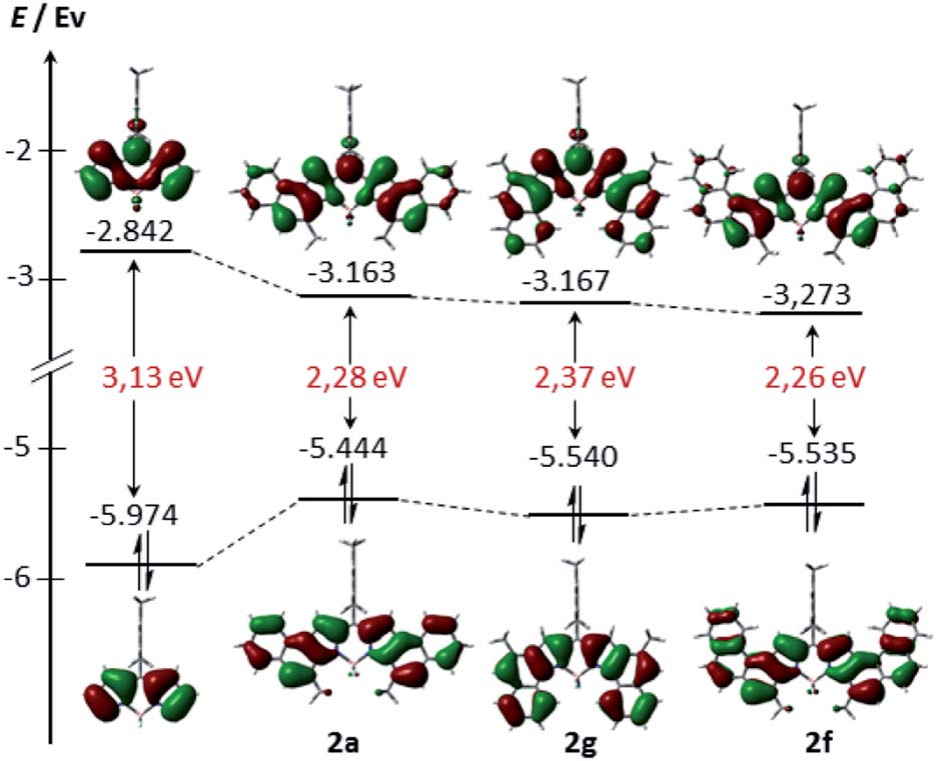
Energy diagrams and Kohn–Sham orbital representations of the HOMO and LUMO of unsubstituted BODIPY **2a**, **2f** and **2g**. All calculations were performed at the B3LYP/6-31G level.

In order to gain further insight into the electronic structure, density functional theory (DFT) calculations were conducted at the B3LYP/6-31G(d) level (see the ESI† for more details). As can be seen from Fig. 4, the HOMO and LUMO of **2a**, **2b** and **2f** are extended over the fused benzene rings. Consequently, these derivatives exhibit lower HOMO–LUMO band gaps compared to their unsubstituted counterpart arising from both the stabilization of the LUMO and the destabilization of the HOMO, as expected, given the α ring fusion.^13,15^ Remarkably, while the central benzene rings of **2a** are involved in both the HOMO and the LUMO delocalization, the outer benzene ring only significantly affects the HOMO. This fact would explain the slight bathochromic shift of the maximum absorption observed in **2e** relative to **2a**.

In addition, nucleus-independent chemical shift (NICS(0)) values at the B3LYP/6-31G(d,p)//B3LYP/6-31G(d) level were calculated to evaluate the ring current effect of these systems (Fig. S6.2†). For the benzene rings directly linked to pyrroles (Scheme 2, blue benzene), NICS(0) values are −3.0, −3.1 and −2.5 in **2a**, **2e** and **2g**, respectively. These values are substantially lower than those reached in the benzo[*e*]indole, naphtho[1,2-*e*]indole and benzo[*g*]indole (−8.5, −8.2 and −8.5, respectively), indicating a higher double-bond character of the K-region of **2a**, **2e** and **2g**. Interestingly, all the NICS(0) values of the aromatic rings that contain the BODIPY core of **2g**, are lower than those of **2a** (see Fig. SX†). In line with the spectroscopic results, this result suggests different π-conjugation in **2a** compared to its isomer **2g**.

Time-dependent (TD) calculations at the PCM(CH_2_Cl_2_)-TD-BMK/6-31G(d,p)//B3LYP/6-31G(d) level were carried out to assign the nature of the absorptions observed in the UV-vis spectra of **2a**, **2e**, and **2g**.^25^ Selected TD-DFT transition energies, oscillator strengths (*f*) and molecular orbital configurations are shown in Table 2. With regard to the calculated spectra of **2a** and **2e**, only one band corresponding to the HOMO–LUMO transition is observed. Moreover, the calculations predict a 10 nm bathochromic shift of **2e** relative to **2a**, which is in good agreement with the experimental result. In the case of **2g**, the spectrum displays three intense bands with oscillator strength values of 0.59, 0.46 and 0.32, which well reproduced the shape of the experimental spectra (see S6.3†). The lowest-energy absorption band is mainly derived also from the HOMO–LUMO transition, while the second one arises from the HOMO^−1^–LUMO transition. Interestingly, the highest-energy band arises from a charge-transfer transition between the *meso*-mesityl ring (HOMO^−5^) and the BODIPY core (LUMO) orbitals, which are minimally overlapped through the mesityl moiety (see S6.4†). This unprecedented finding renders the [1,2]naphtho-fusion a novel and powerful strategy to achieve a wide absorption in the visible range with BODIPY derivatives.

**Table 2:** Selected transition properties of **2a**, **2e** and **2g** calculated at the BMK/6-31G(d,p)//B3LYP/6-31G(d) level of theory

Compd.	Energy (nm)	*f* a	Orbitals^b^ (coefficient)
**2a**	556	1.46	H → L (70%)
**2e**	566	1.57	H → L (70%)
**2g**	551	0.59	H → L (68%), H-1 → L (17%)
	482	0.46	H → L (17%), H-1 → L (68%)
	335	0.32	H-5 → L (68%), H-7 → L (11%)

a Oscillator strength.

b MOs involved in the transitions (H and L denote the HOMO and LUMO).

## Conclusions

In this work, we have reported the efficient synthesis of β-naphtho- and phenanthro-fused-BODIPYs by means of gold(i)-catalyzed cycloisomerization as a key step, being the first example of functionalization of a BODIPY using this kind of catalysis. We found that the catalytic system PPh^F^_3_AuCl/AgSbF_6_ enabled the synthesis of **2a** in a quantitative yield at room temperature. The reaction is compatible with other substituents on the alkyne such as -H or –Ph; however, in the case of bulkier *p*-methoxyphenyl BF_2_ decomplexation takes place as a result of steric interactions. The reaction is totally regioselective toward the *6-endo-dig* and α-cyclization product. The origin of the α-selectivity was studied by DFT calculations and it can be concluded that it arises from (a) the higher nucleophilicity of the α-carbon, which lowers the energy of the nucleophilic attack (rate-determining step), (b) the higher stability of all intermediates and transition states involved in the α-path and (c) in the particular case of Mes- as a substituent, a shifted equilibrium to the starting materials in the β-attack. The synthesis of other β-aryl-fused-BODIPYs, [3,4]phenanthro- (**2e** and **2f**) and [1,2]naphtho-fused (**2e**), was also successfully achieved using this methodology just by changing the position or the design of the *ortho*-alkynyl-aryl moiety. Their molecular and electronic structures were compared by NMR, UV-vis and single-crystal X-ray diffraction analysis. With regard to the X-ray structures, **2a**, **2e** and **2g** present significant crystallographic differences. While **2a** and **2g** display a quasi-planar π-skeleton, **2e** shows a slightly curved π-skeleton owing to its [4]-helicene character. In addition, **2a** and **2e** exhibit a unidirectional packing based on π-stacking interactions, which is not favoured in the case of **2g**. The UV/vis spectra of **2a–2e** exhibit an intense peak (*ε* = 100 000–200 000 M^−1^ cm^−1^) at 650–660 nm. In contrast, **2g** displays three intense absorption bands in the range of 450 to 750 nm. Time-dependent DFT calculations revealed that these transitions arise from the π–π* transition from the HOMO and the HOMO^−1^ to LUMO and a charge transfer transition from the *meso*-mesityl group (HOMO^−5^) and LUMO. NICS(0) values also indicate a different π-conjugation in **2a** and **2g**, as revealed by the less aromatic character of each benzene ring of **2g**.

Given the reactivity of the pyrrole ring of the BODIPY towards Au-activated alkynes, this work serves as a proof of concept to explore other gold(i)-catalyzed reactions to functionalize BODIPY or other pyrrole-based porphyrinoids. In this sense, further transformations are being investigated in our laboratories.

## Conflicts of interest

The authors declare no competing financial interest.

## Supplementary Material

SC-011-D0SC01054E-s001

SC-011-D0SC01054E-s002
