# Supplementary material for: Annulative π-extension of BODIPYs made easy via gold(i)-catalyzed cycloisomerization
Source: Chem Sci. 2020 Jun 12;11(39):10778–85. doi: 10.1039/d0sc01054e (PMC8162369; doi:10.1039/d0sc01054e)
Supplement: SC-011-D0SC01054E-s001 [file SC-011-D0SC01054E-s001.pdf]

Electronic Supplementary Material (ESI) for Chemical Science.
This journal is © The Royal Society of Chemistry 2020

Supporting Information for

Annulative π-Extension of BODIPYs Made Easy *via* Gold(I)-Catalyzed Cycloisomerization

Jorge Labella,[a] Gonzalo Durán-Sampedro,[a] M. Victoria Martínez-Díaz,*[ab] Tomás Torres,*[abc]

[a] Departamento de Química Orgánica, Universidad Autónoma de Madrid, 28049 Madrid, Spain.

[b] Institute for Advanced Research in Chemical Sciences (IAdChem), Universidad Autónoma de Madrid, 28049 Madrid, Spain

[c] IMDEA-Nanociencia, Campus de Cantoblanco, 28049 Madrid, Spain

*e-mail: tomas.torres@uam.es, victoria.martinez@uam.es

Table of content

1. Instrumentation and materials S2
2. Synthetic Procedures and Characterization of Obtained Products S3
   Synthesis and characterization of boronic acid **e** S3
   Synthesis and characterization of BODIPYs **1a-g** S3
   Synthesis and characterization of BODIPYs **2a-g** S7
3. X-Ray Crystal Structures and Crystallographic Details S11
   Crystal structures of BODIPYs **1a**, **1b**, **1c**, **1d**, **1e** and **1g** S11
   Crystal structures of BODIPYs **2a, 2d**, **2e**, **2f** and **2g**. S11
   Crystal packing of BODIPYs **2a**, **2e**, **2f**, and **2g**. S12
   Crystallographic details of BODIPYs **1a**, **1b**, **1c**, **1d**, **1e** and **1g** S13
4. UV/Vis Absorption and Emission Spectra S22
5. NMR spectra S24
6. Computational Studies S47
   Origin of **2d** deborylation S47
   NICS(0) calculations S48
   TD-DFT calculations S48
   Cartesian coordinates of **2a**, **2e** and **2g** S50
7. References S71

1. Instrumentation and materials

Nuclear magnetic resonance spectra (^{1}H-, ^{13}C-, ^{19}F-NMR) were recorded on a Bruker AV-300 or a Bruker DRX-500 spectrometers either in the Organic Chemistry Department or in the Interdepartmental Investigation Service of UAM. Deuterated solvent employed in each case is indicated in brackets, and its residual peak was used to calibrate the spectra using literature reference δ ppm values.[1] All the experiments were recorded at room temperature.

Mass spectra (MS) and high resolution mass spectra (HRMS) were recorded in the Interdepartmental Investigation Service of UAM, employing Electronic Impact (EI), or Matrix-Assisted Laser Desorption/Ionization Time-Of-Flight (MALDI-TOF), using a VG-AutoSpec spectrometer for EI, and a Bruker-Ultraflex-III spectrometer, with a Nd:YAG laser operating at 355 nm, for MALDI-TOF. The matrixes and internal references employed are indicated for each spectrum. Infrared Spectra were recorder in solid state on a Bruker Vector 22 spectrophotometer.

Ultraviolet and visible (UV-Vis) spectra were recorded using solvents in the spectroscopic grade in the Organic Chemistry Department of UAM employing a JASCO-V660 spectrophotometer. The logarithm of the molar extinction coefficient (ε) is indicated in brackets for each maximum. Likewise, fluorescence measurements were carried out with a JASCO-V8600 spectrofluorometer.

Fluorescence quantum yields (ϕ_F) of **2a-2g** were determined in DCM and calculated by using the following equation:[2]

$$\phi_F^S = \phi_F^R \left(\frac{\mathrm{Grad_S}}{\mathrm{Grad_R}}\right)\left(\frac{\eta_S}{\eta_R}\right)^2$$

Scripts R and S indicate reference and sample, respectively. Grad is the gradient from the plot of the integrated fluorescence intensity versus the absorption and η is the refractive index of the solvent (the excitation wavelength and the reference compounds are indicated in each case). For **1a-1g** , **2d´**and **2e** rhodamine 6G was used as the standard (Φ_F = 0.95 in ethanol).[3] For BODIPY **2a-2f** and **2g**, zinc phthalocyanine was used as the standard (Φ_F = 0.45 in PrOH).[3]

Single-crystal X-ray diffraction data collection for structure determinations were collected in the Interdepartmental Investigation Service [4] of UAM at Bruker KAPPA APEX II CCD area-detector X-ray diffractometer operating with graphite monochromated and Mo Kalpha radiation (λ = 0.71073 Å). The data are absorption corrected with the program SADABS. Intensities are calculated with the SAINT software, which also incorporates polarization and Lorentz effect corrections. The structures were solved and refined using the Bruker SHELXTL Software Package.

The monitoring of the reactions has been carried out by thin layer chromatography (TLC), employing aluminium sheets coated with silica gel type 60 F254 (0.2 mm thick, Merck). The analysis of the TLCs was carried out with an UV lamp of 254 and 365 nm. Purification and separation of the synthesized products was performed by normal-phase column chromatography, using silica gel (230-400 mesh, 0.040-0.063 mm, Merck). Eluents along with the relative ratio in the case of solvent mixtures are indicated for each particular case.

Chemicals were purchased from commercial suppliers and used without further purification. Dry solvents were purchased from commercial suppliers in anhydrous grade or thoroughly dried before use employing standard methods. Solid, hygroscopic reagents were dried in a vacuum oven before use.

The synthesis and characterization of boronic acids **a-d**, **2,6-Br$_2$BDP**, **2-BrBDP**, **3,5-Cl$_2$BDP** and 1-bromo-2-[2-(trimethylsilyl)ethynyl]- naphthalene **f** have been previously reported.[5]

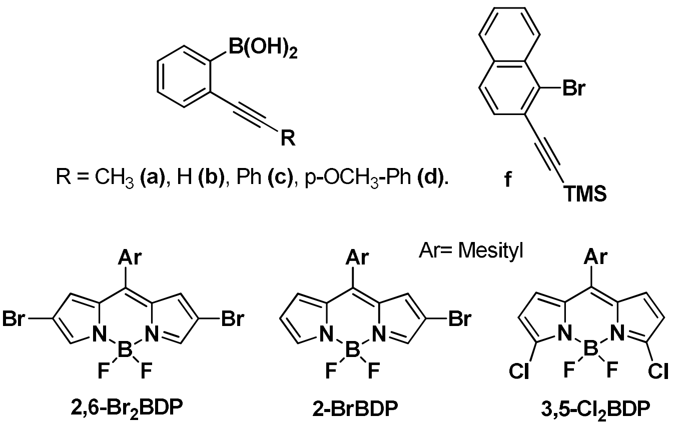

2. Synthetic Procedures and Compound Data

Synthesis and characterization of boronic acid **e**

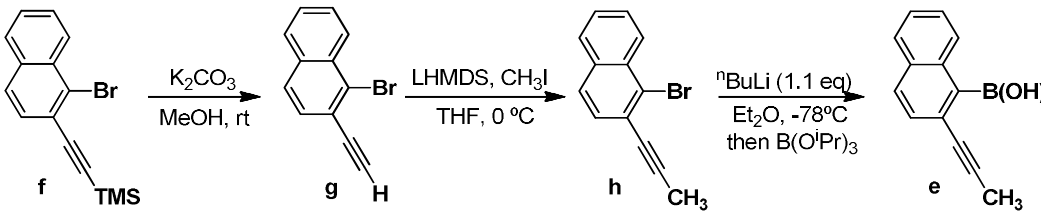

Desilylation: 1-bromo-2-[2-(trimethylsilyl)ethynyl]- naphthalene **f** (3.6 mmol, 1 eq), K_2CO_3 (3.96 mmol, 1.1 eq) were dissolved in 7 mL of MeOH at room temperature for 1h. The reaction mixture was then filtered, concentrated and purified by a short column chromatography on silica gel using PE as eluent to give **g** in 86% yield. *Methylation*: A solution of LHMDS in THF (4.16 mmol, 1.5 eq) was added to a solution of x (2.77 mol, 1 eq) in THF (15 mL) at 0ºC. After 1h iodomethane (4.16 mmol, 1.5 eq) was added at the same temperature and stirred overnight. The mixture was quenched with sat. NH_4Cl aq solution (7 mL), extracted with Et_2O (3x 10 mL), dried over $MgSO_4$, filtered and concentrated. The residue was purified by a short column chromatography on silica gel using PE to give **h** in 77% yield. *Borylation*: A 1.6 M solution of ^nBuLi in hexanes (2.95 mmol, 1.2 eq), was added dropwise to a solution of **h** (2.46 mmol, 1 eq) in THF (40 mL) at – 78 ºC. After 30 min, $B(O^iPr)_3$ (4.92 mmol, 2 eq) was added and reaction mixture was left to stir at – 78ºC gradually warming up to RT over 16 h. The reaction was quenched with 1M HCl aq solution (30 mL), extracted with Et_2O (3x 20 mL), dried over $MgSO_4$, filtered and concentrated. The residue was purified by column chromatography on silica gel using first PE and then PE/Et_2O as eluent to give **e** in 71% yield. **^{1}H-NMR** (300 MHz, $CDCl_3$): δ (ppm) = 8.37 (d, $^3J_{H-H}$ = 9 Hz, 1H), 7.80 (d, $^3J_{H-H}$ = 9 Hz, 2H), 7.48 (m, 3H), 5.53 (s, 2H), 2.13 (s, 3H); **^{13}C-NMR** (75.50 MHz, $CDCl_3$): δ (ppm) = 135.64, 132.50, 130.02, 129.05, 128.53, 128.46, 126.97, 126.35, 125.72, 90.09, 81.13, 4.67; **HRLSI-MS (ESI+)**: Calculated for 233.0750 ; Found 233.0743, ppm error = 3.0. ; **FT-IR**: ν (cm^{-1}) = 3300, 3050, 1442, 800. **Mp** = 155 – 158 ºC

Synthesis and characterization of BODIPYs **1a-g**

General method for the Suzuki cross-coupling.[4b]

In a round-bottom flask **2,6-Br$_2$BDP** (0.11 mmol, 1 eq), the appropriate boronic acid (0.26 mmol, 2.4 eq), $Pd_2(dba)_3.CHCl_3$ (11 μmol, 0.1 eq), *t*-$Bu_3P.HBF_4$ (44 μmol, 0.4 eq) and $CsCO_3$ (0.44 mmol, 4 eq) were dissolved in 6 ml of a previously degassed mixture of THF/water (15:1; v/v) under argon

atmosphere. The reaction was stirred at room temperature overnight. After that, the crude of the reaction was extracted with DCM (3x20 mL), dried over $MgSO_4$, filtered and concentrated. The residue was purified by column chromatography on silica gel using a suitable eluent, as specified in each case.

BODIPY **1a**

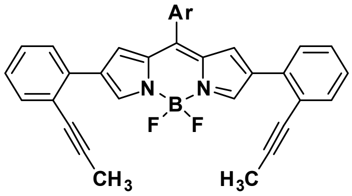

BODIPY **1a** was prepared following the general method described above and purified by column chromatography on silica gel using heptane/AcOEt as eluent (from 50:1 to 5:1; v/v). The product obtained from the column was further purified washing with cold hexane. **1a** was isolated as a purple solid in 93% yield. **^{1}H-NMR** (300 MHz, $CDCl_3$): δ (ppm) = 8.46 (s, 2H), 7.42 (m, 4H), 7.26 - 7.18 (m, 4H), 7.12 (s, 2H), 6.98 (s, 2H), 2.38 (s, 3H), 2.22 (s, 6H), 1.98 (s, 6H); **^{13}C-NMR** (75.5 MHz, $CDCl_3$): δ (ppm) = 146.87, 144.37, 139.07, 136.69, 135.63, 134.41, 133.59, 133.26, 130.07, 128.25, 128.09, 127.52, 127.52, 127.22, 121.38, 90.76, 79.91, 32.32, 21.30, 20.32; **^{19}F-NMR** (282 MHz, $CDCl_3$): δ (ppm) = -145.86 (m); **^{11}B-NMR** (128 MHz, $CDCl_3$): δ (ppm) = 0.35 (t, $J_{B\text{-}F}$ = 29 Hz); **HRLSI-MS** (MALDI-TOF): Calculated for $C_{36}H_{29}BF_2N_2$: 538,2393; Found: 538.2404, ppm error = 2.0. ; **UV/vis (DCM)**: λ_{max} (nm) (log ε) = 573 (4.29), 328 (4.45). **Φ_F** (exc. λ = 550 nm) = 0.52. **FT-IR**: ν (cm^{-1}) = 3041, 3027, 2967, 2870, 2841, 1560, 1427, 1392, 1231, 1101, 979, 781. **Mp** > 200 ºC.

BODIPY **1b**

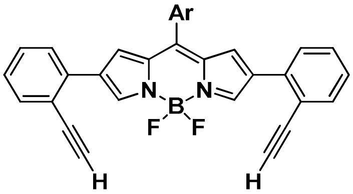

BODIPY **1b** was prepared following the general method described above and purified by column chromatography on silica gel using heptane/AcOEt as eluent (from 50:1 to 5:1; v/v). The product obtained from the column was further purified washing with cold hexane. **1b** was isolated as a purple solid in 62% yield. **^{1}H-NMR** (300 MHz, $CDCl_3$): δ (ppm) = 8.49 (s, 2H), 7.56 (dd, $^3J_{H\text{-}H}$ = 7.5 Hz, $^4J_{H\text{-}H}$ = 1.5 Hz, 2H), 7.41 (dd, $^3J_{H\text{-}H}$ = 7.5 Hz, $^4J_{H\text{-}H}$ = 1.5 Hz, 2H), 7.33 (td, $^3J_{H\text{-}H}$ = 7.5 Hz, $^4J_{H\text{-}H}$ = 1.5 Hz, 2H), 7.23 (m, 2H), 7.06 (s, 2H), 6.98 (s, 2H), 3.23 (s, 2H), 2.38 (s, 3H), 2.20 (s, 6H); **^{13}C-NMR** (75.5 MHz, $CDCl_3$): δ (ppm) = 13C NMR (75 MHz, CDCl3) δ 144.29, 139.00, 136.47, 135.55, 135.09, 134.34, 132.67, 132.61, 129.58, 129.18, 128.24, 127.79, 127.63, 127.19, 119.40, 83.32, 81.70, 21.21, 20.16.; **^{19}F-NMR** (282 MHz, $CDCl_3$): δ (ppm) = -145.84 (m); **^{11}B-NMR** (128 MHz, $CDCl_3$): δ (ppm) = 0.37 (t, $J_{B\text{-}F}$ = 29 Hz); **HRLSI-MS** (MALDI-TOF): Calculated for $C_{34}H_{25}BF_2N_2$: 510.2079; Found 510.2088. , ppm error = 1.8.; **UV/vis (DCM)**: λ_{max} (nm) (log ε) = 565 (4.1). **Φ_F** (exc. λ = 550 nm) = 0.54. **FT-IR**: ν (cm^{-1}) = 3252, 3031, 2969, 2869, 1560, 1398, 1222, 1103, 983, 784. **Mp** > 200 ºC.

BODIPY **1c**

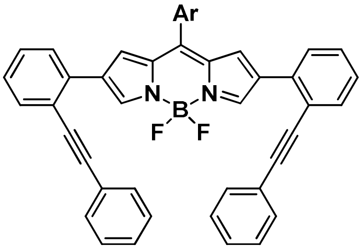

BODIPY **1c** was prepared following the general method described above and purified by column chromatography on silica gel using heptane/AcOEt as eluent (from 50:1 to 15:1; v/v). The product obtained from the column was further purified washing with cold hexane. **1c** was isolated as a purple solid in 91% yield. **^{1}H-NMR** (300 MHz, $CDCl_3$): δ (ppm) = 8.48 (s, 2H), 7.51 (d, $^3J_{H-H}$ = 9 Hz, 2H), 7.38 (d, $^3J_{H-H}$ = 9 Hz, 2H), 7.29 – 7.12 (m), 6.78 (s, 2H), 2.25 (s, 3H), 2.03 (s, 6H). **^{13}C-NMR** (75.5 MHz, $CDCl_3$): δ (ppm) = 147.36, 144.58, 144.48, 138.78, 136.55, 135.88, 134.43, 134.34, 133.92, 133.17, 131.59, 129.81, 128.52, 128.46, 127.72, 127.34, 123.10, 120.61, 93.85, 89.36, 21.37, 20.34.; **^{19}F-NMR** (282 MHz, $CDCl_3$): δ (ppm) = -145.64 (m); **^{11}B-NMR** (128 MHz, $CDCl_3$): δ (ppm) = 0.39 (t, J_{B-F} = 29 Hz); **HRLSI-MS** (MALDI-TOF): Calculated for $C_{46}H_{33}BF_2N_2$ 662.2707; Found 662.2691, ppm error = 2.4.; **UV/vis (DCM)**: λ_{max} (nm) (log ε) = 578 (4.3). **Φ_F** (exc. λ = 550 nm) = 0.50. **FT-IR**: ν (cm^{-1}) = 3098, 3030, 2950, 2820, 1562, 1420, 1392, 1227, 1097, 977, 784, 702. **Mp** > 200 ºC.

BODIPY **1d**

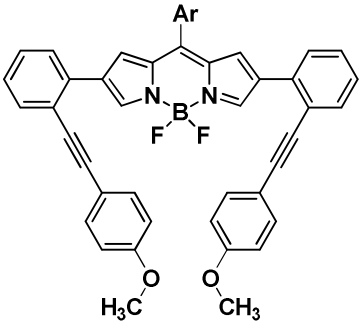

BODIPY **1d** was prepared following the general method described above and purified by column chromatography on silica gel using heptane/AcOEt as eluent (from 50:1 to 5:1; v/v). The product obtained from the column was further purified washing with cold hexane. **1d** was isolated as a purple solid in 83% yield. **^{1}H-NMR** (300 MHz, $CDCl_3$): δ (ppm) = 8.61 (s, 2H), 7.57 (dd, $^3J_{H-H}$ = 6 Hz, $^4J_{H-H}$ = 1.2 Hz, 2H), 7.46 (dd, $^3J_{H-H}$ = 6 Hz, $^4J_{H-H}$ = 1.2 Hz, 2H), 7.36 - 7.21 (m), 7.18 (s, 2H), 6.90 - 6.86 (m, 6H), 3.85 (s, 6H), 2.37 (s, 3H), 2.15 (s, 6H); **^{13}C-NMR** (75.5 MHz, $CDCl_3$): δ (ppm) = 159.94, 147.12, 144.53, 138.77, 136.63, 135.84, 134.11, 133.68, 133.30, 133.27, 133.10, 129.90, 128.45, 127.70, 127.32, 127.18, 120.98, 115.27, 114.16, 94.05, 88.20, 55.47, 21.40, 20.38; **^{19}F-NMR** (282 MHz, $CDCl_3$): δ (ppm) = -145.66 (m); **^{11}B-NMR** (128 MHz, $CDCl_3$): δ (ppm) = 0.39 (t, J_{B-F} = 29 Hz); **HRLSI-MS** (MALDI-TOF): Calculated for $C_{48}H_{37}BF_2N_2O_2$: 722.2919; Found 722.2886, ppm error = 4.6.; **UV/vis (DCM)**: λ_{max} (nm) (log ε) = 583 (4.4). **Φ_F** (exc. λ = 550 nm) = 0.44. **FT-IR**: ν (cm^{-1}) = 3073, 3012, 2977, 2860, 1561, 1419, 1396, 1299, 1226, 1097, 1012, 779, 707. **Mp** > 200 ºC.

BODIPY **1e**

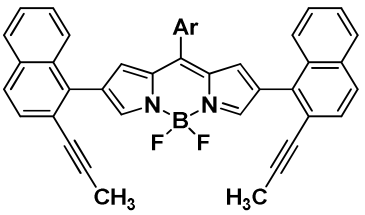

BODIPY **1e** was prepared following the general method described above and purified by column chromatography on silica gel using heptane/AcOEt as eluent (from 50:1 to 10:1; v/v). The product obtained from the column was further purified washing with cold hexane. **1e** was isolated as a purple solid in 75% yield. **^{1}H-NMR** (300 MHz, $CDCl_3$): δ (ppm) = 8.30 (s, 2H), 7.98 - 7.94 (m, 2H), 7.85 – 7.82 (m, 2H), 7.75 (d, $^{3}J_{H-H}$ = 9 Hz, 2H), 7.55 – 7.48 (m, 6H), 7.02 (s, 2H), 6.98 (s, 2H), 2.35 (s, 3H), 2.34 (s, 6H), 2.04 (s, 6H); **^{13}C-NMR** (75.5 MHz, $CDCl_3$): δ (ppm) = 146.38, 139.10, 136.51, 133.38, 133.06, 131.93, 130.89, 130.03, 129.32, 128.42, 128.38, 127.85, 126.99, 126.36, 126.05, 125.29, 121.19, 91.06, 80.23, 21.26, 20.45, 4.68; **^{19}F-NMR** (282 MHz, $CDCl_3$): δ (ppm) = -145.86 (m); **^{11}B-NMR** (128 MHz, $CDCl_3$): δ (ppm) = 0.47 (t, J_{B-F} = 28 Hz); **HRLSI-MS** (MALDI-TOF): Calculated for $C_{44}H_{33}BF_2N_2$: 638.2707; Found 638.2715, ppm error = 1.3.; **UV/vis (DCM)**: λ_{max} (nm) (log ε) = 560 (4.6). **Φ_F** (exc. λ = 550 nm) = 0.49. **FT-IR**: ν (cm^{-1}) = 3040, 3028, 2967, 2841, 1561, 1422, 1392, 1234, 1098, 960, 781. **Mp** > 200 ºC.

<u>BODIPY **1f**</u>

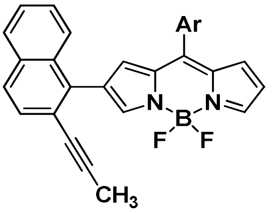

In a round-bottom flask **2-BrBDP** (0.11 mmol, 1 eq), boronic acid **e** (0.13 mmol, 1.2 eq), $Pd_2(dba)_3.CHCl_3$ (11 μmol, 0.1 eq), *t*-$Bu_3P.HBF_4$ (44 μmol, 0.4 eq) and $CsCO_3$ (0.44 mmol, 4 eq) were dissolved in 6 ml of a previously degassed mixture of THF/water (15:1; v/v) under argon atmosphere. The reaction was stirred at room temperature overnight. After that, the crude of the reaction was extracted with DCM (3x20 mL), dried over $MgSO_4$, filtered and concentrated. The residue was purified by column chromatography on silica gel using heptane/AcOEt as eluent (from 50:1 to 20:1; v/v). The product obtained from the column was further purified washing with cold hexane. **1f** was isolated as a purple solid in 94 % yield. **^{1}H-NMR** (300 MHz, $CDCl_3$): δ (ppm) = 8.24 (s, 1H), 7.94 (s, 1H), 7.94 -7.87 (m, 1H), 7.82 – 7.78 (m, 1H), 7.71 (d, $^{3}J_{H-H}$ = 9 Hz, 1H), 7.50 – 7.44 (m, 3H), 6.96 (bs, 3H), 6.96 (d, $^{3}J_{H-H}$ = 3 Hz, 1H), 6.50 (dd, $^{3}J_{H-H}$ = 3 Hz, $^{3}J_{H-H}$ = 1.7 Hz, 1H), 2.34 (s, 3H), 2.21 (s, 6H), 1.98 (s, 3H); **^{13}C-NMR** (75.5 MHz, $CDCl_3$): δ (ppm) = 147.29, 146.91, 143.87, 139.06, 136.50, 135.52, 133.27, 133.04, 131.89, 131.22, 130.85, 129.97, 129.88, 129.26, 128.40, 128.36, 127.88, 126.99, 126.36, 126.00, 121.22, 118.47, 118.43, 91.12, 80.16, 21.26, 20.27, 4.60.; **^{19}F-NMR** (282 MHz, $CDCl_3$): δ (ppm) = -145.66 (m); **^{11}B-NMR** (128 MHz, $CDCl_3$): δ (ppm) = 0.43 (t, J_{B-F} = 29 Hz); **HRLSI-MS** (MALDI-TOF): Calculated for $C_{31}H_{25}BF_2N_2$: 474.2079; Found 474.2070, ppm error = 4.4.; **UV/vis (DCM)**: λ_{max} (nm) (log ε) = 523 (4.59). **Φ_F** (exc. λ = 550 nm) = 0.54. **FT-IR**: ν (cm^{-1}) = 3063, 3047, 2897, 1536, 1506, 1400, 1139, 1100, 821, 795. **Mp** > 200 ºC.

<u>BODIPY **1g**</u>

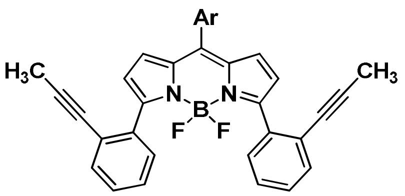

In a round-bottom flask **3,5-Cl$_2$BDP** (0.11 mmol, 1 eq), boronic acid **a** (0.13 mmol, 1.2 eq), Pd(PPh$_3$)$_4$ (11 μmol, 0.1 eq) and CsCO$_3$ (0.44 mmol, 4 eq) were dissolved in 6 ml of a previously degassed mixture of toluene/water (10:1; v/v) under argon atmosphere. The reaction was stirred and heated to reflux overnight. After that, the crude of the reaction was extracted with toluene (3x20 mL), dried over MgSO$_4$, filtered and concentrated. The residue was purified by column chromatography on silica gel using heptane/AcOEt as eluent (from 50:1 to 10:1; v/v). The product obtained from the column was further purified washing with cold hexane. BODIPY **X** was isolated as a red solid in 44 % yield. **^{1}H-NMR** (300 MHz, CDCl$_3$): δ (ppm) = 7.90 (d, $^{3}J_{H-H}$ = 9 Hz, 2H), 7.43 (d, $^{3}J_{H-H}$ = 9 Hz, 2H), 7.34 – 7.26 (m), 6.99 (s, 2H), 6.71 (d, $^{3}J_{H-H}$ = 6 Hz, 2H), 6.63 (d, $^{3}J_{H-H}$ = 6 Hz, 2H), 2.39 (s, 3H), 2.24 (s, 6H), 1.91 (s, 6H); **^{13}C-NMR** (75.5 MHz, CDCl$_3$): δ (ppm) = 157.41, 144.00, 138.61, 136.96, 136.01, 134.75, 132.59, 130.70, 130.63, 130.56, 128.89, 128.26, 128.16, 127.26, 123.57, 122.50, 122.46, 90.54, 79.23, 21.30, 20.35, 4.65; **^{19}F-NMR** (282 MHz, CDCl$_3$): δ (ppm) = -145.56 (m); **^{11}B-NMR** (128 MHz, CDCl$_3$): δ (ppm) = 1.16 (t, J_{B-F} = 31 Hz); **HRLSI-MS** (MALDI-TOF): Calculated for $C_{36}H_{29}BF_2N_2$: 538,2393; Found: 538.2414, ppm error = 3.9.; **UV/vis (DCM)**: λ_{max} (nm) (log ε) = 545 (4.3). **Φ_F** (exc. λ = 550 nm) = 0.87. **FT-IR**: ν (cm^{-1}) = 3063, 2897, 1550, 1413, 1135, 1107, 1092, 798. **Mp** > 200 ºC.

<u>Synthesis and characterization of BODIPYs **2a-g**</u>

General method for the gold-catalyzed cycloisomerization

In a Schlenk tube the corresponding alkynyl-aryl BODIPY (0.04 mmol, 1 eq), P(C$_6$F$_5$)$_3$AuCl (4 μmol, 0.1 eq), AgSbF$_6$ (4 μmol, 0.1 eq) were dissolved in DCM (2 mL) under argon atmosphere. The reaction was stirred at room temperature for 15 min. After that, the crude of the reaction was extracted with DCM (3x10 mL), dried over MgSO$_4$, filtered and concentrated. The residue was purified by column chromatography on silica gel using toluene as eluent. The product obtained from the column was further purified washing with cold hexane. The yield of the reaction and other details are specified in each case.

<u>BODIPY **2a**</u>

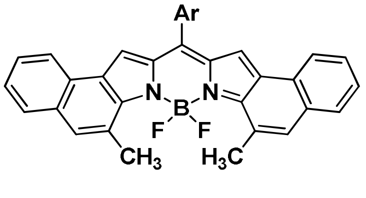

BODIPY **2a** was prepared following the general method described above and purified by column chromatography on silica gel using toluene as eluent. The product obtained from the column was further purified washing with cold hexane. **2a** was isolated quantitatively as a blue solid. **^{1}H-NMR** (300 MHz, CDCl$_3$): δ (ppm) = 7.97 – 7.94 (m, 2H), 7.69 - 7.66 (m, 2H), 7.58 (s, 2H), 7.43 - 7.38 (m, 6H), 7.09 (s, 2H), 3.01 (d, $^{4}J_{H-H}$ = 3 Hz, 6H), 2.49 (s, 3H), 2.21 (s, 6H); **^{13}C-NMR** (75.5 MHz, CDCl$_3$): δ (ppm) = 151.57, 146.63, 138.99, 138.92, 137.26, 136.65, 131.41, 131.06, 130.28, 128.29, 128.26, 127.47, 127.37, 127.17, 126.35, 123.40, 121.43, 21.28, <u>20.79 (t, J_{C-F} = 11 Hz)</u>, 20.34; **^{19}F-**

NMR (282 MHz, $CDCl_3$): δ (ppm) = -123.13 (m); **^{11}B-NMR** (128 MHz, $CDCl_3$): δ (ppm) = 1.77 (t, J_{B-F} = 34 Hz); **HRLSI-MS** (MALDI-TOF): Calculated for $C_{36}H_{29}BF_2N_2$: 538,2393; Found: 538.2409, ppm error = 3.0; **UV/vis (DCM)**: λ_{max} (nm) (log ε) = 650 (5.1); Φ_F (exc. λ = 630 nm) = 0.08. **FT-IR**: ν (cm^{-1}) = 3098, 3045, 3030, 2888, 2962, 1542, 1412, 1392, 1387, 1215, 1097, 977, 801. **Mp** > 200 ºC.

BODIPY **2b**

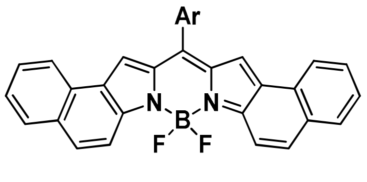

BODIPY **2b** was prepared following the general method described above and purified by column chromatography on silica gel using toluene as eluent. The product obtained from the column was further purified washing with cold hexane. **2b** was isolated quantitatively as a blue solid. **^{1}H-NMR** (300 MHz, $CDCl_3$): δ (ppm) = 7.99 (d, $^3J_{H-H}$ = 9 Hz, 2H), 7.91 (d, $^3J_{H-H}$ = 9 Hz, 2H), 7.79 (d, $^3J_{H-H}$ = 9 Hz, 4H), 4.47 (m, 4H), 7.33 (s, 2H), 7.10 (s, 2H), 2.48 (s, 3H), 2.22 (s, 6H); **^{13}C-NMR** (75.5 MHz, $CDCl_3$): δ (ppm) = 151.09, 147.57, 139.31, 137.32, 135.75, 130.57, 130.26, 130.04, 129.89, 129.19, 128.71, 128.48, 128.38, 126.53, 124.07, 121.33, 116.63, 21.43, 20.54; **^{19}F-NMR** (282 MHz, $CDCl_3$): δ (ppm) = - 146.44 (m); **^{11}B-NMR** (128 MHz, $CDCl_3$): δ (ppm) = 1.82 (t, J_{B-F} = 34 Hz); **HRLSI-MS** (MALDI-TOF): Calculated for $C_{34}H_{25}BF_2N_2$: 510.2079; Found: 510.2094, ppm error = 2.9; **UV/vis (DCM)**: λ_{max} (nm) (log ε) = 650 (5.3); Φ_F (exc. λ = 630 nm) = 0.09. **FT-IR**: ν (cm^{-1}) = 3086, 2937, 1554, 1389, 1185, 1103, 959, 802. **Mp** > 200 ºC.

BODIPY **2c**

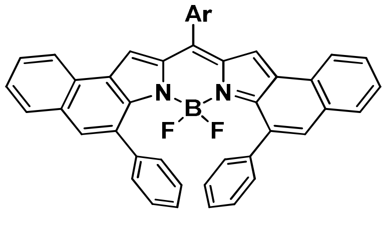

In a Schlenk tube the BODIPY **1c** (0.04 mmol, 1 eq), $P(C_6F_5)_3AuCl$ (8 μmol, 0.2 eq), $AgSbF_6$ (8 μmol, 0.2 eq) were dissolved in DCM (2 mL) under argon atmosphere. The reaction was stirred at 40°C for 1 h . After that, the crude of the reaction was extracted with DCM (3x10 mL), dried over $MgSO_4$, filtered and concentrated. The residue was purified by column chromatography on silica gel using toluene as eluent. The product obtained from the column was further purified washing with cold hexane. **2c** was isolated in a 78% yield as a purple solid. **^{1}H-NMR** (500 MHz, $CDCl_3$): δ (ppm) = 7.99 (d, $^3J_{H-H}$ = 8 Hz, 2H), 7.63 (d, $^3J_{H-H}$ = 8 Hz, 2H), 7.47 - 7.22 (m), 7.10 (s, 2H), 2.49 (s, 3H), 2.21 (s, 6H); **^{13}C-NMR** (126MHz, $CDCl_3$): δ (ppm) = 149.98, 147.50, 139.70, 139.56, 139.18, 138.45, 137.45, 132.52, 131.54, 131.30, 129.69, 129.55, 129.10, 128.42, 127.99, 127.98, 127.75, 127.14, 126.63, 123.57, 121.63, 21.45, 20.45; **^{19}F-NMR** (470 MHz, $CDCl_3$): δ (ppm) = - 129.38 (m); **^{11}B-NMR** (128 MHz, $CDCl_3$): δ (ppm) = 1.02 (t, J_{B-F} = 35 Hz); **HRLSI-MS** (MALDI-TOF): Calculated for $C_{46}H_{33}BF_2N_2$: 662.2707; Found 662.2717, ppm error = 1.5; **UV/vis (DCM)**: λ_{max} (nm) (log ε) = 650 (5.1); Φ_F (exc. λ = 630 nm) = 0.07. **FT-IR**: ν (cm^{-1}) = 3090, 3025, 3015, 2937, 1544, 1399, 1396, 1185, 1097, 977, 801, 715. **Mp** > 200 ºC.

Dipyrromethene **2d′**

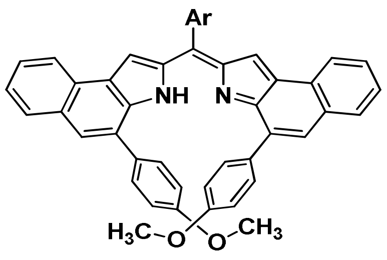

Dipyrromethene **2d′** was prepared following the general method described above and purified by column chromatography on silica gel using toluene as eluent. The product obtained from the column was further purified washing with cold hexane. **2d′** was isolated quantitatively as a purple solid. **^{1}H-NMR** (300 MHz, $CDCl_3$): δ (ppm) = 12.97 (s, 1H), 7.96 – 7.85 (m, 2H), 7.84 (d, $^{3}J_{H\text{-}H}$ = 9 Hz, 4H), 7.72 – 7.69 (m, 2H), 7.51 (s, 2H), 7.40 (m, 4H), 7.20 (s, 2H), 7.11 (s, 2H), 6.71 (d, $^{3}J_{H\text{-}H}$ = 9 Hz, 4H), 3.85 (s, 6H), 2.85 (s,3H), 2.26 (s, 6H); **^{13}C-NMR** (75.50 MHz, $CDCl_3$): δ (ppm) = 159.94, 147.12, 144.53, 138.77, 136.63, 135.84, 134.11, 133.68, 133.30, 133.27, 133.10, 129.90, 128.45, 127.70, 127.32, 127.18, 120.98, 115.27, 114.16, 94.05, 88.20, 55.47, 21.40, 20.38; **HRLSI-MS** (MALDI-TOF): Calculated for $C_{48}H_{38}N_2O_2$: 674.2928; Found 674.2933, ppm error = 0.7; **UV/vis (DCM)**: λ_{max} (nm) (log ε) = 586 (4.6). **Φ_F** (exc. λ = 550 nm) = 0.01. **FT-IR**: ν (cm^{-1}) = 3497, 3035, 2987, 1421, 1399, 1396, 1185, 1097, 801, 725. **Mp** > 200 ºC.

<u>BODIPY **2e**</u>

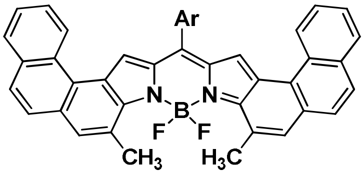

BODIPY **2e** was prepared following the general method described above and purified by column chromatography on silica gel using toluene as eluent. The product obtained from the column was further purified washing with cold hexane. **2e** was isolated quantitatively as a blue solid. **^{1}H-NMR** (500 MHz, $CDCl_3$): δ (ppm) = 8.59 (d, $^{3}J_{H\text{-}H}$ = 10 Hz, 2H), 7.95 (s, 2H), 7.92 (d, $^{3}J_{H\text{-}H}$ = 10 Hz, 2H), 7.79 (d, $^{3}J_{H\text{-}H}$ = 10 Hz, 2H), 7.72 – 7.69 (m, 4H), 7.62 – 7.54 (m, 4H), 7.17 (s, 2H), 3.10 (s, 6H), 2.55 (s, 3H), 2.28 (s, 6H); **^{13}C-NMR** (126 MHz, $CDCl_3$): δ (ppm) = 153.18, 148.52, 139.82, 139.33, 138.38, 137.40, 133.64, 130.86, 130.82, 130.74, 129.61, 129.38, 128.72, 127.57, 127.46, 127.32, 127.25, 126.11, 125.67, 125.33, 124.04, 21.58, <u>21.03 (t, $J_{C\text{-}F}$ = 11 Hz)</u>, 20.73. **^{19}F-NMR** (470 MHz, $CDCl_3$): δ (ppm) = - 122.22 (m); **^{11}B-NMR** (128 MHz, $CDCl_3$): δ (ppm) = 1.83 (t, $J_{B\text{-}F}$ = 34 Hz); **HRLSI-MS** (MALDI-TOF): Calculated for $C_{44}H_{33}BF_2N_2$: 638.2707; Found: 638.2718, ppm error = 1.7.; **UV/vis (DCM)**: λ_{max} (nm) (log ε) = 660 (5.0); Φ_F (exc. λ = 630 nm) = 0.00. **FT-IR**: ν (cm^{-1}) = 3063, 3055, 3022, 3015, 2897, 1547, 1414, 1111, 1102, 1092, 801. **Mp** > 200 ºC.

<u>BODIPY **2f**</u>

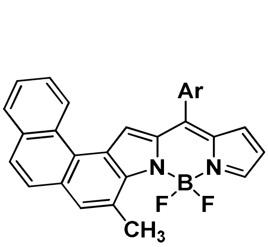

BODIPY **2f** was prepared following the general method described above and purified by column chromatography on silica gel using toluene as eluent. The product obtained from the column was further purified washing with cold hexane. **2f** was isolated quantitatively as a purple solid. **^{1}H-NMR**

(500 MHz, $CDCl_3$): δ (ppm) = 8.58 (d, $^3J_{H-H}$ = 10 Hz, 1H), 8.14 (s, 1H), 7.92 – 7.90 (m, 2H), 7.77 (s, 1H), 7.76 (d, $^3J_{H-H}$ = 10 Hz, 1H), 7.71 -7.69 (m, 2H), 7.58 – 7.51 (m, 2H), 7.07 (s, 2H), 6.81 (d, $^3J_{H-H}$ = 4 Hz, 1H), 6.64 (dd, $^3J_{H-H}$ = 5 Hz, $^3J_{H-H}$ = 1.5 Hz, 1H), 3.02 (s, 3H), 2.46 (s,3H), 2.19 (s, 3H); **^{13}C-NMR** (75.5 MHz, $CDCl_3$): δ (ppm) = 151.04, 148.46, 147.55, 139.25, 138.17, 137.79, 136.88, 136.33, 133.43, 130.99, 130.62, 130.20, 129.26, 129.22, 129.19, 128.58, 127.31, 126.94, 126.87, 126.81, 125.92, 125.33, 123.97, 121.34, 121.28, 21.43, 20.63, (t, J_{C-F} = 10 Hz), 20.41; **^{19}F-NMR** (282 MHz, $CDCl_3$): δ (ppm) = - 133.31 (m); **^{11}B-NMR** (128 MHz, $CDCl_3$): δ (ppm) = 1.32 (t, J_{B-F} = 35 Hz); **HRLSI-MS** (MALDI-TOF): Calculated for $C_{31}H_{25}BF_2N_2$: 474.2079; Found 474.2088, ppm error = 1.9; **UV/vis (DCM)**: λ_{max} (nm) (log ε) = 568 (4.6). Φ_F (exc. λ = 550 nm) = 0.02. **FT-IR**: ν (cm^{-1}) = 3071, 3018, 3010, 2903, 1546, 1430, 1222, 1092, 801, 630, **Mp** > 200 ºC.

BODIPY **2g**

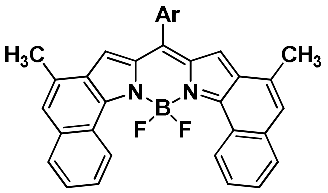

In a Schlenk tube the BODIPY **1g** (0.04 mmol, 1 eq), $P(C_6F_5)_3AuCl$ (8 µmol, 0.2 eq), $AgSbF_6$ (8 µmol, 0.2 eq) were dissolved in DCM (2 mL) under argon atmosphere. The reaction was stirred at room temperature for 5 min . After that, the crude of the reaction was extracted with DCM (3x10 mL), dried over $MgSO_4$, filtered and concentrated. The residue was purified by column chromatography on silica gel using toluene as eluent. The product obtained from the column was further purified washing with cold hexane. **2g** was isolated quantitatively as a purple solid. **^{1}H-NMR** (300 MHz, $CDCl_3$): δ (ppm) = 9. 53 (d, $^3J_{H-H}$ = 9 Hz, 2H), 7.69 – 7.57 (m, 6H), 7.07 (bs, 4H), 6.91 (s, 2H), 2.46 (s, 3H), 2.40 (d, $^4J_{H-H}$ = 3 Hz, 6H), 2.21 (s, 6H); **^{13}C-NMR** (75.50 MHz, $CDCl_3$): δ (ppm) = 150.96, 146.98, 138.93, 138.78, 137.18, 137.05, 133.57, 130.94, 129.62, 129.49, 128.77, 128.62, 128.56, 128.47, 128.25, 126.55, 124.67, 122.84, 122.36, 21.27, 20.35, 18.68; **^{19}F-NMR** (282 MHz, $CDCl_3$): δ (ppm) = - 141.56 (m); **^{11}B-NMR** (128 MHz, $CDCl_3$): δ (ppm) = 2.77 (t, J_{B-F} = 35 Hz); **HRLSI-MS** (MALDI-TOF): Calculated for $C_{36}H_{29}BF_2N_2$: 538,2393; Found: 538.2415, ppm error = 4.0.; **UV/vis (DCM)**: λ_{max} (nm) (log ε) = 683 (4.7), 632 (4.5), 548 (4.4); Φ_F (exc. λ = 630 nm) = 0.01. **FT-IR**: ν (cm^{-1}) = 3075, 3069, 3030, 3019, 2910, 2890, 1517, 1376, 1228, 1096, 972, 799. **Mp** > 200 ºC.

3. X-Ray Crystal Structures and Crystallographic Details

Crystal structures of BODIPYs **1a**, **1b**, **1c**, **1d**, **1e** and **1g**.

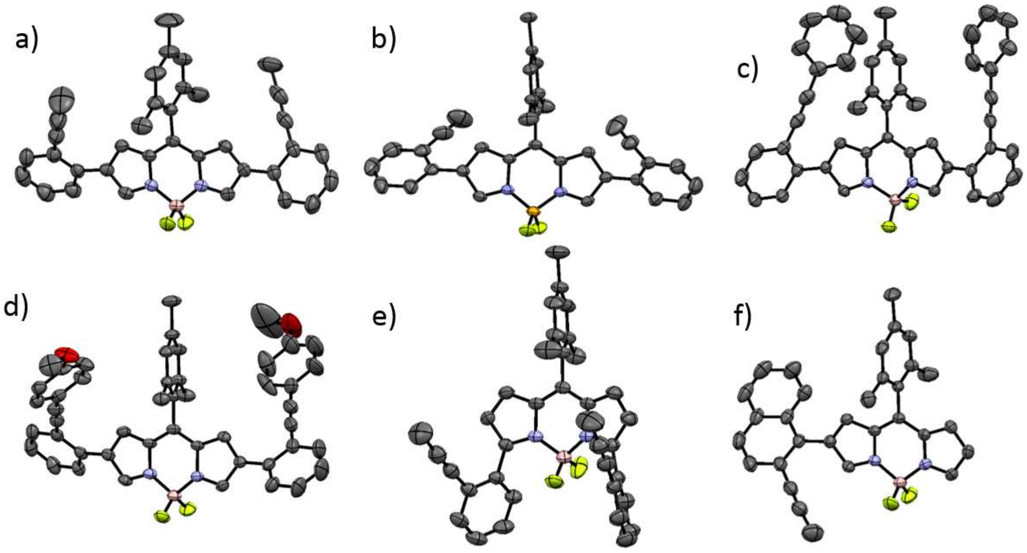

Figure S3.1. X-Ray crystal structures of **1a**, **1b**, **1c**, **1d**, **1e** and **1g**. Thermal ellipsoids are scaled to 50% probability level. Hydrogen atoms are omitted for clarity.

Crystal structures of BODIPYs **2a, 2d**, **2e**, **2f** and **2g**.

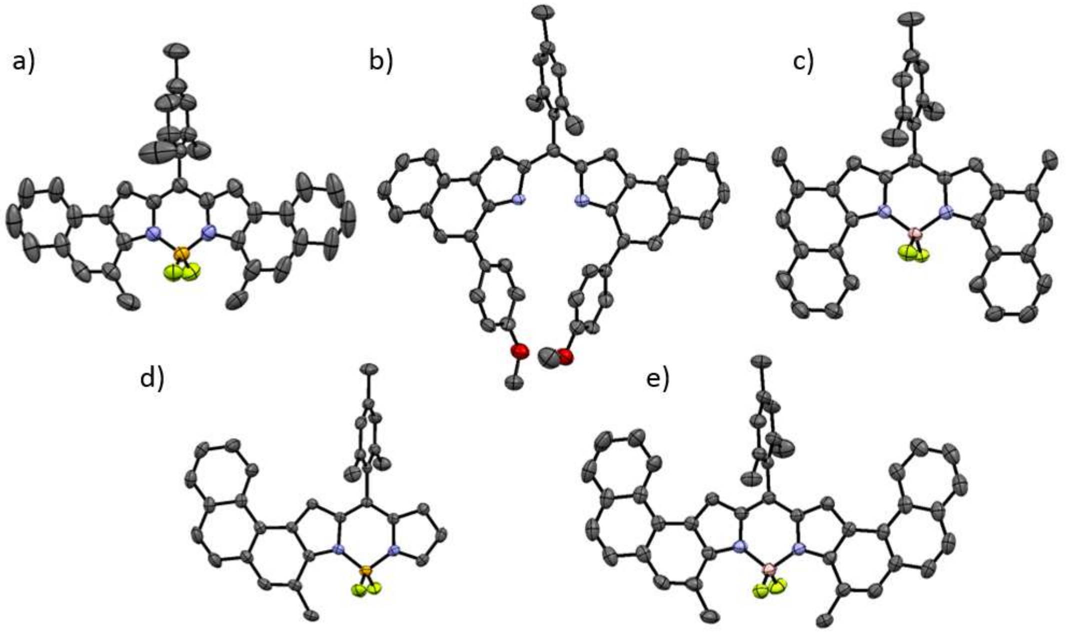

Figure S3.2. X-Ray crystal structures of **2a**, **2d**, **2e**, **2f** and **2g**. Thermal ellipsoids are scaled to 50% probability level. Hydrogen atoms are omitted for clarity.

Crystal packing of BODIPYs **2a**, **2e**, **2f**, and **2g**.

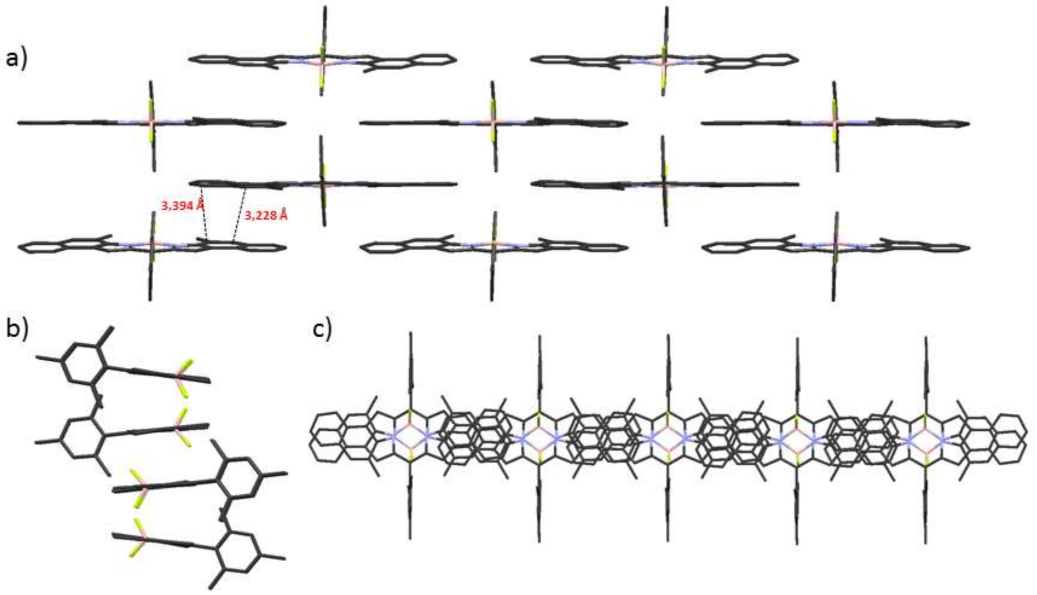

Figure S3.3. Crystal packing of **2a** (a: front view; b: side view; c: top view)

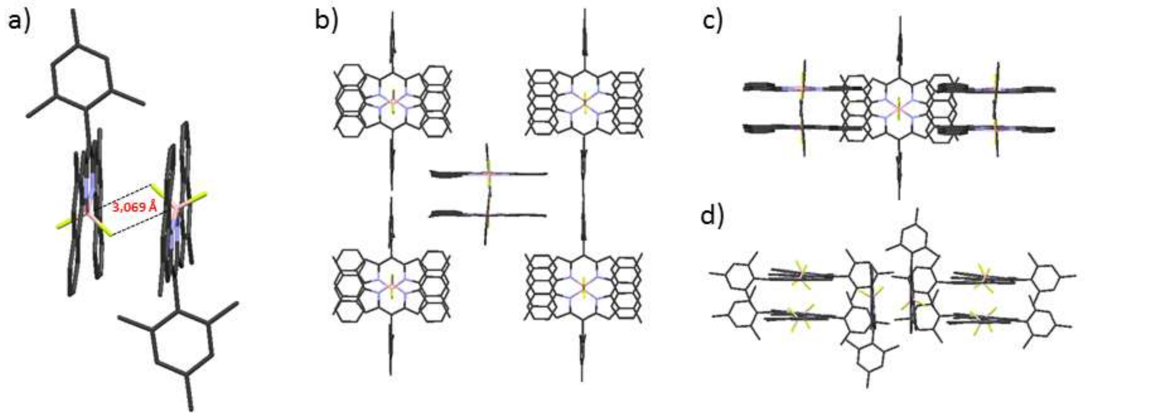

Figure S3.4. Crystal packing of **2g** (a: dimers linked by B-F interactions; b: front view; c: top view; d: side view)

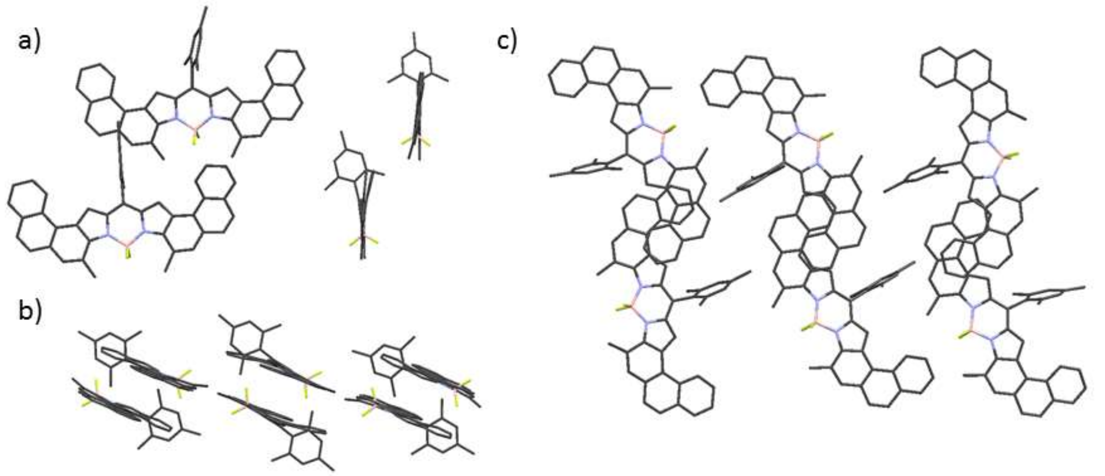

Figure S3.5. Crystal packing of **2e** (a: two unit of the unit cell; b: front view; c: top view)

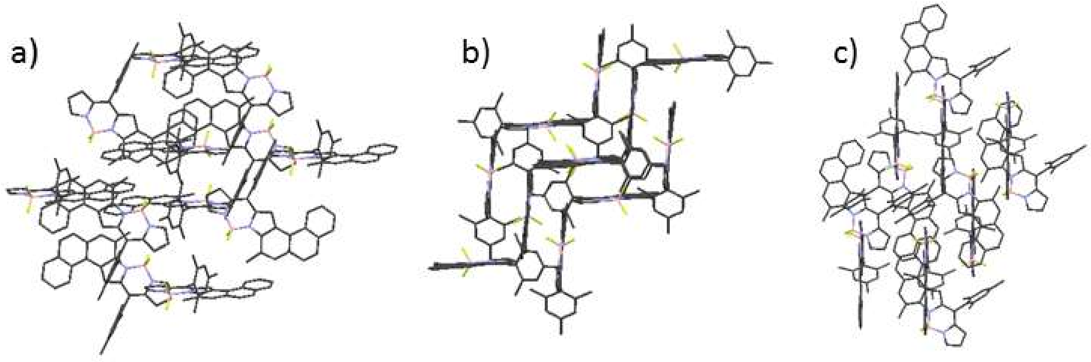

Figure S3.6. Crystal packing of **2f** (a: front view; b: side view; c: top view)

<u>Crystallographic details of BODIPYs **1a**, **1b**, **1c**, **1d**, **1e** and **1g**.</u>

Table S3.1. Selected crystallographic data for **1a**. CCDC number= 1985328

**Chemical formula**	$C_{36}H_{29}BF_2N_2O_{0.50}$	
**Formula weight**	546.42 g/mol	
**Temperature**	250(2) K	
**Wavelength**	0.71073 Å	
**Crystal size**	0.052 x 0.059 x 0.294 mm	
**Crystal habit**	intense purple needle	
**Crystal system**	monoclinic	
**Space group**	C 1 2/c 1	
**Unit cell dimensions**	a = 24.3250(9) Å	α = 90°
	b = 9.9336(4) Å	β = 104.9532(14)°
	c = 28.2901(12) Å	γ = 90°
**Volume**	6604.4(5) $Å^3$	
**Z**	8	
**Density (calculated)**	1.099 g/cm^3	
**Absorption coefficient**	0.072 mm^{-1}	
**F(000)**	2288	
**Theta range for data collection**	1.49 to 25.35°	
**Index ranges**	-29<=h<=29, -11<=k<=11, -33<=l<=34	
**Reflections collected**	56003	
**Independent reflections**	6031 [R(int) = 0.0747]	
**Coverage of independent reflections**	100.0%	
**Absorption correction**	multi-scan	
**Structure solution technique**	direct methods	
**Structure solution program**	SHELXS-97 (Sheldrick 2008)	
**Refinement method**	Full-matrix least-squares on F2	
**Refinement program**	SHELXL-2014/7 (Sheldrick, 2014)	
**Function minimized**	Σ w(Fo2 - Fc2)2	
**Data / restraints / parameters**	6031 / 0 / 387	
**Goodness-of-fit on F2**	1.051	
**Final R indices**	3439 data; I>2σ(I)	R1 = 0.0746, wR2 = 0.2262

	all data	R1 = 0.1390, wR2 = 0.2774
**Weighting scheme**	$w=1/[\sigma^2(F_o^2)+(0.1767P)^2]$<br>where $P=(F_o^2+2F_c^2)/3$	
**Largest diff. peak and hole**	1.066 and -0.424 eÅ^{-3}	
**R.M.S. deviation from mean**	0.190 eÅ^{-}	

Table S3.2. Selected crystallographic data for **1b**. CCDC number= 1985329

**Chemical formula**	$C_{34}H_{25}BF_2N_2$	
**Formula weight**	510.37 g/mol	
**Temperature**	250(2) K	
**Wavelength**	0.71073 Å	
**Crystal size**	0.019 x 0.101 x 0.539 mm	
**Crystal habit**	clear intense purple ribbon	
**Crystal system**	monoclinic	
**Space group**	P 1 21/c 1	
**Unit cell dimensions**	a = 11.6062(5) Å	α = 90°
	b = 32.6033(10) Å	β = 108.632(2)°
	c = 7.6374(3) Å	γ = 90°
**Volume**	2738.53(18) Å^3	
**Z**	4	
**Density (calculated)**	1.238 g/cm^3	
**Absorption coefficient**	0.081 mm^{-1}	
**F(000)**	1064	
**Theta range for data collection**	1.85 to 25.35°	
**Index ranges**	-13<=h<=13, -39<=k<=39, -9<=l<=9	
**Reflections collected**	27427	
**Independent reflections**	5008 [R(int) = 0.0473]	
**Coverage of independent reflections**	99.9%	
**Absorption correction**	none	
**Max. and min. transmission**	0.9980 and 0.9580	
**Structure solution technique**	direct methods	
**Structure solution program**	SHELXS-97 (Sheldrick 2008)	
**Refinement method**	Full-matrix least-squares on F2	
**Refinement program**	SHELXL-2014/7 (Sheldrick, 2014)	
**Function minimized**	Σ w(Fo2 - Fc2)2	
**Data / restraints / parameters**	5008 / 0 / 355	
**Goodness-of-fit on F2**	1.099	
**Final R indices**	3284 data; I>2σ(I)	R1 = 0.0497, wR2 = 0.1316
	all data	R1 = 0.0910, wR2 = 0.1607
**Weighting scheme**	$w=1/[\sigma^2(F_o^2)+(0.0859P)^2+0.2109P]$<br>where $P=(F_o^2+2F_c^2)/3$	
**Largest diff. peak and hole**	0.257 and -0.304 eÅ^{-3}	
**R.M.S. deviation from mean**	0.088 eÅ^{-3}	

Table S3.3. Selected crystallographic data for **1c**. CCDC number= 1985330

**Chemical formula**	$C_{46}H_{33}BF_2N_2$
**Formula weight**	662.55 g/mol
**Temperature**	250(2) K
**Wavelength**	0.71073 Å
**Crystal size**	0.015 x 0.105 x 0.275 mm

**Crystal habit**	intense purple ribbon	
**Crystal system**	orthorhombic	
**Space group**	P n m a	
**Unit cell dimensions**	a = 21.0905(5) Å	α = 90°
	b = 18.9830(6) Å	β = 90°
	c = 8.7486(3) Å	γ = 90°
**Volume**	3502.60(18) $Å^3$	
**Z**	4	
**Density (calculated)**	1.256 g/cm^3	
**Absorption coefficient**	0.080 mm^{-1}	
**F(000)**	1384	
**Theta range for data collection**	1.93 to 25.35°	
**Index ranges**	-25<=h<=24, -22<=k<=22, -10<=l<=10	
**Reflections collected**	81484	
**Independent reflections**	3305 [R(int) = 0.0746]	
**Coverage of independent reflections**	99.7%	
**Absorption correction**	multi-scan	
**Max. and min. transmission**	0.9990 and 0.9780	
**Structure solution technique**	direct methods	
**Structure solution program**	SHELXS-97 (Sheldrick 2008)	
**Refinement method**	Full-matrix least-squares on F^2	
**Refinement program**	SHELXL-2014/7 (Sheldrick, 2014)	
**Function minimized**	Σ w(F_o^2 - F_c^2)^2	
**Data / restraints / parameters**	3305 / 0 / 265	
**Goodness-of-fit on F^2**	1.056	
**Final R indices**	2298 data; I>2σ(I)	R1 = 0.0421, wR2 = 0.1233
	all data	R1 = 0.0808, wR2 = 0.1780
**Weighting scheme**	w=1/[$\sigma^2(F_o^2)$+(0.1088P)^2+0.7000P]<br>where P=(F_o^2+2F_c^2)/3	
**Largest diff. peak and hole**	0.327 and -0.237 e$Å^{-3}$	
**R.M.S. deviation from mean**	0.095 e$Å^{-3}$	

Table S3.4. Selected crystallographic data for **1d**. CCDC number= 1985331

**Chemical formula**	$C_{48}H_{37}BF_2N_2O_2$	
**Formula weight**	722.60 g/mol	
**Temperature**	200(2) K	
**Wavelength**	0.71073 Å	
**Crystal size**	0.033 x 0.097 x 0.229 mm	
**Crystal habit**	intense purple prismatic	
**Crystal system**	triclinic	
**Space group**	P -1	
**Unit cell dimensions**	a = 7.4833(5) Å	α = 111.599(3)°
	b = 14.4445(10) Å	β = 95.542(3)°
	c = 19.1563(10) Å	γ = 94.737(3)°
**Volume**	1900.6(2) $Å^3$	
**Z**	2	
**Density (calculated)**	1.263 g/cm^3	
**Absorption coefficient**	0.083 mm^{-1}	
**F(000)**	756	
**Theta range for data collection**	1.15 to 25.35°	
**Index ranges**	-9<=h<=9, -17<=k<=17, -23<=l<=23	
**Reflections collected**	31803	

**Independent reflections**	6928 [R(int) = 0.0644]
**Coverage of independent reflections**	99.9%
**Absorption correction**	multi-scan
**Max. and min. transmission**	0.9970 and 0.9810
**Structure solution technique**	direct methods
**Structure solution program**	SHELXS-97 (Sheldrick 2008)
**Refinement method**	Full-matrix least-squares on F^2
**Refinement program**	SHELXL-2014 (Sheldrick 2014)
**Function minimized**	Σ w(F_o^2 - F_c^2)^2
**Data / restraints / parameters**	6928 / 12 / 589
**Goodness-of-fit on F^2**	1.064
**Final R indices**	4056 data; I>2σ(I) R1 = 0.0528, wR2 = 0.1404
	all data R1 = 0.1122, wR2 = 0.1859
**Weighting scheme**	w=1/[σ^2(F_o^2)+(0.1022P)^2+0.0087P]<br>where P=(F_o^2+2F_c^2)/3
**Largest diff. peak and hole**	0.322 and -0.351 eÅ^{-3}
**R.M.S. deviation from mean**	0.069 eÅ^{-3}

Table S3.5. Selected crystallographic data for **1f**. CCDC number= 1985332

**Chemical formula**	**$C_{31}H_{25}BF_2N_2$**	
**Formula weight**	474.34 g/mol	
**Temperature**	296(2) K	
**Wavelength**	0.71073 Å	
**Crystal size**	0.054 x 0.295 x 0.366 mm	
**Crystal habit**	dark purple plate	
**Crystal system**	triclinic	
**Space group**	P -1	
**Unit cell dimensions**	a = 7.9160(14) Å	α = 79.032(11)°
	b = 10.089(2) Å	β = 88.721(10)°
	c = 15.968(3) Å	γ = 85.253(10)°
**Volume**	1247.7(4) Å^3	
**Z**	2	
**Density (calculated)**	1.263 g/cm^3	
**Absorption coefficient**	0.084 mm^{-1}	
**F(000)**	496	
**Theta range for data collection**	1.30 to 25.35°	
**Index ranges**	-9<=h<=9, -12<=k<=12, -19<=l<=19	
**Reflections collected**	62317	
**Independent reflections**	4551 [R(int) = 0.0331]	
**Coverage of independent reflections**	99.7%	
**Absorption correction**	multi-scan	
**Max. and min. transmission**	0.9960 and 0.9700	
**Refinement method**	Full-matrix least-squares on F^2	
**Refinement program**	SHELXL-2014/7 (Sheldrick, 2014)	
**Function minimized**	Σ w(F_o^2 - F_c^2)^2	
**Data / restraints / parameters**	4551 / 0 / 329	
**Goodness-of-fit on F^2**	1.031	
**Final R indices**	3806 data; I>2σ(I)	R1 = 0.0440, wR2 = 0.1348
	all data	R1 = 0.0602, wR2 = 0.1632
**Weighting scheme**	w=1/[σ^2(F_o^2)+(0.1024P)^2+0.3569P]	

	where $P=(F_o^2+2F_c^2)/3$
**Largest diff. peak and hole**	0.712 and -0.396 eÅ^{-3}
**R.M.S. deviation from mean**	0.147 eÅ^{-3}

Table S3.6. Selected crystallographic data for **1g**. CCDC number= 1985333

**Chemical formula**	**$C_{36}H_{29}BF_2N_2$**	
**Formula weight**	538.42 g/mol	
**Temperature**	296(2) K	
**Wavelength**	0.71073 Å	
**Crystal size**	0.071 x 0.149 x 0.215 mm	
**Crystal habit**	purple prismatic	
**Crystal system**	triclinic	
**Space group**	P -1	
**Unit cell dimensions**	a = 10.1937(6) Å	α = 105.446(3)°
	b = 11.0010(6) Å	β = 103.611(3)°
	c = 14.2800(7) Å	γ = 94.765(3)°
**Volume**	1481.92(14) Å^3	
**Z**	2	
**Density (calculated)**	1.207 g/cm^3	
**Absorption coefficient**	0.078 mm^{-1}	
**F(000)**	564	
**Theta range for data collection**	1.54 to 25.34°	
**Index ranges**	-12<=h<=12, -13<=k<=13, -17<=l<=17	
**Reflections collected**	21050	
**Independent reflections**	5433 [R(int) = 0.0840]	
**Coverage of independent reflections**	100.0%	
**Absorption correction**	multi-scan	
**Max. and min. transmission**	0.9940 and 0.9830	
**Refinement method**	Full-matrix least-squares on F^2	
**Refinement program**	SHELXL-2014/7 (Sheldrick, 2014)	
**Function minimized**	Σ w$(F_o^2 - F_c^2)^2$	
**Data / restraints / parameters**	5433 / 0 / 375	
**Goodness-of-fit on F^2**	1.031	
**Final R indices**	2660 data; I>2σ(I)	R1 = 0.0561, wR2 = 0.1473
	all data	R1 = 0.1582, wR2 = 0.2279
**Weighting scheme**	$w=1/[\sigma^2(F_o^2)+(0.1180P)^2]$ where $P=(F_o^2+2F_c^2)/3$	
**Largest diff. peak and hole**	0.384 and -0.487 eÅ^{-3}	
**R.M.S. deviation from mean**	0.145 eÅ^{-3}	

Crystallographic details of BODIPYs **2a, 2e**, **2f** ,**2g** and dipyrromethene **2d**.

Table S3.7. Selected crystallographic data for **2a**. CCDC number= 1985334

**Chemical formula**	**$C_{36}H_{29}BF_2N_2$**
**Formula weight**	538.42 g/mol
**Temperature**	250(2) K
**Wavelength**	0.71073 Å
**Crystal size**	0.137 x 0.144 x 0.980 mm
**Crystal habit**	dark blue prismatic
**Crystal system**	monoclinic

**Space group**	P 1 21/n 1	
**Unit cell dimensions**	a = 14.395(4) Å	α = 90°
	b = 14.038(3) Å	β = 108.639(11)°
	c = 18.526(5) Å	γ = 90°
**Volume**	3547.3(15) $Å^3$	
**Z**	4	
**Density (calculated)**	1.008 g/cm^3	
**Absorption coefficient**	0.066 mm^{-1}	
**F(000)**	1128	
**Theta range for data collection**	2.14 to 25.35°	
**Index ranges**	-17<=h<=17, -16<=k<=16, -22<=l<=22	
**Reflections collected**	93294	
**Independent reflections**	6466 [R(int) = 0.0928]	
**Coverage of independent reflections**	99.6%	
**Absorption correction**	multi-scan	
**Max. and min. transmission**	0.9910 and 0.9390	
**Refinement method**	Full-matrix least-squares on F^2	
**Refinement program**	SHELXL-2014/7 (Sheldrick, 2014)	
**Function minimized**	$\Sigma w(F_o^2 - F_c^2)^2$	
**Data / restraints / parameters**	6466 / 0 / 375	
**Goodness-of-fit on F^2**	1.030	
**Final R indices**	3298 data; I>2σ(I)	R1 = 0.0962, wR2 = 0.2819
	all data	R1 = 0.1716, wR2 = 0.3532
**Weighting scheme**	$w=1/[\sigma^2(F_o^2)+(0.2000P)^2+1.9700P]$ where $P=(F_o^2+2F_c^2)/3$	
**Largest diff. peak and hole**	0.604 and -0.307 $eÅ^{-3}$	
**R.M.S. deviation from mean**	0.094 $eÅ^{-3}$	

Table S3.8. Selected crystallographic data for **2d´**. CCDC number= 1985335

**Chemical formula**	$\mathbf{C_{48}H_{38}N_2O_2}$	
**Formula weight**	674.80 g/mol	
**Temperature**	200(2) K	
**Wavelength**	0.71073 Å	
**Crystal size**	0.028 x 0.045 x 0.205 mm	
**Crystal habit**	dark purple prismatic	
**Crystal system**	monoclinic	
**Space group**	C 1 2/c 1	
**Unit cell dimensions**	a = 20.361(6) Å	α = 90°
	b = 16.095(5) Å	β = 114.048(7)°
	c = 23.355(7) Å	γ = 90°
**Volume**	6989.(4) $Å^3$	
**Z**	8	
**Density (calculated)**	1.283 g/cm^3	
**Absorption coefficient**	0.078 mm^{-1}	
**F(000)**	2848	
**Theta range for data collection**	1.67 to 25.49°	
**Index ranges**	-24<=h<=24, -19<=k<=19, -27<=l<=28	
**Reflections collected**	41414	
**Independent reflections**	6439 [R(int) = 0.3225]	
**Coverage of independent**	98.7%	

**reflections**	
**Absorption correction**	multi-scan
**Structure solution technique**	direct methods
**Structure solution program**	SHELXS-97 (Sheldrick 2008)
**Refinement method**	Full-matrix least-squares on F^2
**Refinement program**	SHELXL-2014/7 (Sheldrick, 2014)
**Function minimized**	Σ w(F_o^2 - F_c^2)^2
**Data / restraints / parameters**	6439 / 0 / 474
**Goodness-of-fit on F^2**	1.000
**Final R indices**	2044 data; I>2σ(I) R1 = 0.0794, wR2 = 0.1612
	all data R1 = 0.2983, wR2 = 0.2712
**Weighting scheme**	w=1/[σ^2(F_o^2)+(0.1010P)^2]<br>where P=(F_o^2+2F_c^2)/3
**Largest diff. peak and hole**	0.386 and -0.406 eÅ^{-3}
**R.M.S. deviation from mean**	0.104 eÅ^{-3}

Table S3.9. Selected crystallographic data for **2e**. CCDC number= 1985336

**Chemical formula**	**$C_{44}H_{33}BF_2N_2$**	
**Formula weight**	638.53 g/mol	
**Temperature**	250(2) K	
**Wavelength**	0.71073 Å	
**Crystal size**	0.100 x 0.116 x 0.195 mm	
**Crystal habit**	dark blue-purple prismatic	
**Crystal system**	monoclinic	
**Space group**	P 1 21/c 1	
**Unit cell dimensions**	a = 12.5426(5) Å	α = 90°
	b = 41.2031(15) Å	β = 115.8809(10)°
	c = 13.9741(5) Å	γ = 90°
**Volume**	6497.4(4) Å^3	
**Z**	8	
**Density (calculated)**	1.306 g/cm^3	
**Absorption coefficient**	0.083 mm^{-1}	
**F(000)**	2672	
**Theta range for data collection**	0.99 to 25.35°	
**Index ranges**	-14<=h<=15, -49<=k<=49, -16<=l<=16	
**Reflections collected**	131931	
**Independent reflections**	11877 [R(int) = 0.0523]	
**Coverage of independent reflections**	99.9%	
**Absorption correction**	multi-scan	
**Max. and min. transmission**	0.9920 and 0.9840	
**Refinement method**	Full-matrix least-squares on F^2	
**Refinement program**	SHELXL-2014/7 (Sheldrick, 2014)	
**Function minimized**	Σ w(F_o^2 - F_c^2)^2	
**Data / restraints / parameters**	11877 / 0 / 893	
**Goodness-of-fit on F^2**	1.039	
**Δ/σ_{max}**	0.001	
**Final R indices**	7903 data; I>2σ(I)	R1 = 0.0466, wR2 = 0.1442
	all data	R1 = 0.0862, wR2 = 0.1878
**Weighting scheme**	w=1/[σ^2(F_o^2)+(0.1251P)^2+0.0648P]<br>where P=(F_o^2+2F_c^2)/3	
**Largest diff. peak and hole**	0.385 and -0.322 eÅ^{-3}	

**R.M.S. deviation from mean**	0.115 eÅ^{-3}

Table S3.10. Selected crystallographic data for **2f**. CCDC number= 1985337

**Chemical formula**	**$C_{31}H_{25}BF_2N_2$**	
**Formula weight**	474.34 g/mol	
**Temperature**	296(2) K	
**Wavelength**	0.71073 Å	
**Crystal size**	0.054 x 0.295 x 0.366 mm	
**Crystal habit**	dark purple plate	
**Crystal system**	triclinic	
**Space group**	P -1	
**Unit cell dimensions**	a = 7.9160(14) Å	α = 79.032(11)°
	b = 10.089(2) Å	β = 88.721(10)°
	c = 15.968(3) Å	γ = 85.253(10)°
**Volume**	1247.7(4) Å^3	
**Z**	2	
**Density (calculated)**	1.263 g/cm^3	
**Absorption coefficient**	0.084 mm^{-1}	
**F(000)**	496	
**Theta range for data collection**	1.30 to 25.35°	
**Index ranges**	-9<=h<=9, -12<=k<=12, -19<=l<=19	
**Reflections collected**	62317	
**Independent reflections**	4551 [R(int) = 0.0331]	
**Coverage of independent reflections**	99.7%	
**Absorption correction**	multi-scan	
**Max. and min. transmission**	0.9960 and 0.9700	
**Refinement method**	Full-matrix least-squares on F^2	
**Refinement program**	SHELXL-2014/7 (Sheldrick, 2014)	
**Function minimized**	$\Sigma\ w(F_o^2 - F_c^2)^2$	
**Data / restraints / parameters**	4551 / 0 / 329	
**Goodness-of-fit on F^2**	1.031	
**Final R indices**	3806 data; I>2σ(I)	R1 = 0.0440, wR2 = 0.1348
	all data	R1 = 0.0602, wR2 = 0.1632
**Weighting scheme**	$w=1/[\sigma^2(F_o^2)+(0.1024P)^2+0.3569P]$ where $P=(F_o^2+2F_c^2)/3$	
**Largest diff. peak and hole**	0.712 and -0.396 eÅ^{-3}	
**R.M.S. deviation from mean**	0.147 eÅ^{-3}	

Table S3.11. Selected crystallographic data for **2g**. CCDC number= 1985338

**Chemical formula**	**$C_{36}H_{29}BF_2N_2$**	
**Formula weight**	538.42 g/mol	
**Temperature**	250(2) K	
**Wavelength**	0.71073 Å	
**Crystal size**	0.107 x 0.194 x 0.291 mm	
**Crystal habit**	intense green-purple prismatic	
**Crystal system**	tetragonal	
**Space group**	I 41 c d	
**Unit cell dimensions**	a = 17.6517(4) Å	α = 90°
	b = 17.6517(4) Å	β = 90°
	c = 36.5175(10) Å	γ = 90°

**Volume**	11378.2(6) Å^3
**Z**	16
**Density (calculated)**	1.257 g/cm^3
**Absorption coefficient**	0.082 mm^{-1}
**F(000)**	4512
**Theta range for data collection**	1.98 to 25.35°
**Index ranges**	-21<=h<=21, -21<=k<=21, -43<=l<=43
**Reflections collected**	104985
**Independent reflections**	5216 [R(int) = 0.0518]
**Coverage of independent reflections**	99.9%
**Absorption correction**	multi-scan
**Max. and min. transmission**	0.9910 and 0.9770
**Refinement method**	Full-matrix least-squares on F^2
**Refinement program**	SHELXL-2014/7 (Sheldrick, 2014)
**Function minimized**	$\Sigma w(F_o^2 - F_c^2)^2$
**Data / restraints / parameters**	5216 / 1 / 375
**Goodness-of-fit on F^2**	1.010
**Final R indices**	4230 data; I>2σ(I) R1 = 0.0378, wR2 = 0.0988
	all data R1 = 0.0632, wR2 = 0.1486
**Weighting scheme**	$w=1/[\sigma^2(F_o^2)+(0.0871P)^2+9.7000P]$<br>where $P=(F_o^2+2F_c^2)/3$
**Absolute structure parameter**	0.4(2)
**Largest diff. peak and hole**	0.289 and -0.559 eÅ^{-3}
**R.M.S. deviation from mean**	0.156 eÅ^{-3}

4. UV/Vis Absorption and Emission Spectra

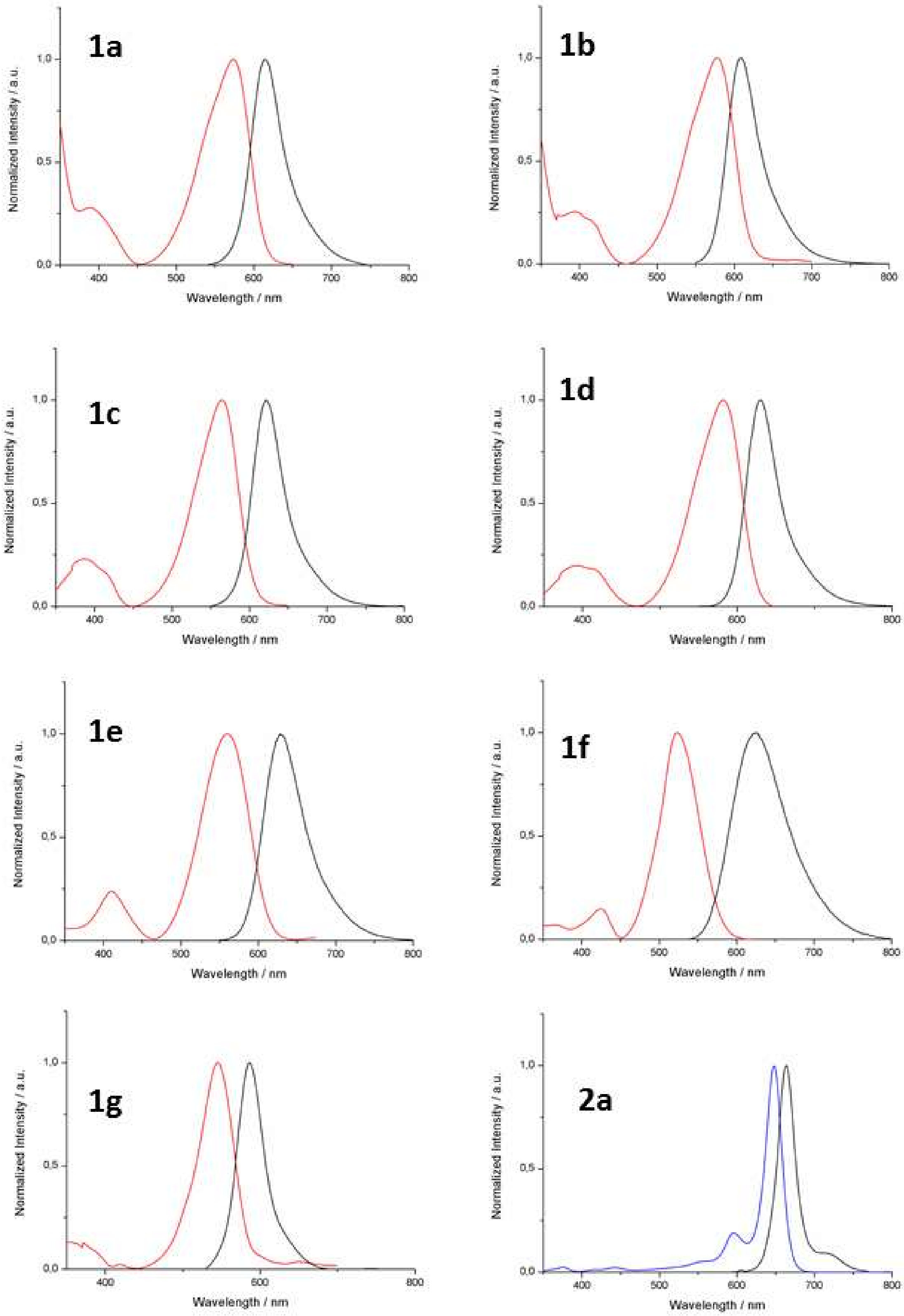

Figure S4.1. UV/Vis Absorption (red/blue) and Emission spectra (black) of **1a-1g** and **2a-2g**

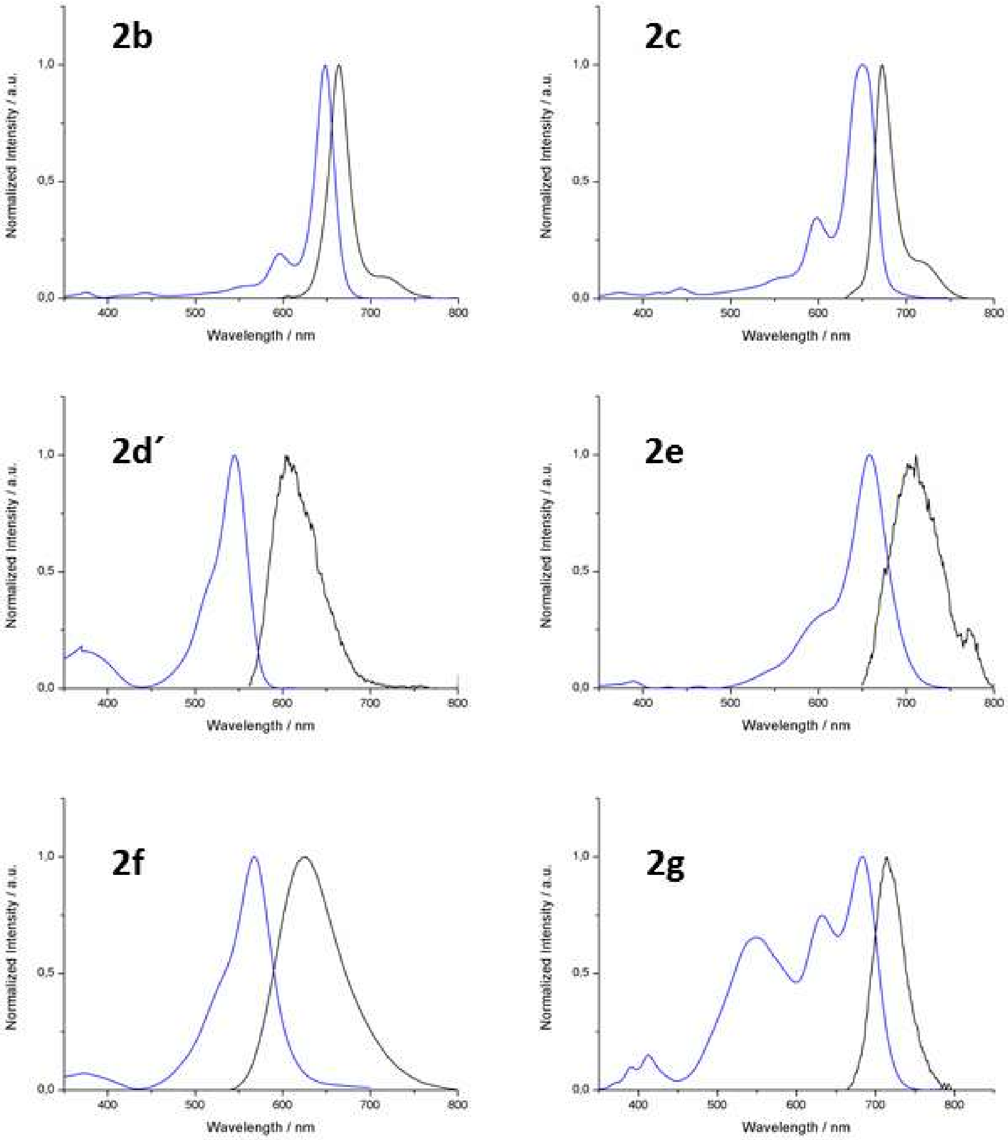

Figure S4.1. Continued

5. NMR spectra

Boronic Acid **e**

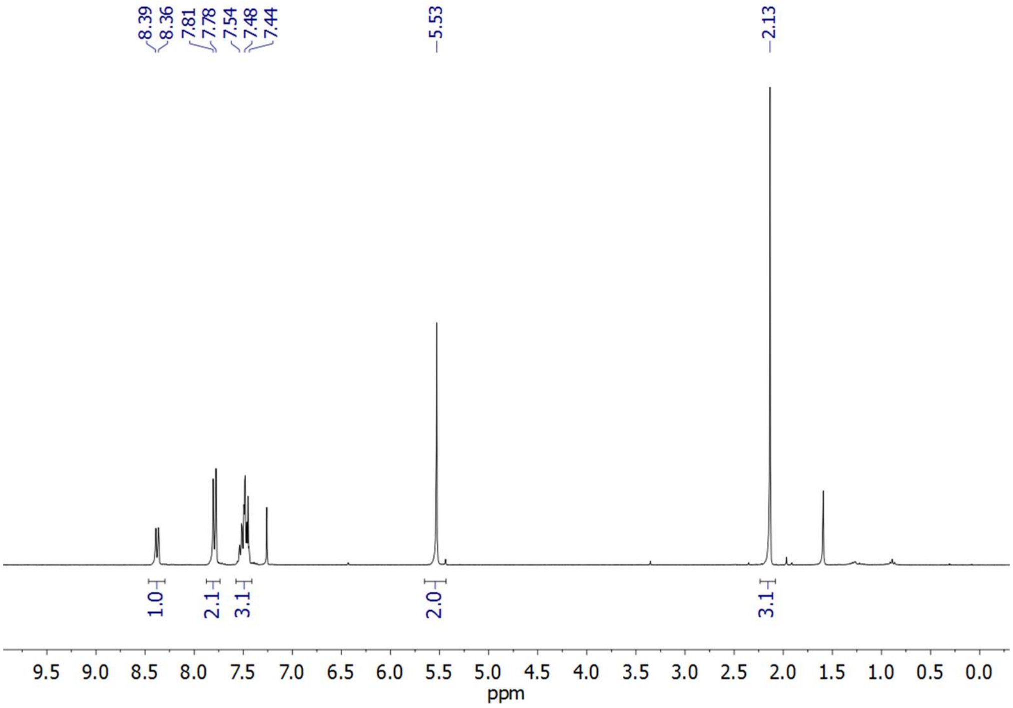

Figure S5.1. ^{1}H-NMR spectrum ($CDCl_3$) of **e**

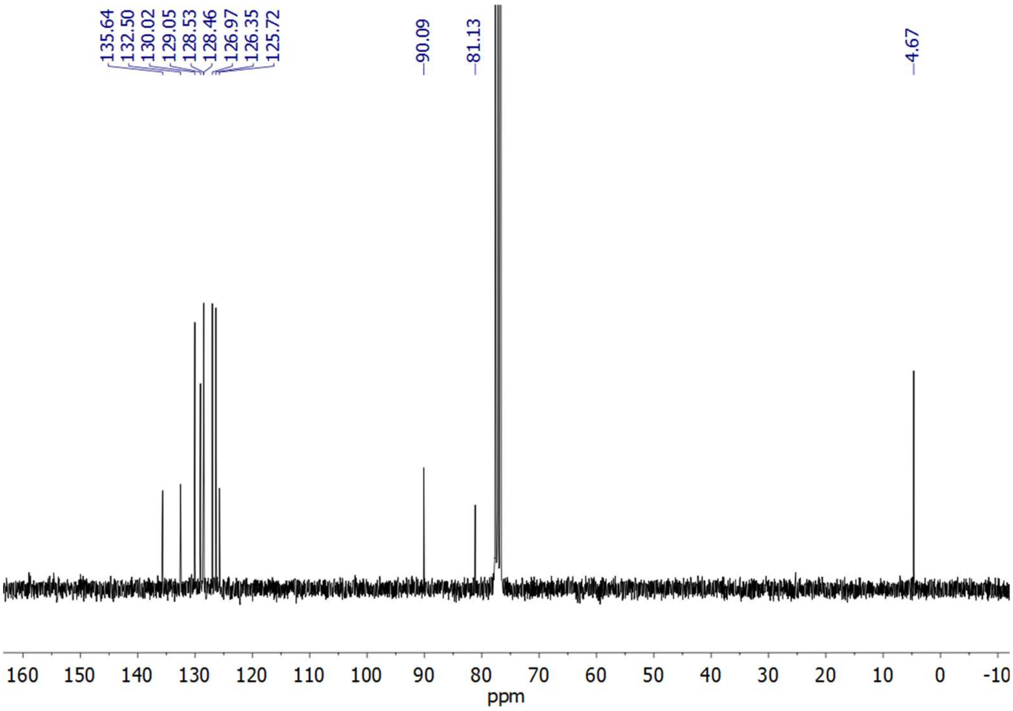

Figure S5.2. ^{13}C-NMR spectrum ($CDCl_3$) of **e**

BODIPY **1a**

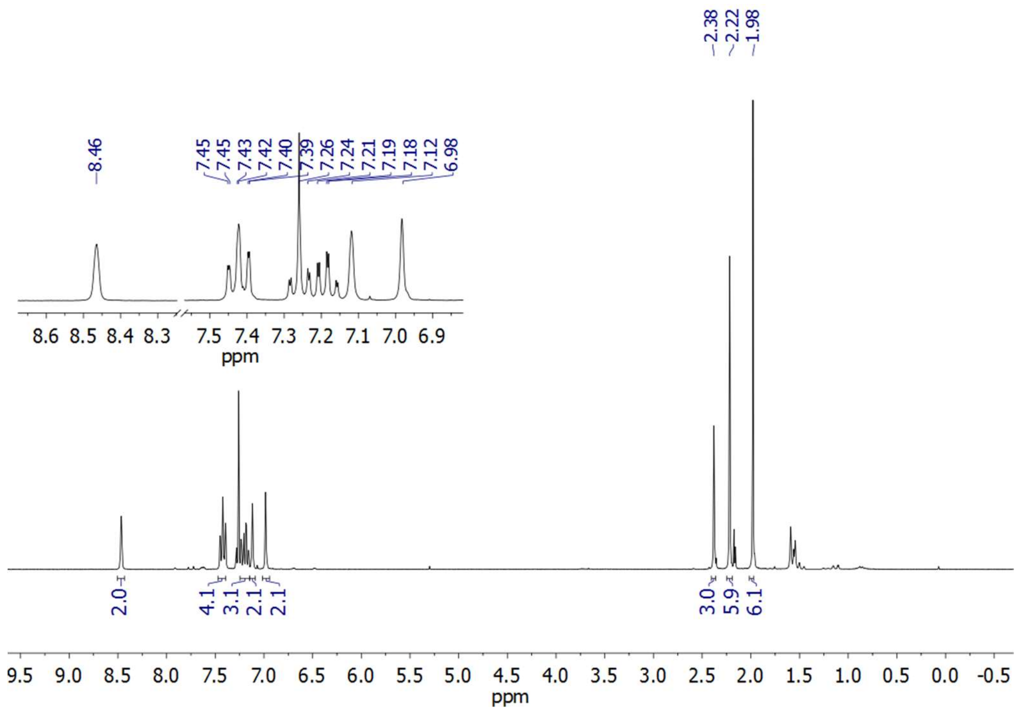

Figure S5.3. ^{1}H-NMR spectrum ($CDCl_3$) of **1a**

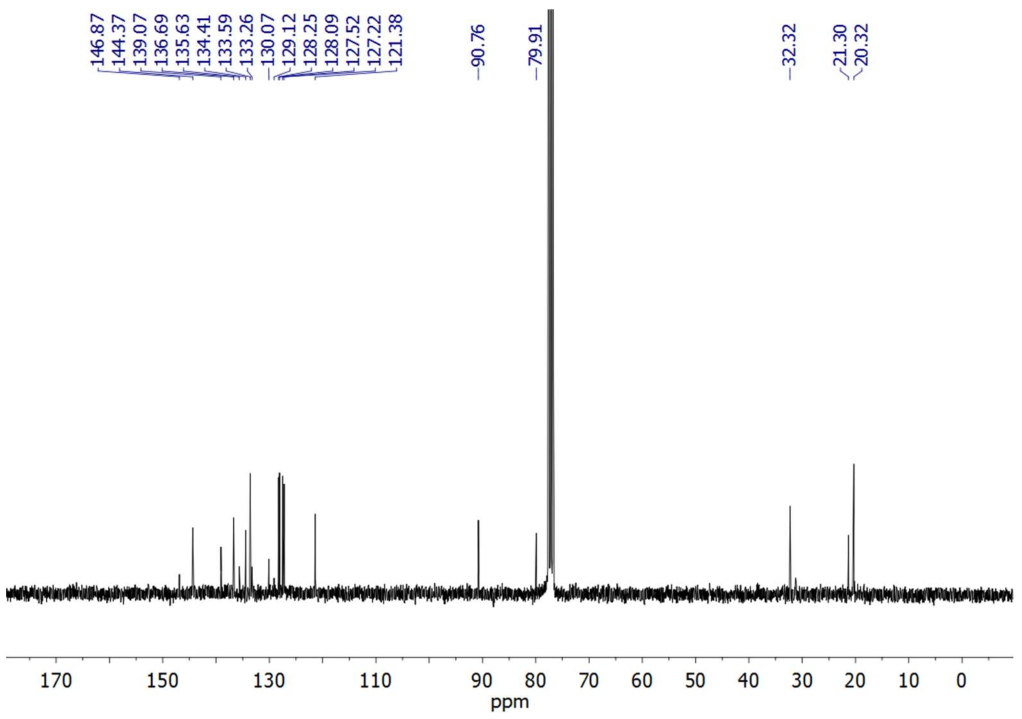

Figure S5.4. ^{13}C-NMR spectrum ($CDCl_3$) of **1a**

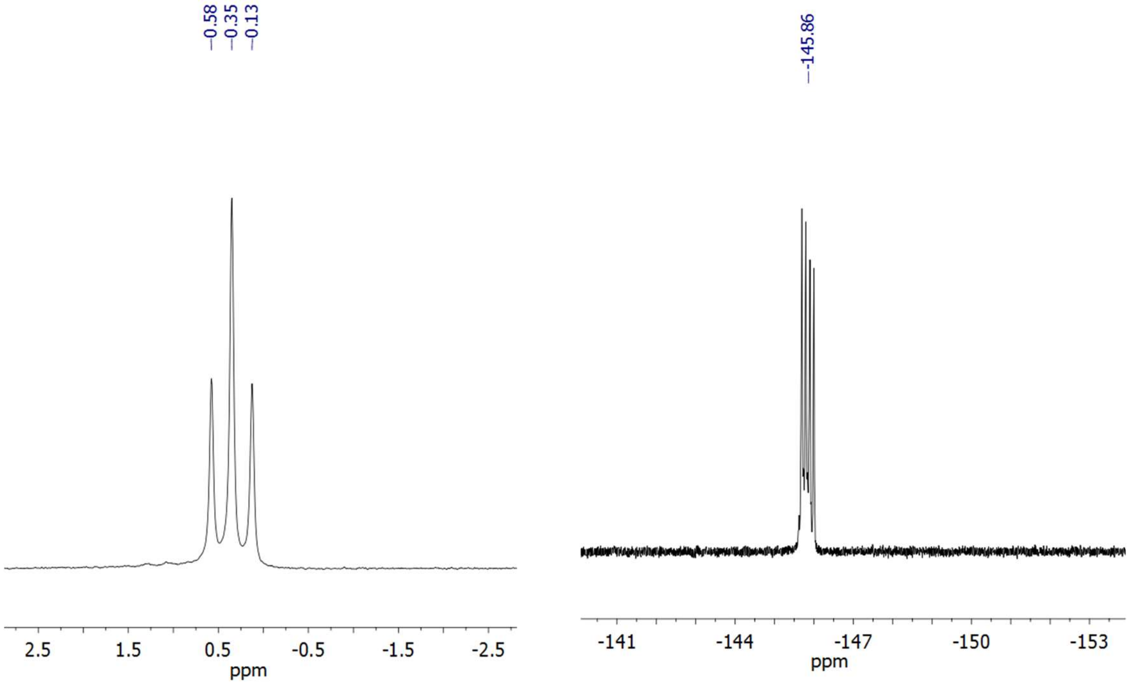

Figure S5.5. ^{11}B-NMR (left) and ^{19}F-NMR (right) spectra ($CDCl_3$) of **1a**

BODIPY **1b**

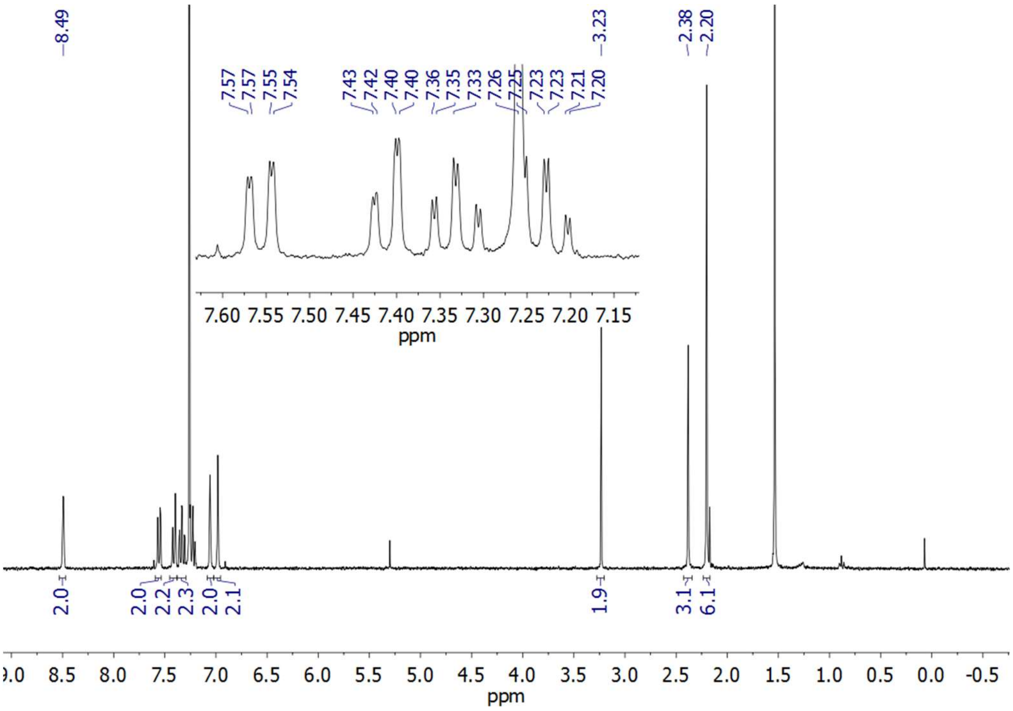

Figure S5.6. ^{1}H-NMR spectrum ($CDCl_3$) of **1b**

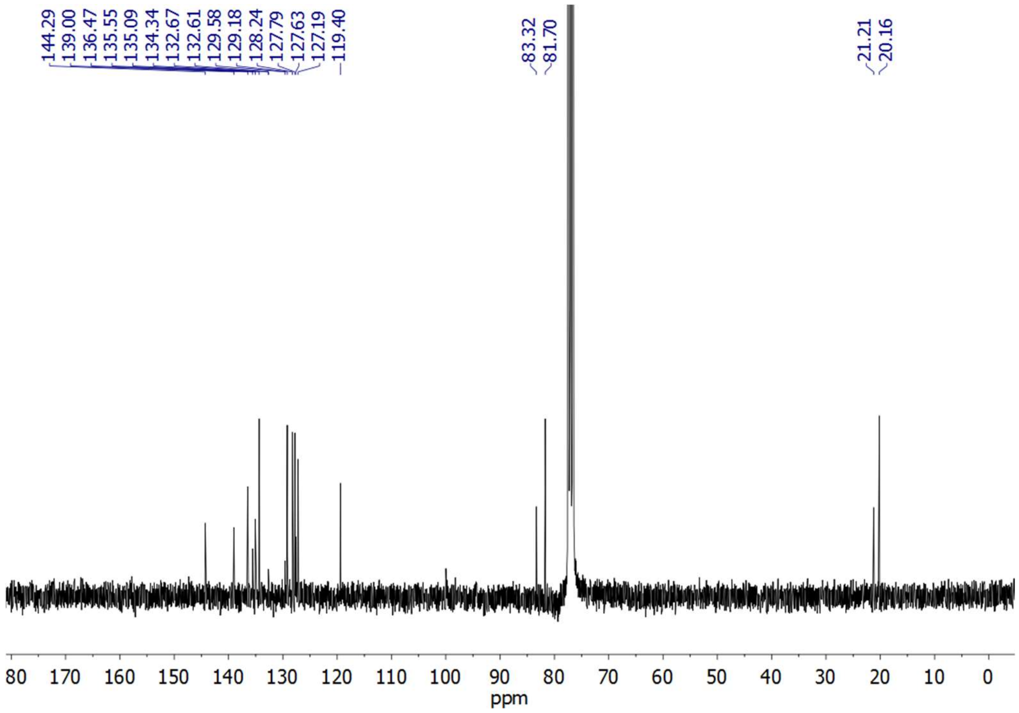

Figure S5.7. ^{13}C-NMR spectrum ($CDCl_3$) of **1b**

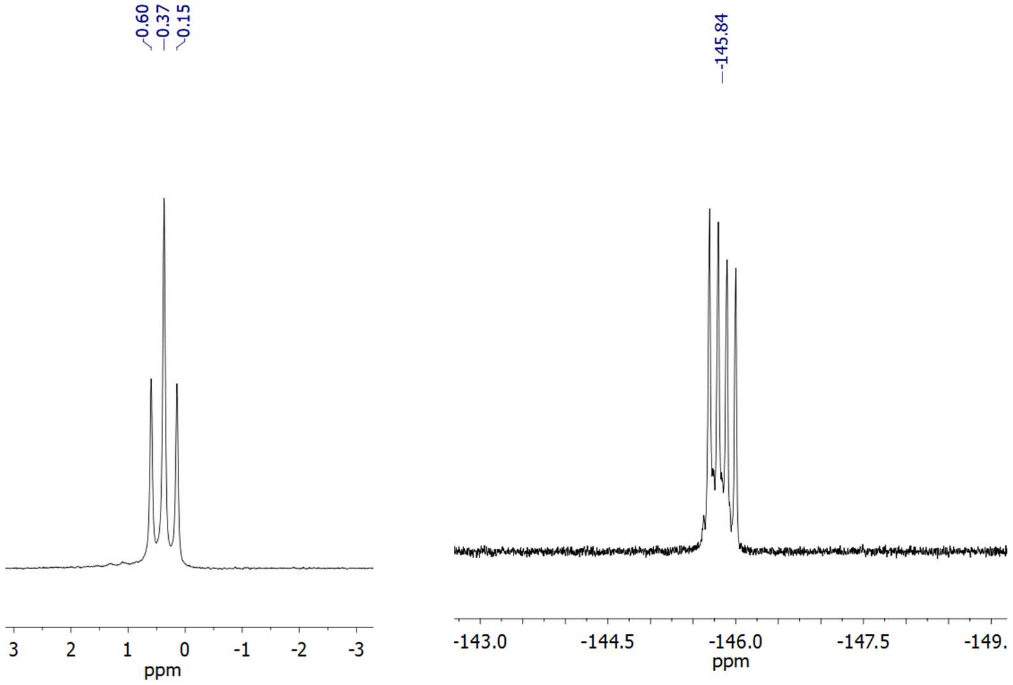

Figure S5.8. ^{11}B-NMR (left) and ^{19}F-NMR (right) spectra ($CDCl_3$) of **1b**

BODIPY **1c**

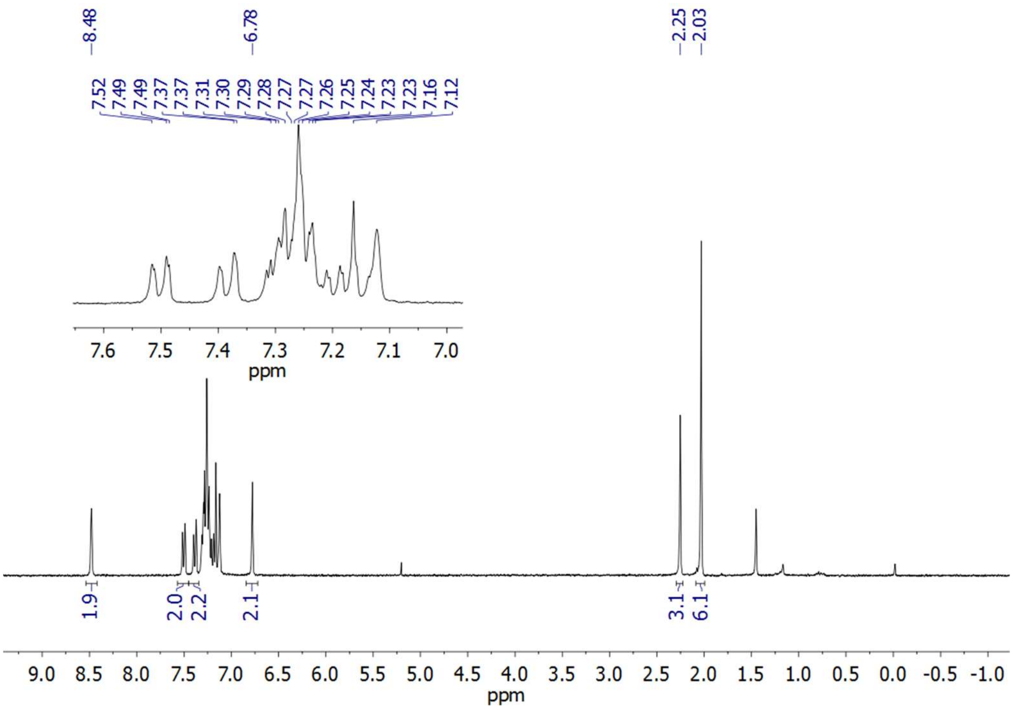

Figure S5.9. ^{1}H-NMR spectrum ($CDCl_3$) of **1c**

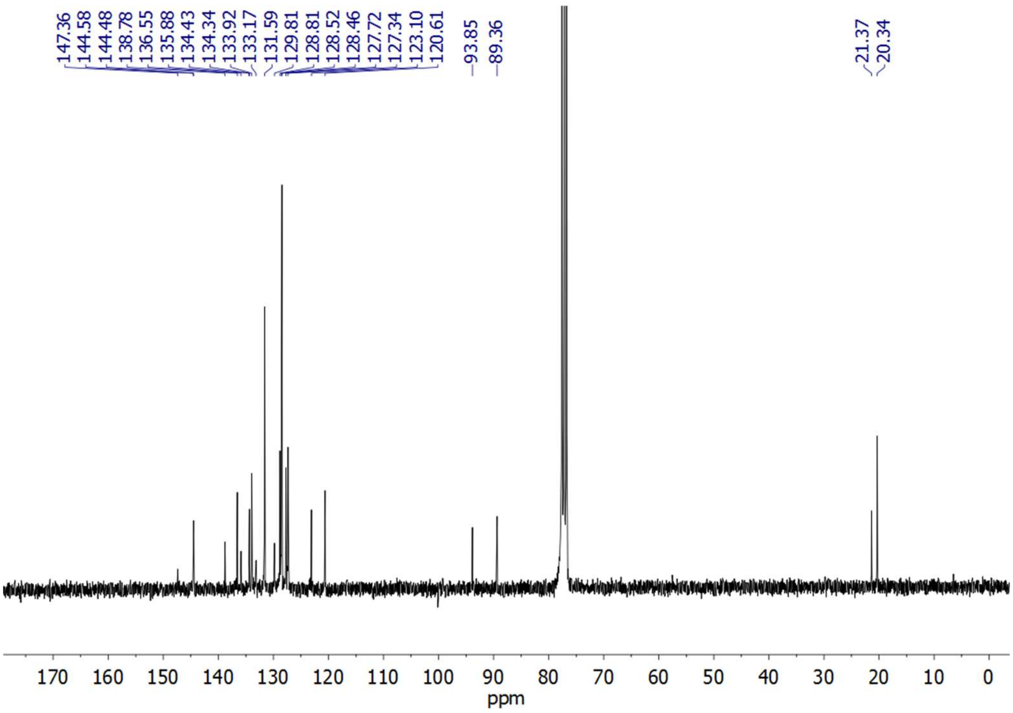

Figure S5.10. ^{13}C-NMR spectrum ($CDCl_3$) of **1c**

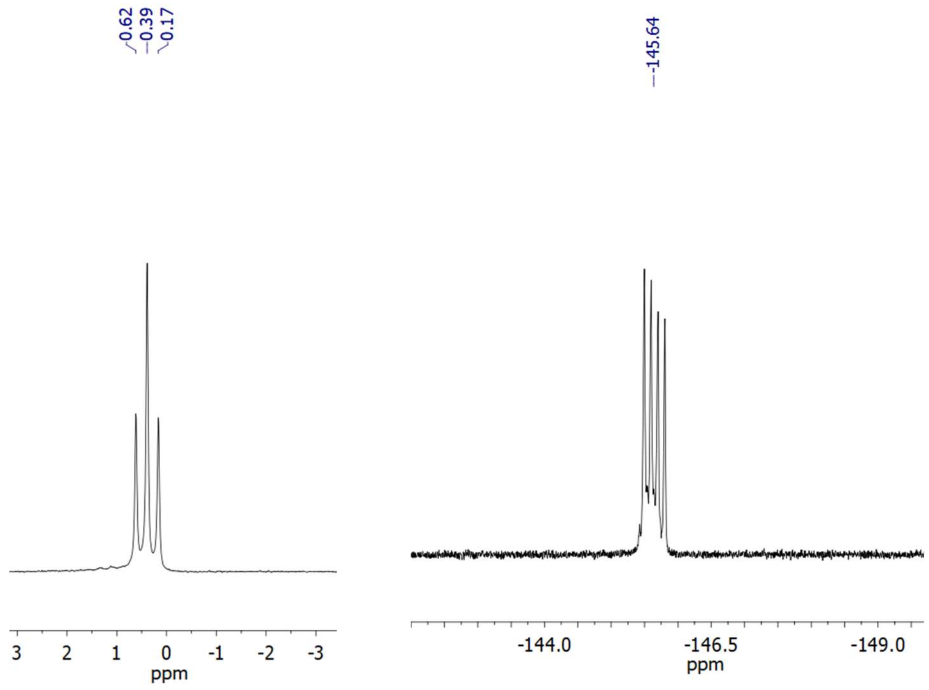

Figure S5.11. ^{11}B-NMR (left) and ^{19}F-NMR (right) spectra ($CDCl_3$) of **1c**

<u>BODIPY **1d**</u>

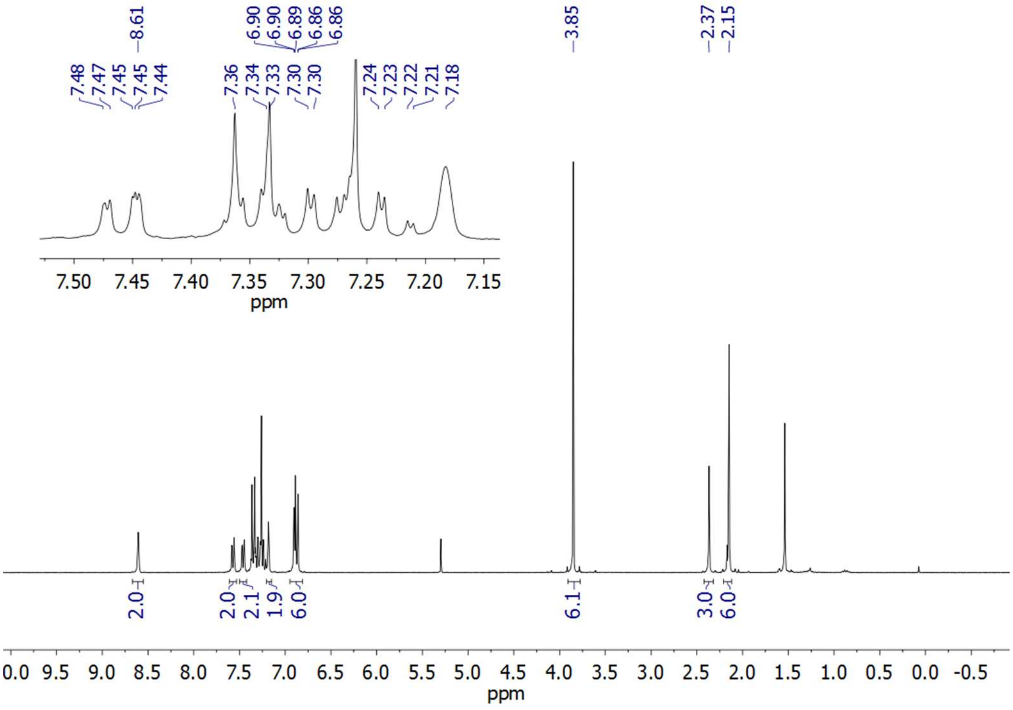

Figure S5.12. ^{1}H-NMR spectrum ($CDCl_3$) of **1d**

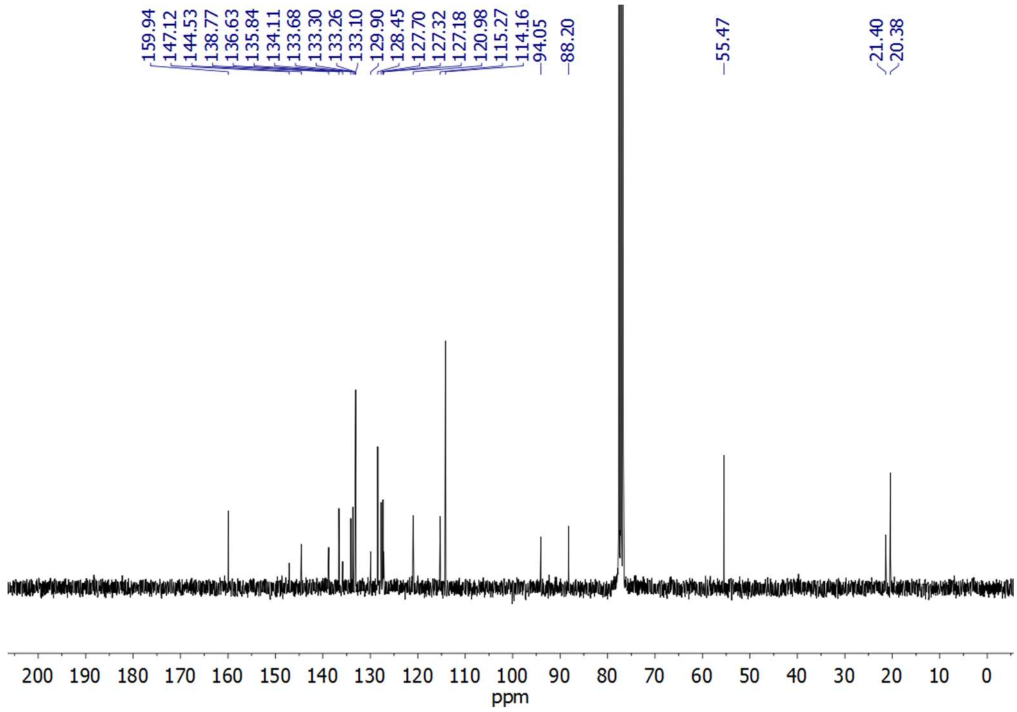

Figure S5.13. ^{13}C-NMR spectrum ($CDCl_3$) of **1d**

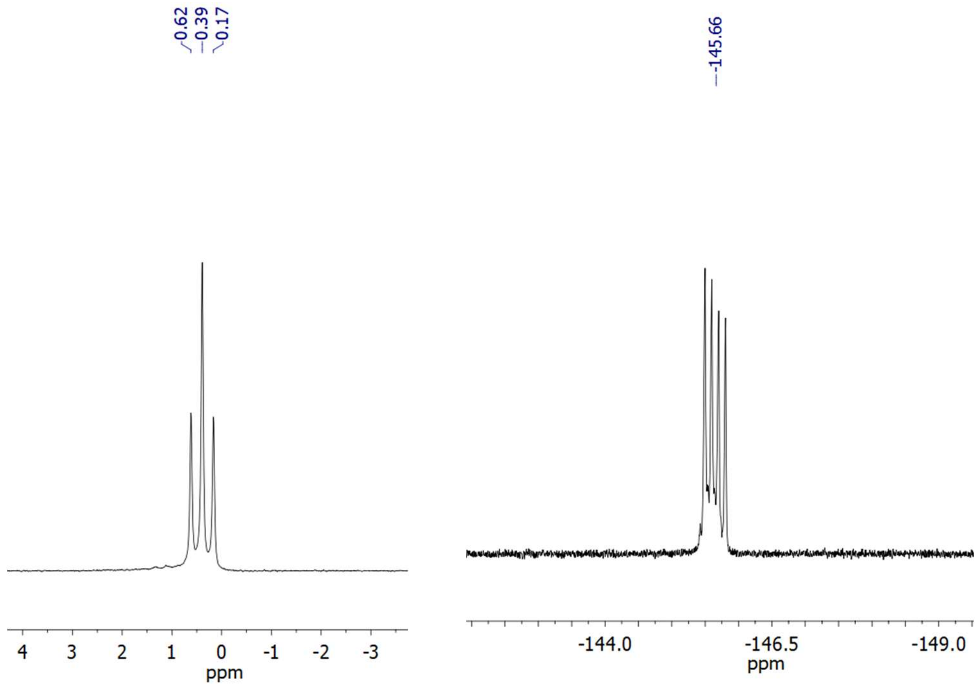

Figure S5.14. ^{11}B-NMR (left) and ^{19}F-NMR (right) spectra ($CDCl_3$) of **1d**

<u>BODIPY **1e**</u>

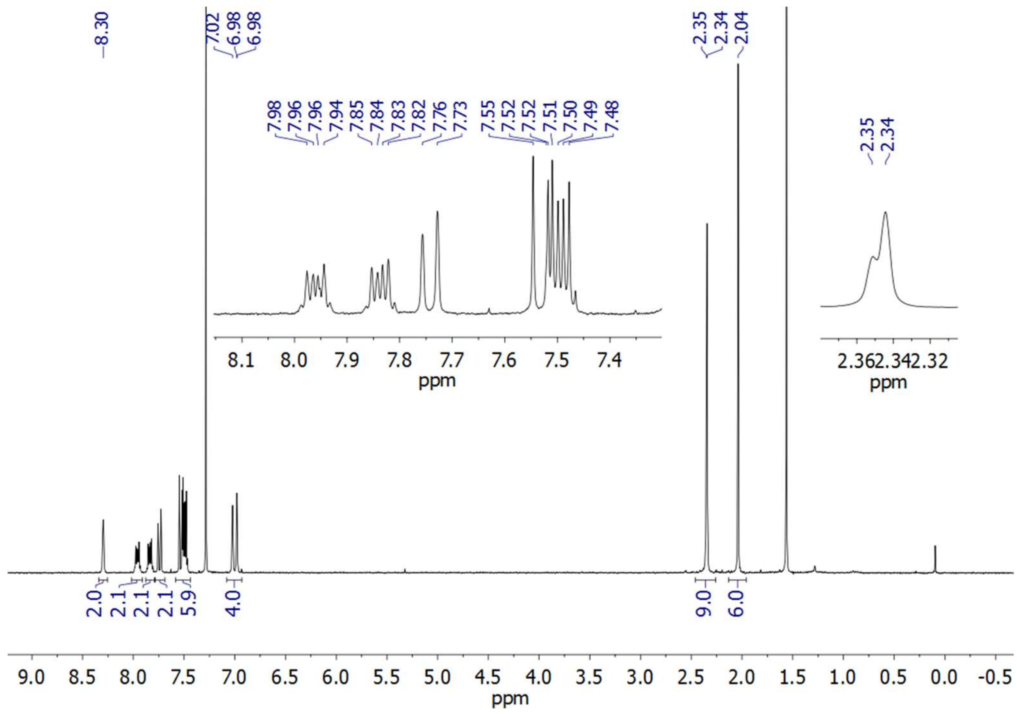

Figure S5.15. ^{1}H-NMR spectrum ($CDCl_3$) of **1e**

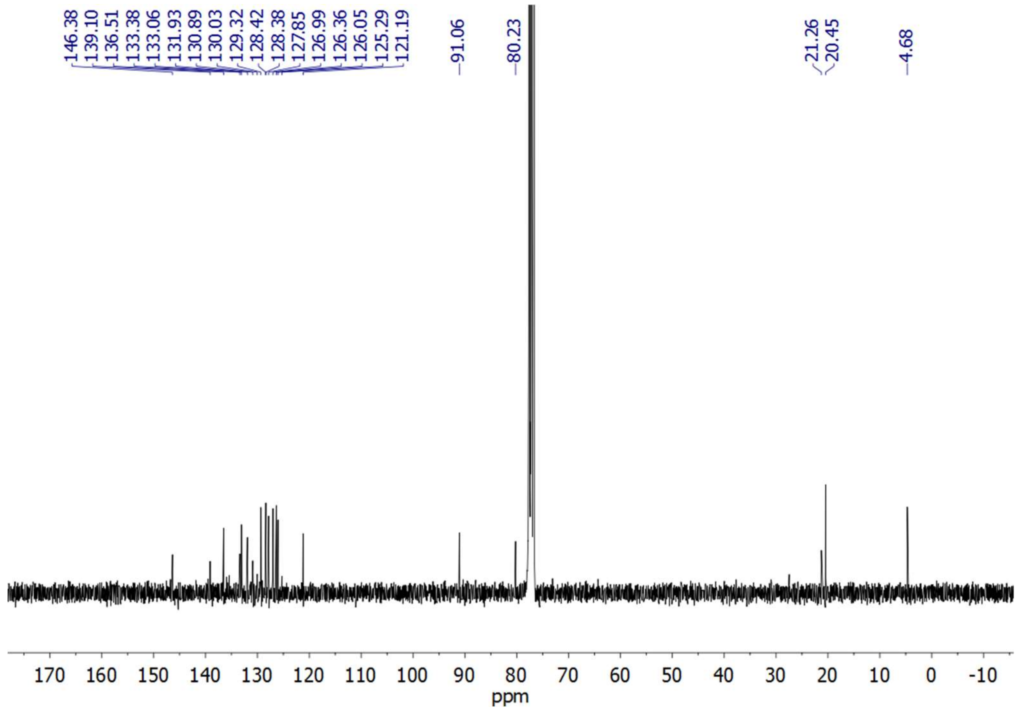

Figure S5.16. ^{13}C-NMR spectrum ($CDCl_3$) of **1e**

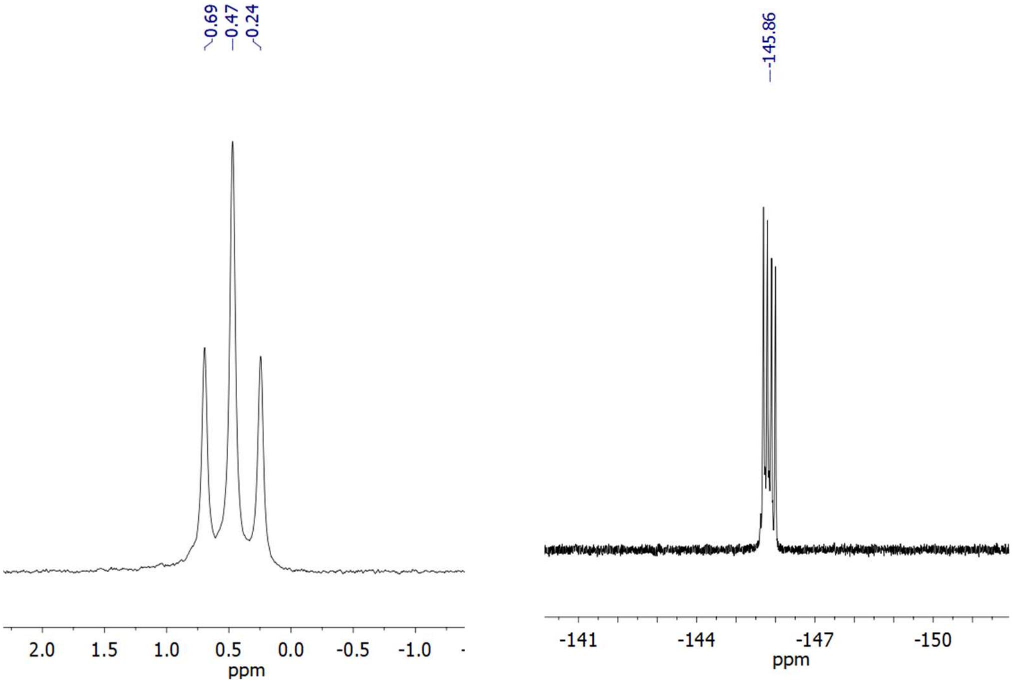

Figure S5.17. ^{11}B-NMR (left) and ^{19}F-NMR (right) spectra ($CDCl_3$) of **1e**

<u>BODIPY **1f**</u>

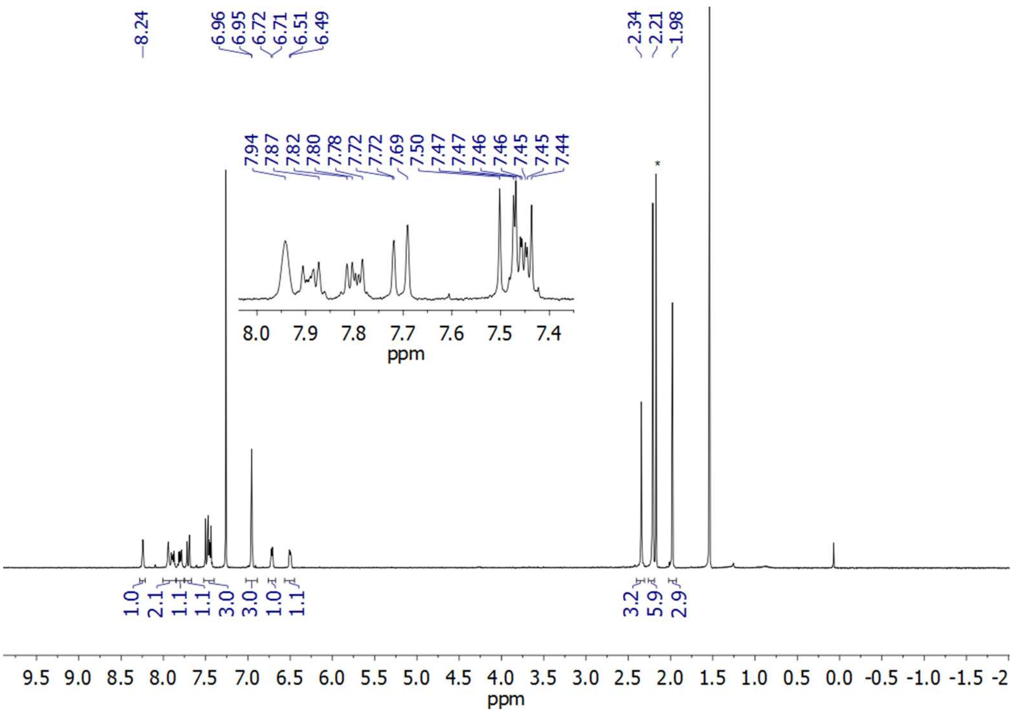

Figure S5.18. ^{1}H-NMR spectrum ($CDCl_3$) of **1f**

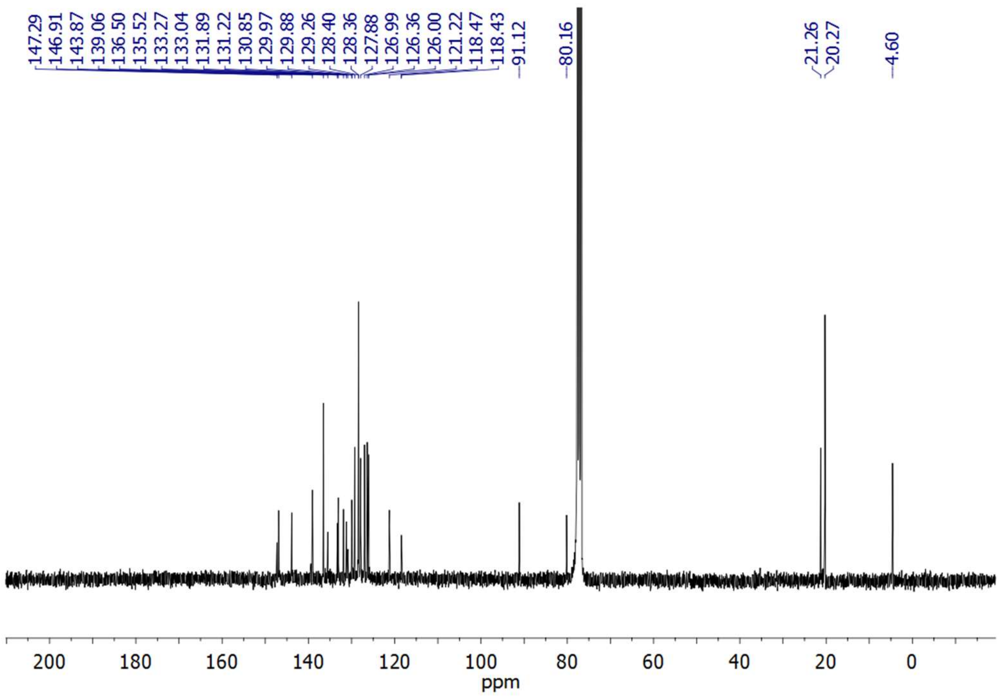

Figure S5.19. ^{13}C-NMR spectrum ($CDCl_3$) of **1f**

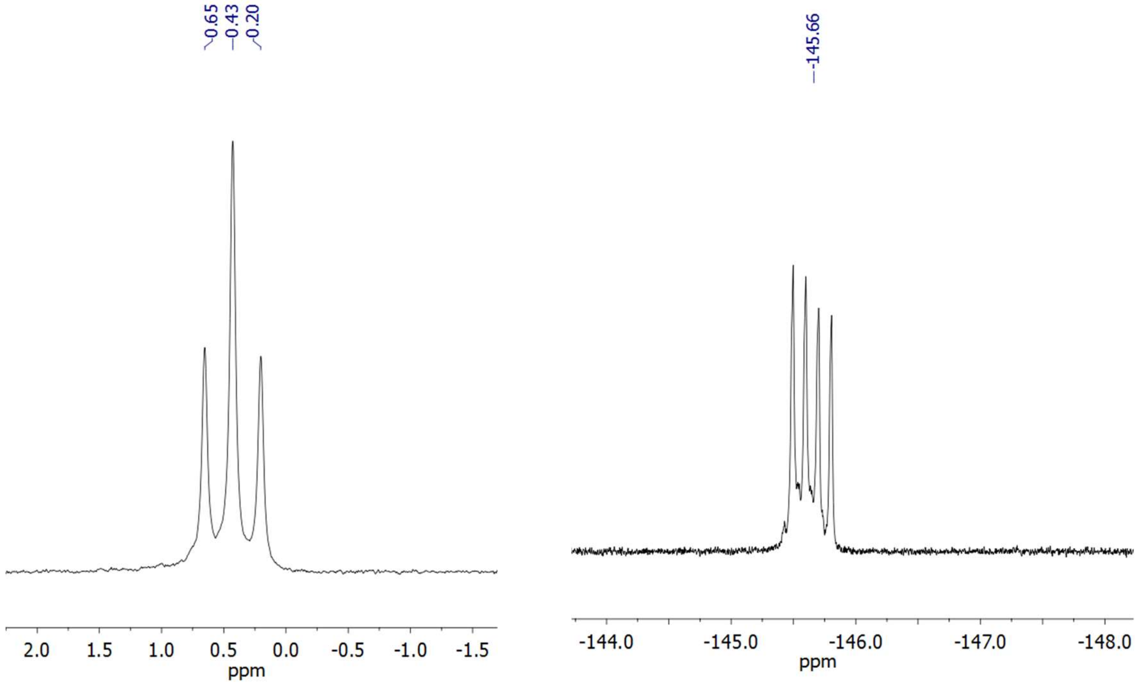

Figure S5.20. ^{11}B-NMR (left) and ^{19}F-NMR (right) spectra ($CDCl_3$) of **1f**

BODIPY **1g**

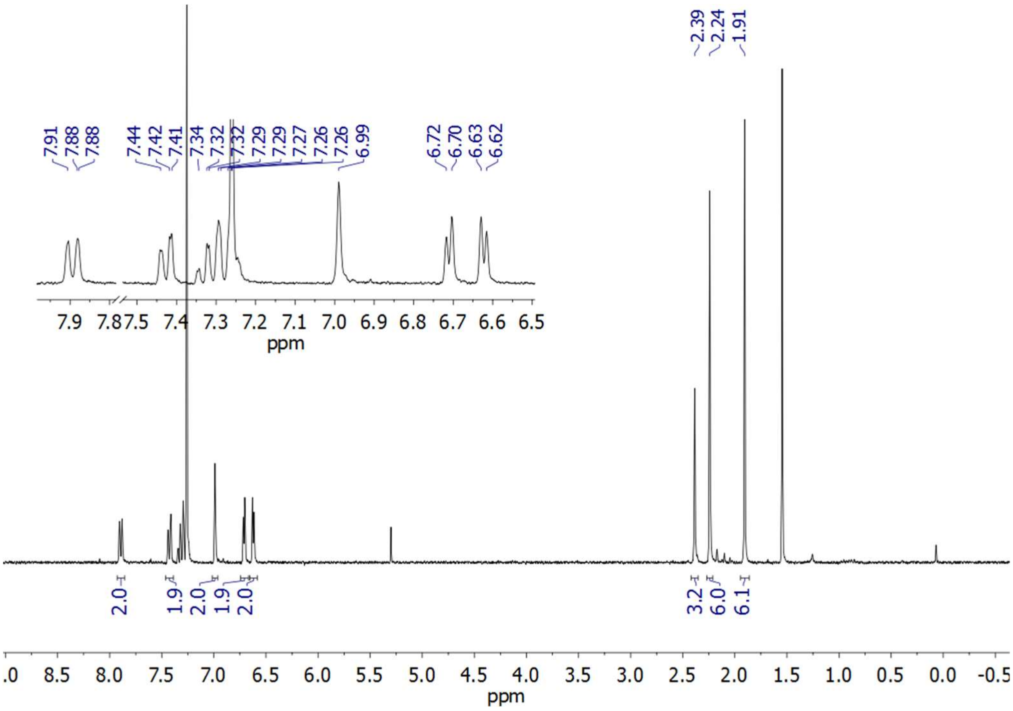

Figure S5.21. ^{1}H-NMR spectrum ($CDCl_3$) of **1g**

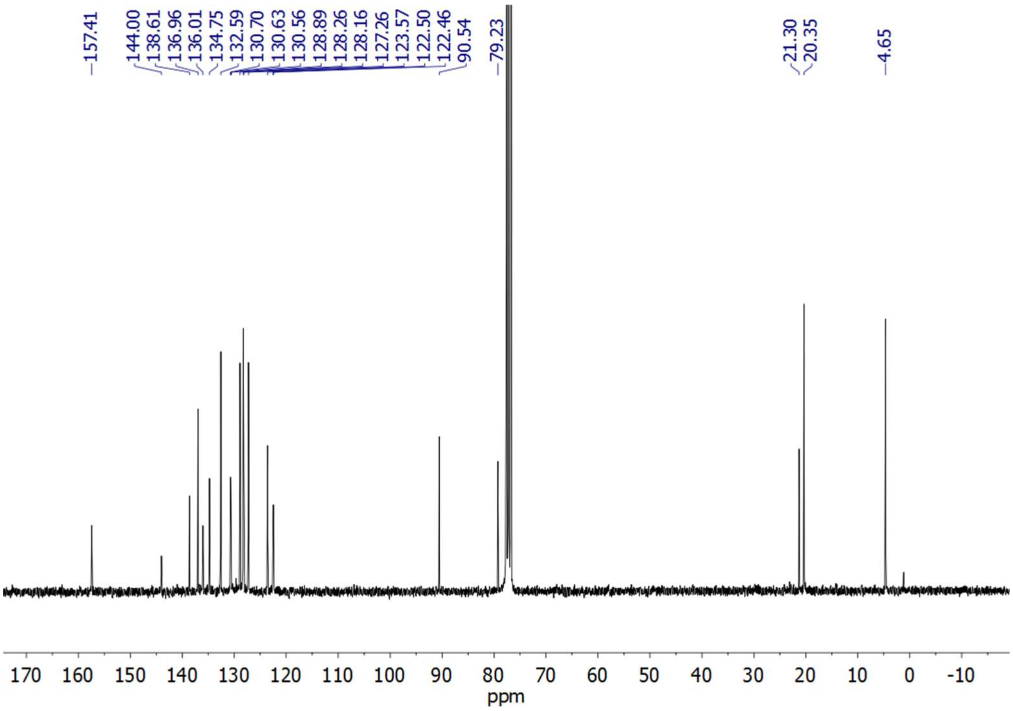

Figure S5.22. ^{13}C-NMR spectrum ($CDCl_3$) of **1g**

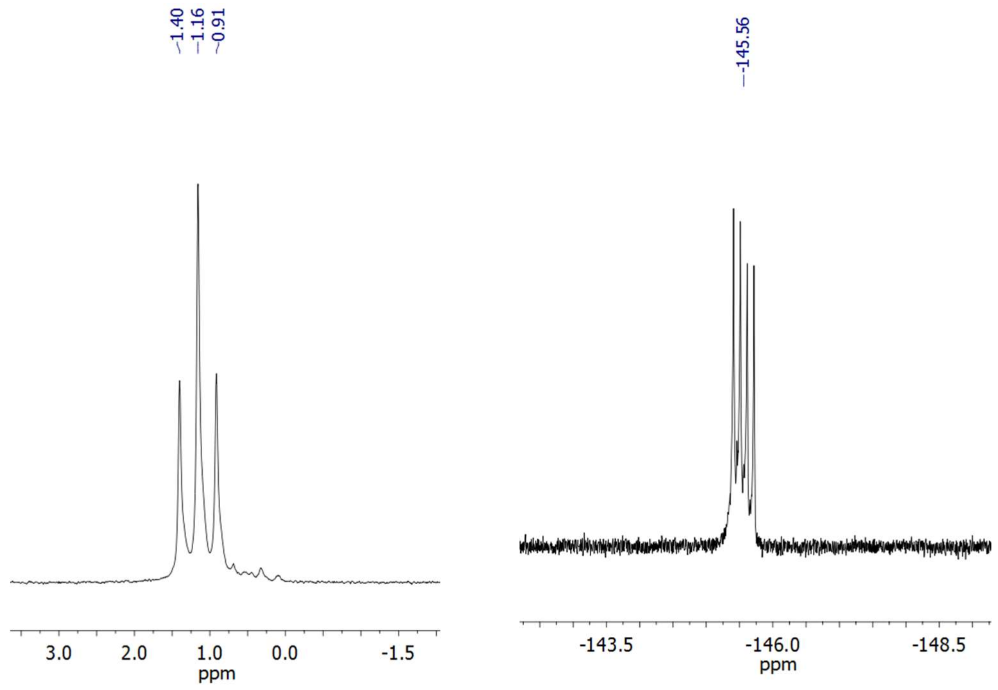

Figure S5.23. ^{11}B-NMR (left) and ^{19}F-NMR (right) spectra ($CDCl_3$) of **1g**

BODIPY **2a**

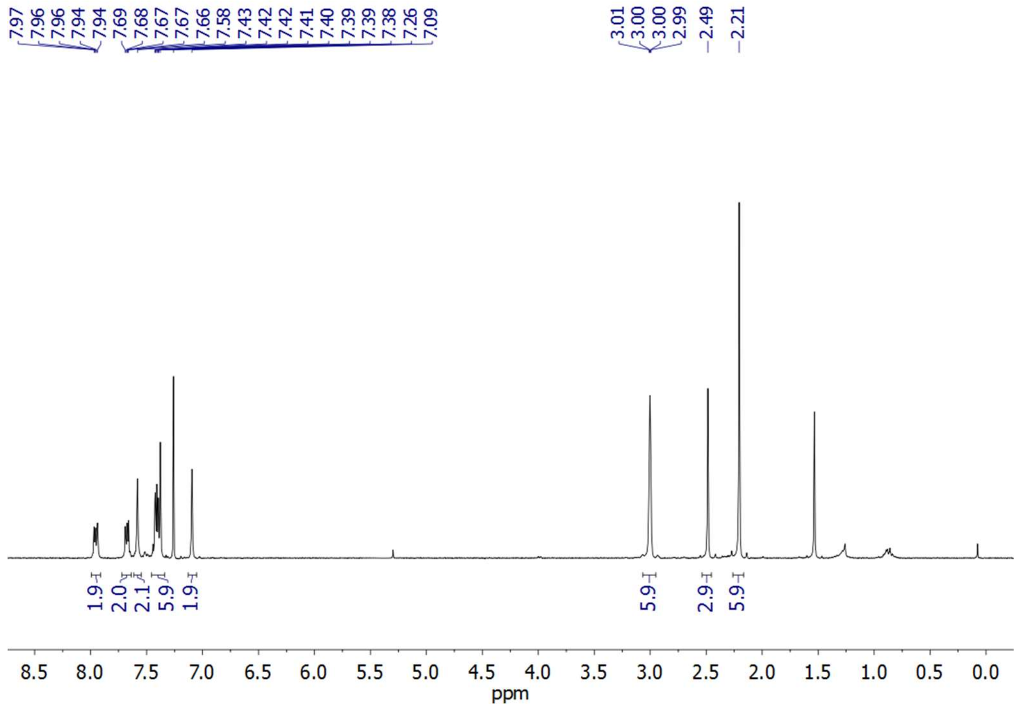

Figure S5.24. ^{1}H-NMR spectrum ($CDCl_3$) of **2a**

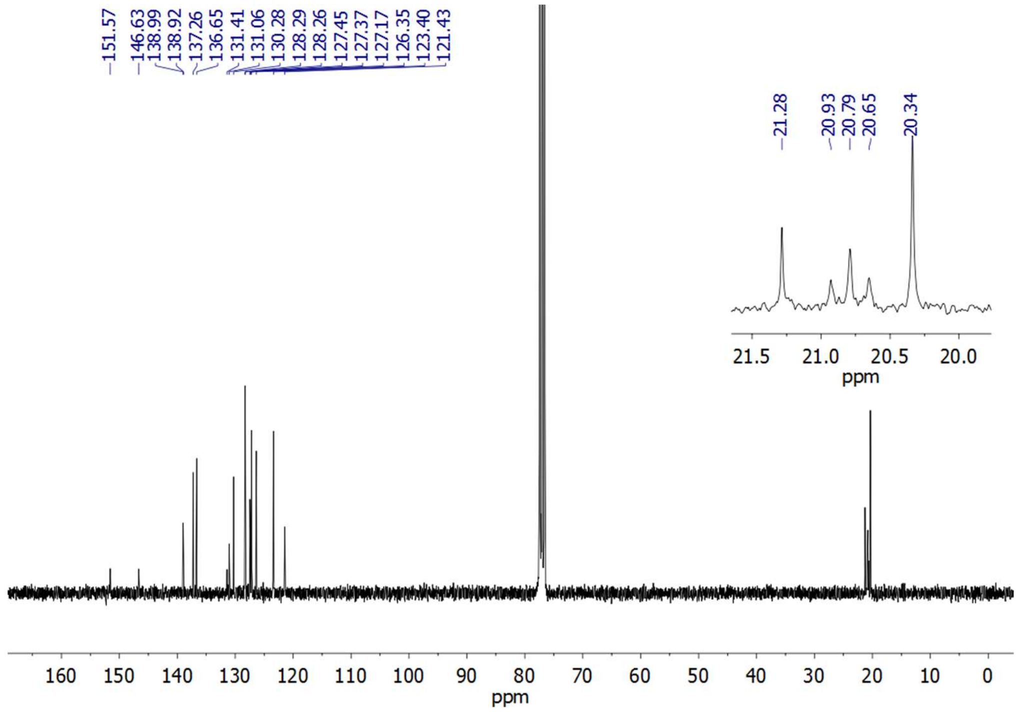

Figure S5.25. ^{13}C-NMR spectrum ($CDCl_3$) of **2a**

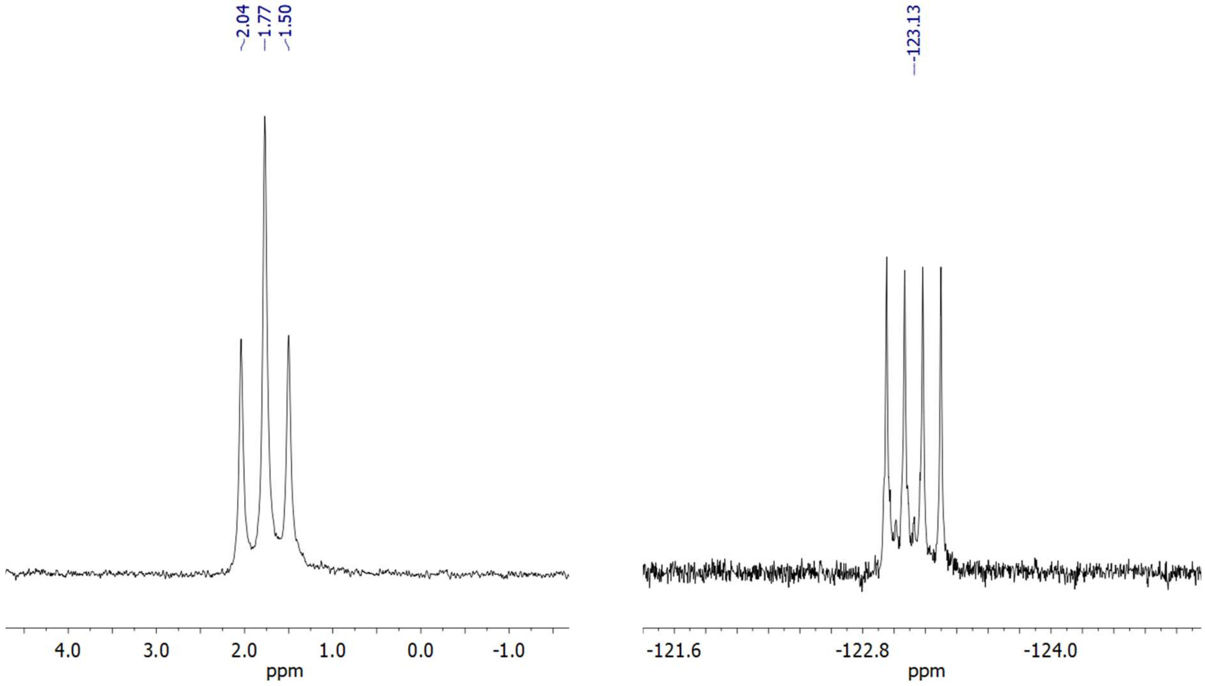

Figure S5.26. ^{11}B-NMR (left) and ^{19}F-NMR (right) spectra ($CDCl_3$) of **2a**

BODIPY **2b**

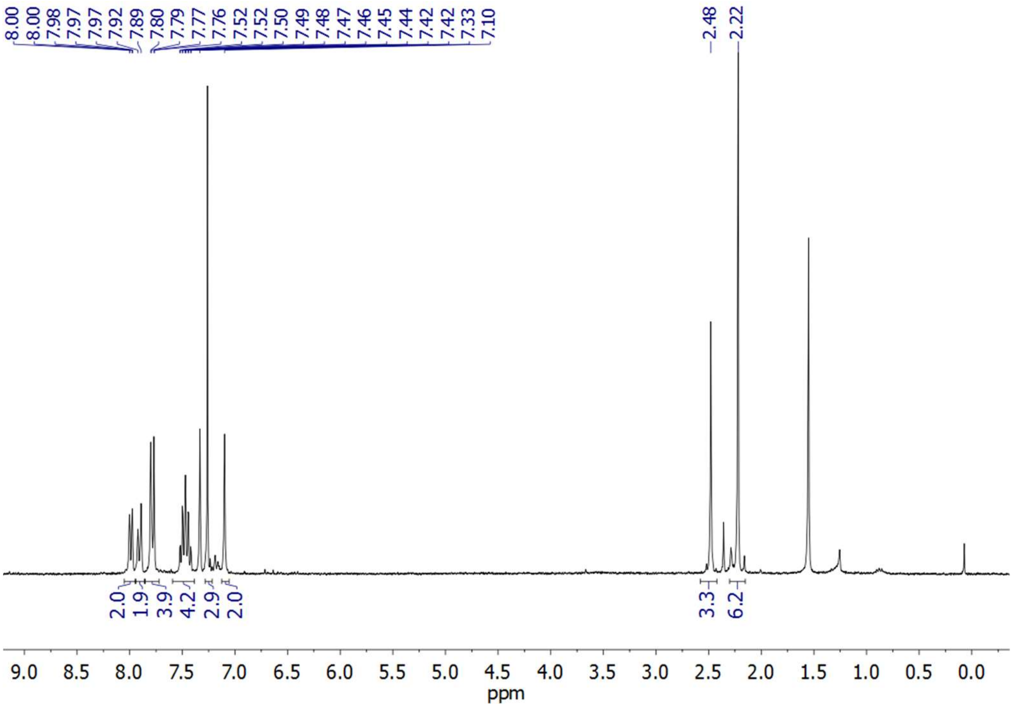

Figure S5.27. ^{1}H-NMR spectrum ($CDCl_3$) of **2b**

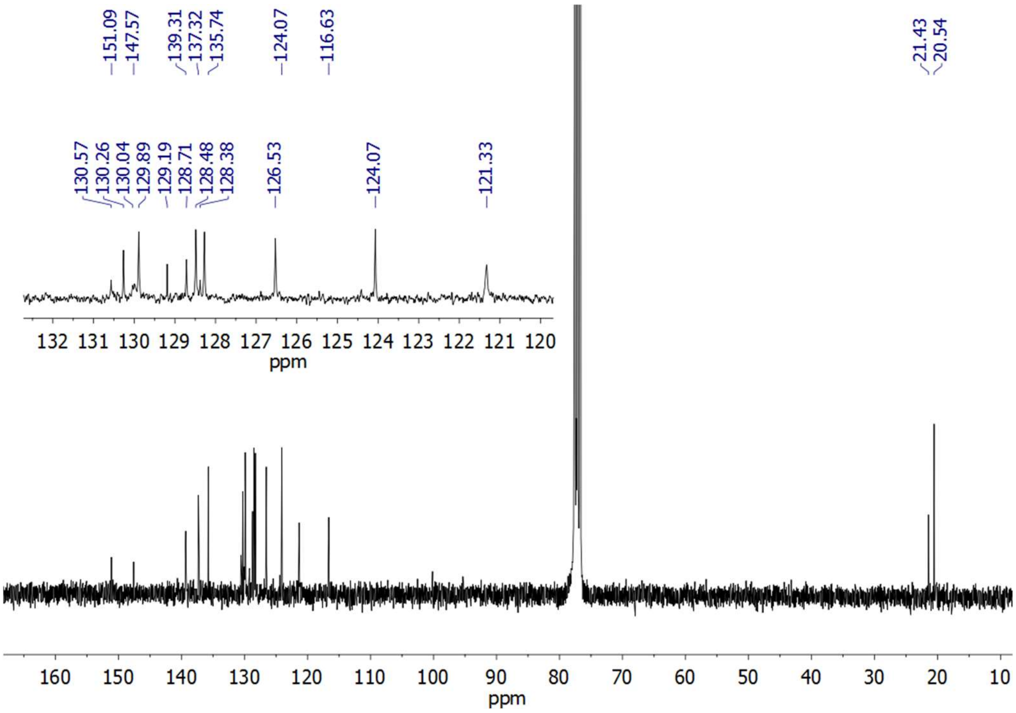

Figure S5.28. ^{13}C-NMR spectrum ($CDCl_3$) of **2b**

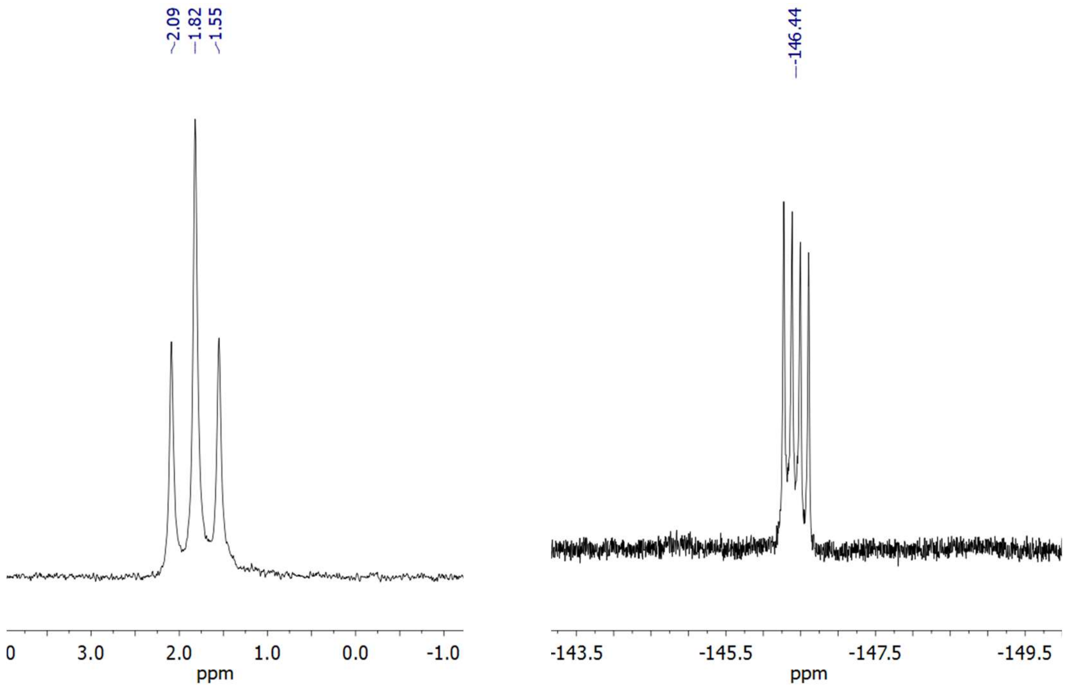

Figure S5.29. ^{11}B-NMR (left) and ^{19}F-NMR (right) spectra ($CDCl_3$) of **2b**

<u>BODIPY **2c**</u>

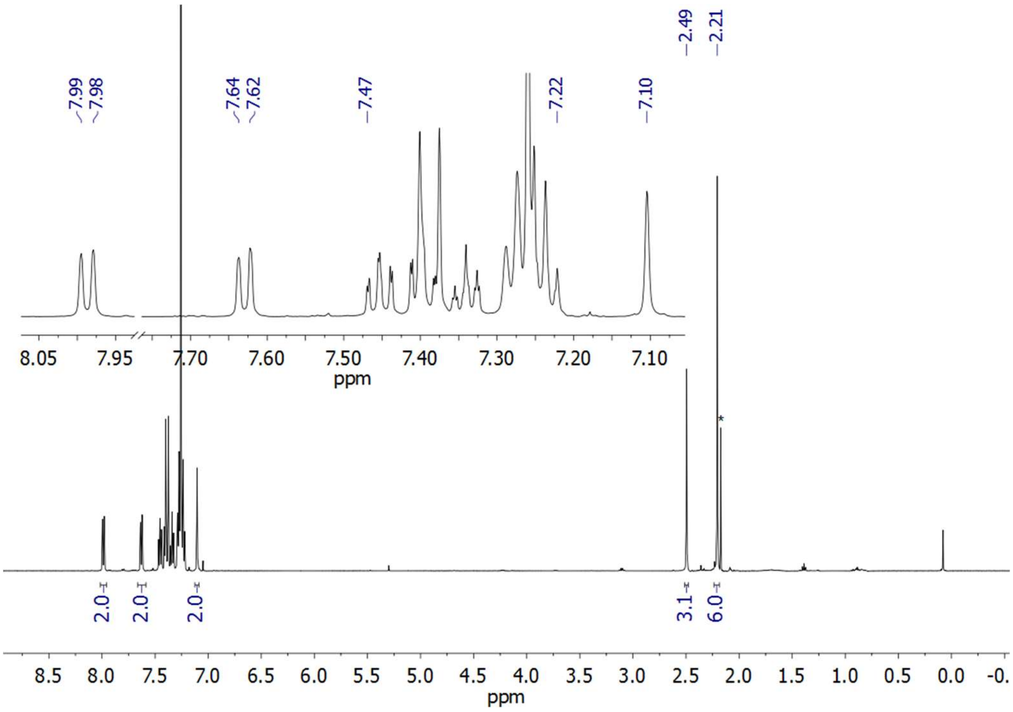

Figure S5.30. ^{1}H-NMR spectrum ($CDCl_3$) of **2c**

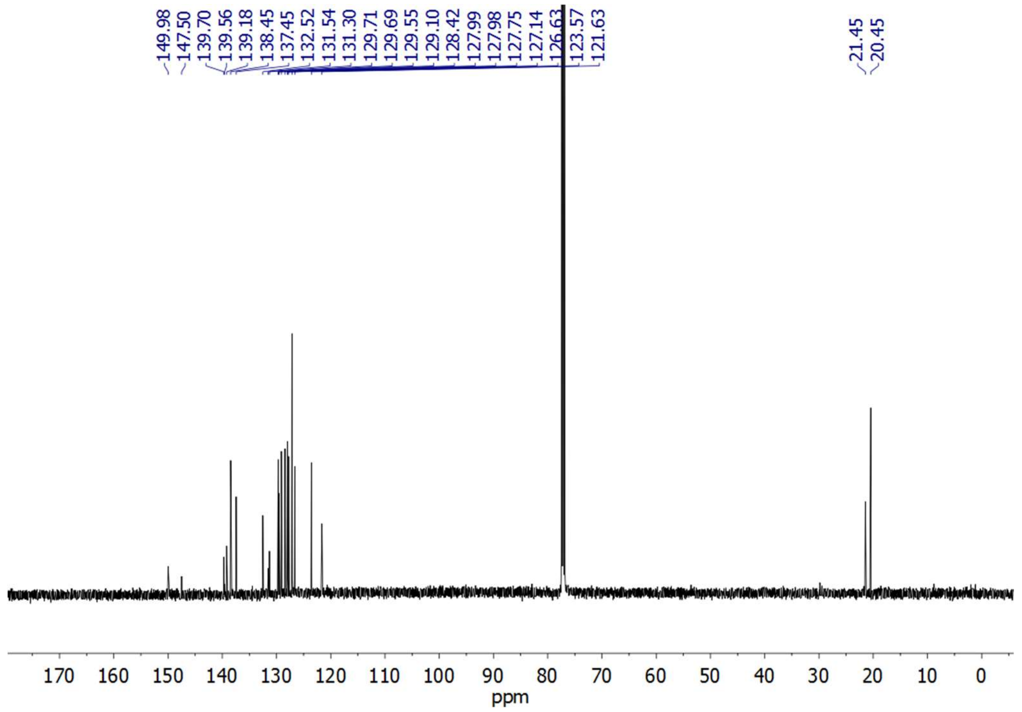

Figure S5.31. ^{13}C-NMR spectrum ($CDCl_3$) of **2c**

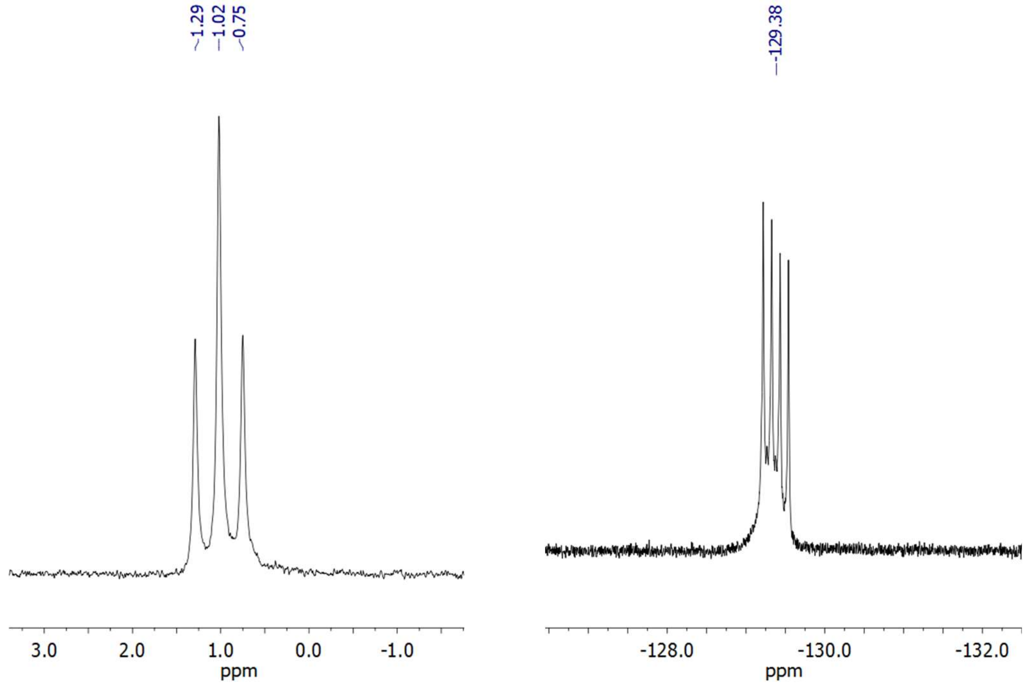

Figure S5.32. ^{11}B-NMR (left) and ^{19}F-NMR (right) spectra ($CDCl_3$) of **2c**

Dipyrromethene **2d´**

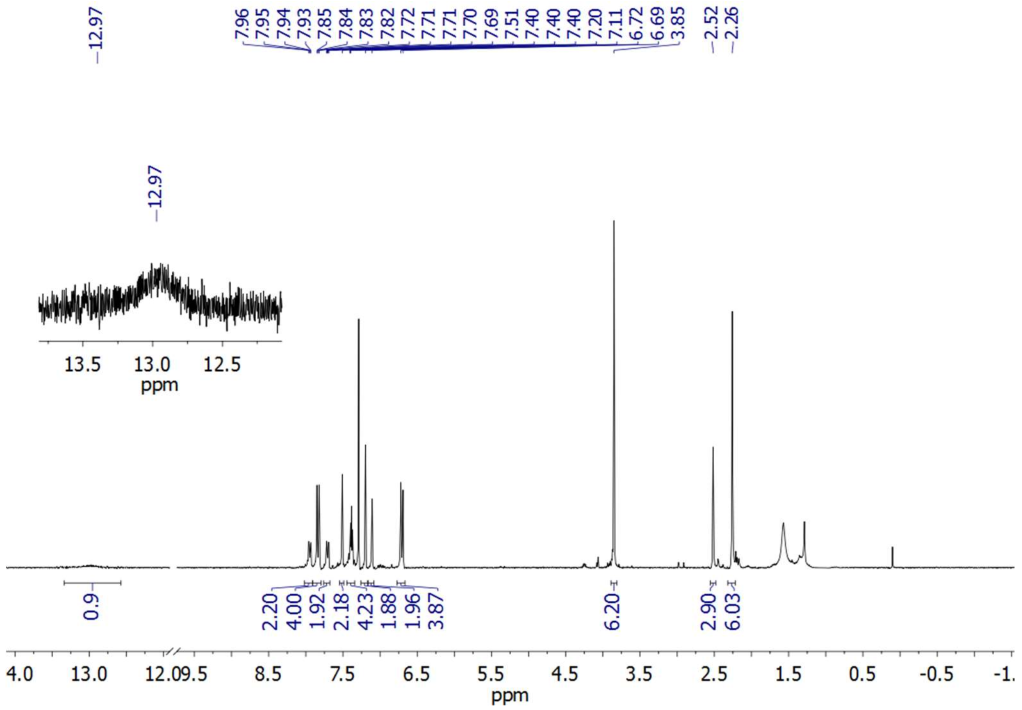

Figure S5.33. ^{1}H-NMR spectrum ($CDCl_3$) of **2d´**

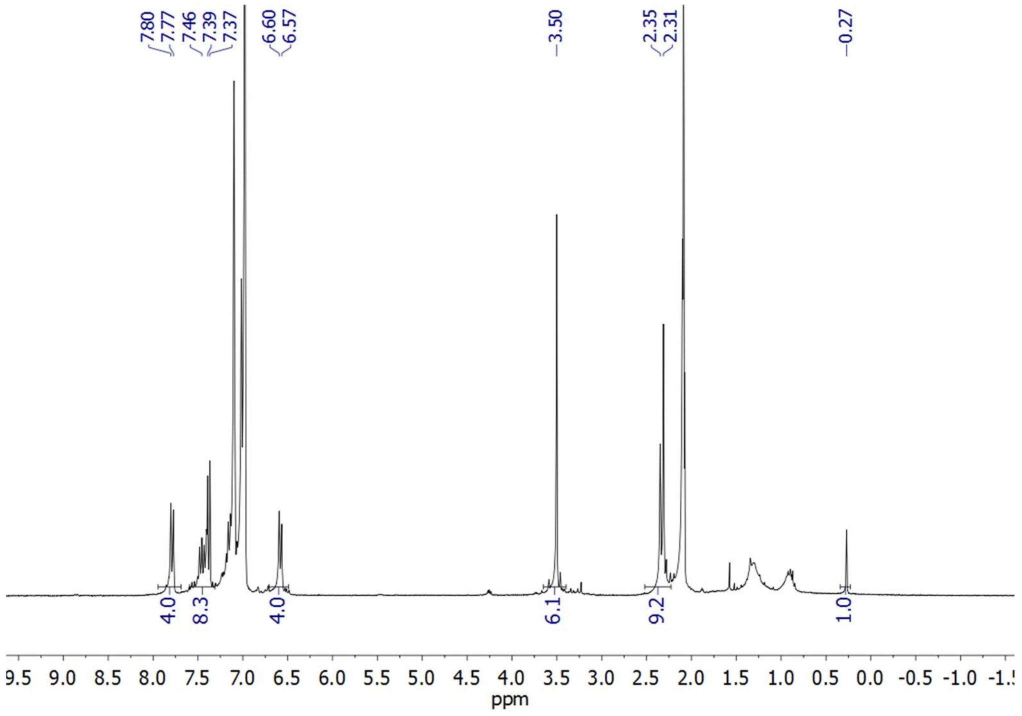

Figure S5.33.2. ^{1}H-NMR spectrum (Toluene-d_8) of **2d´**

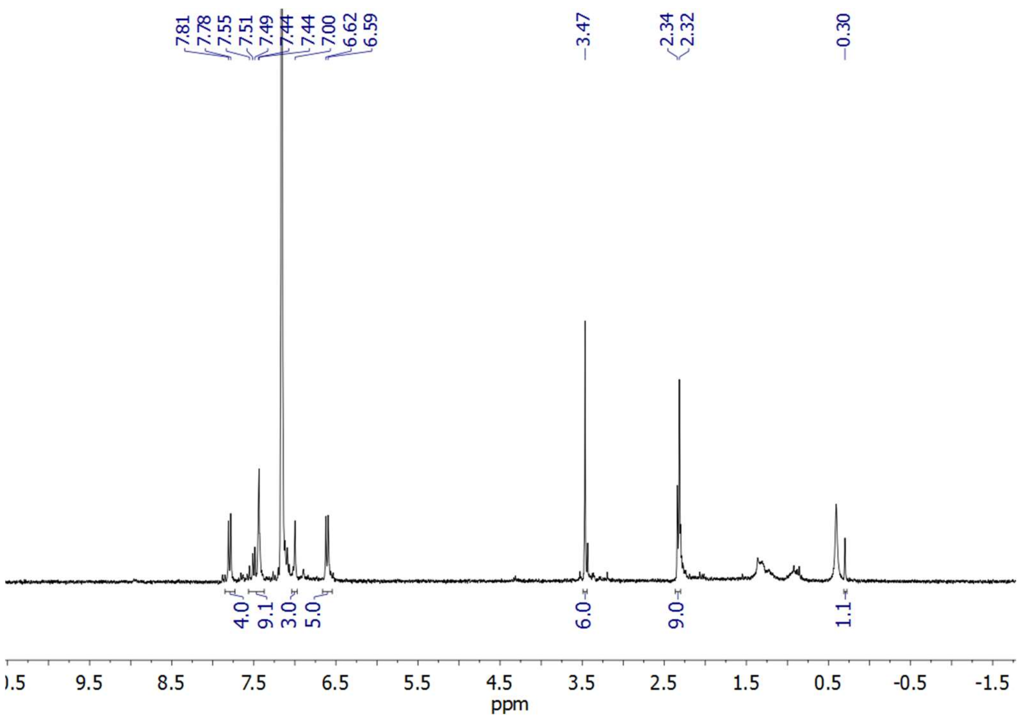

Figure S5.33.3. ^{1}H-NMR spectrum (C_6D_6) of **2d´**

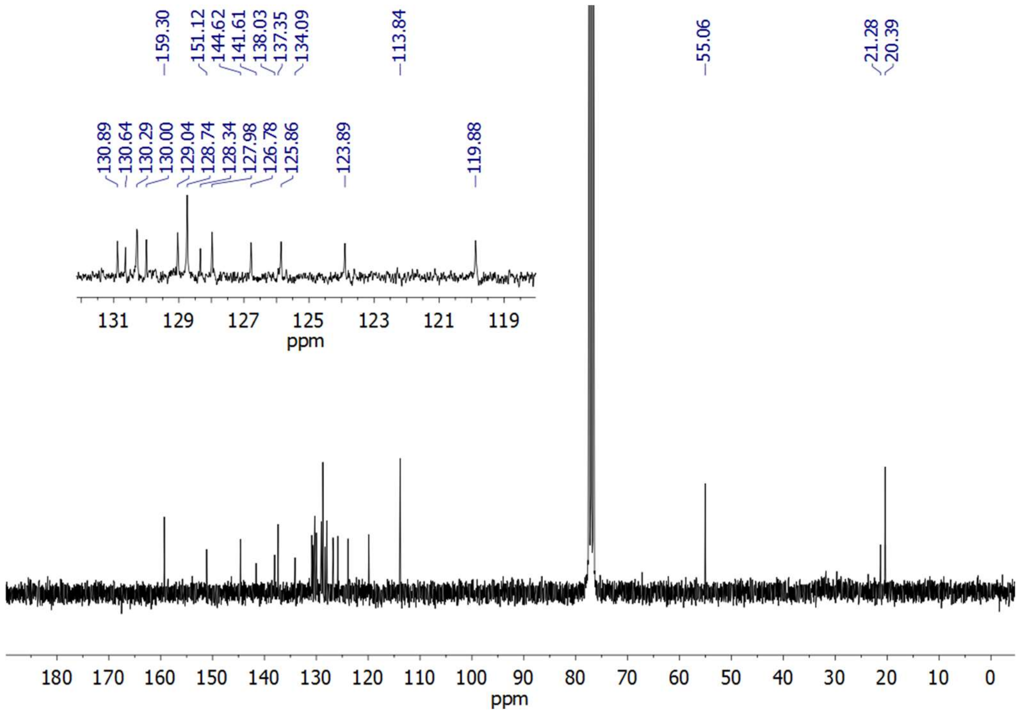

Figure S5.34. ^{13}C-NMR spectrum ($CDCl_3$) of **2d´**

<u>BODIPY **2e**</u>

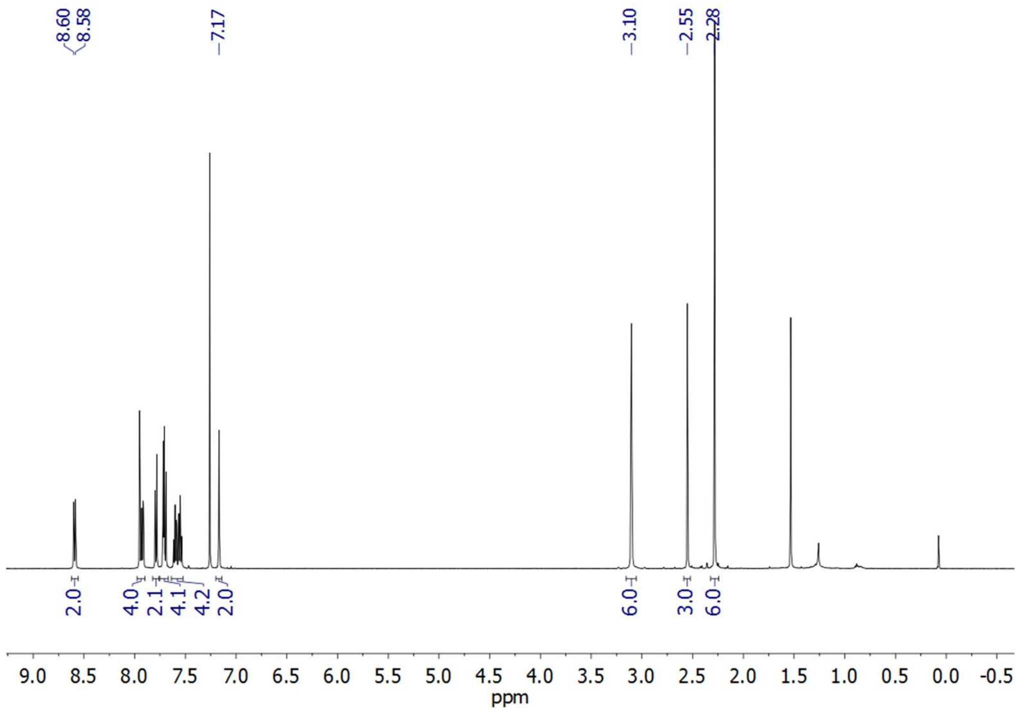

Figure S5.35. ^{1}H-NMR spectrum ($CDCl_3$) of **2e**

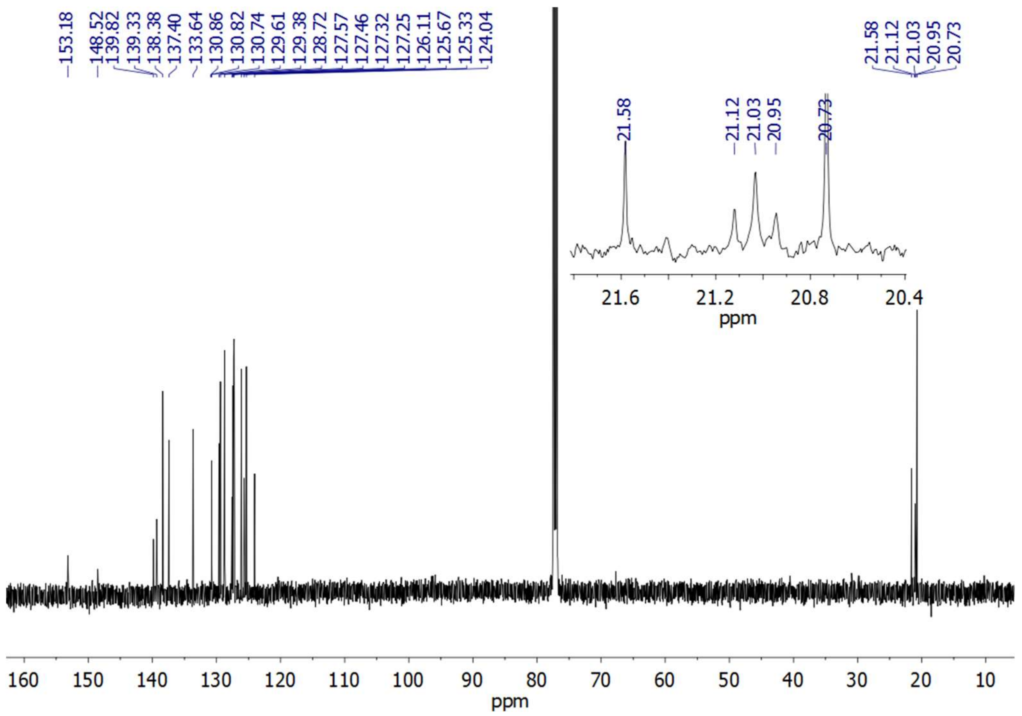

Figure S5.36. ^{13}C-NMR spectrum ($CDCl_3$) of **2e**

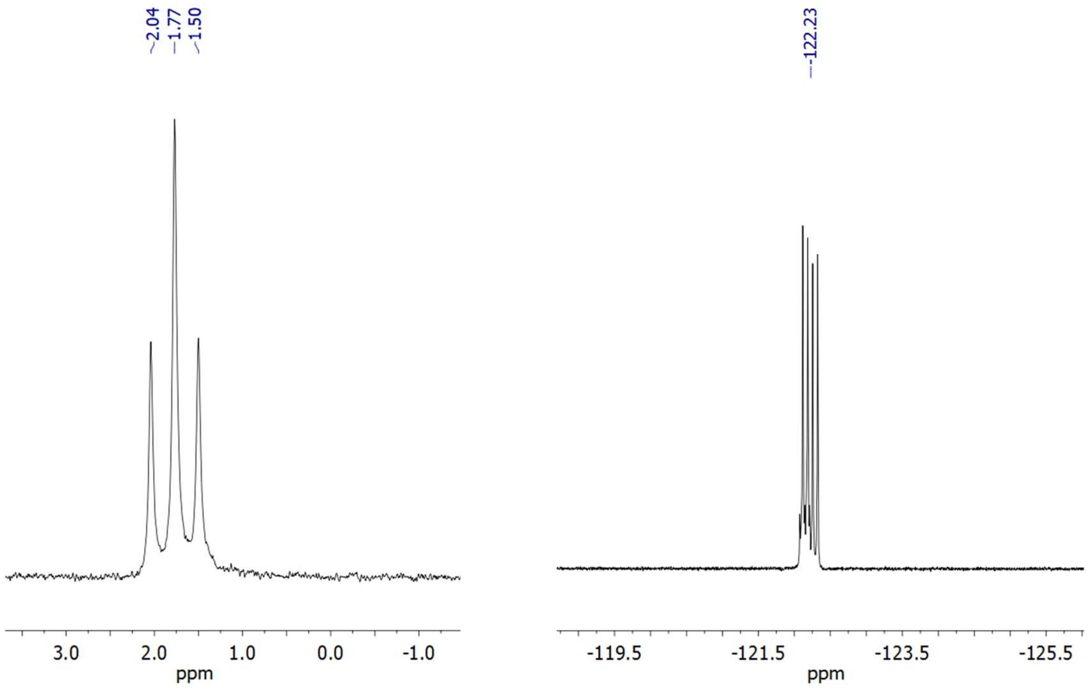

Figure S5.37. ^{11}B-NMR (left) and ^{19}F-NMR (right) spectra ($CDCl_3$) of **2e**

<u>BODIPY **2f**</u>

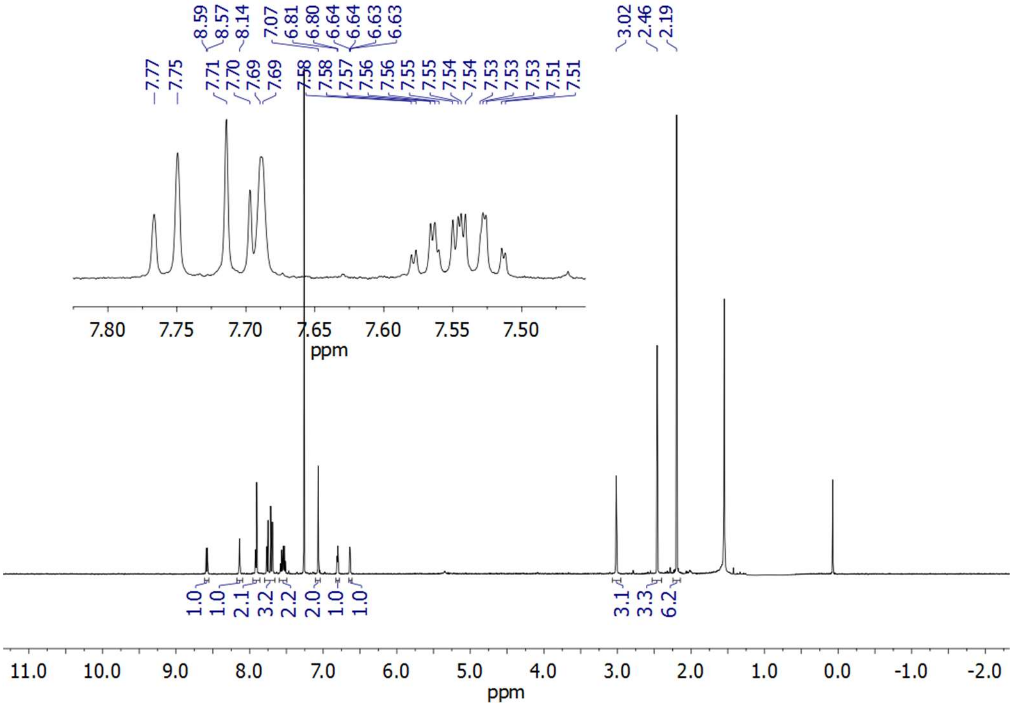

Figure S5.38. ^{1}H-NMR spectrum ($CDCl_3$) of **2f**

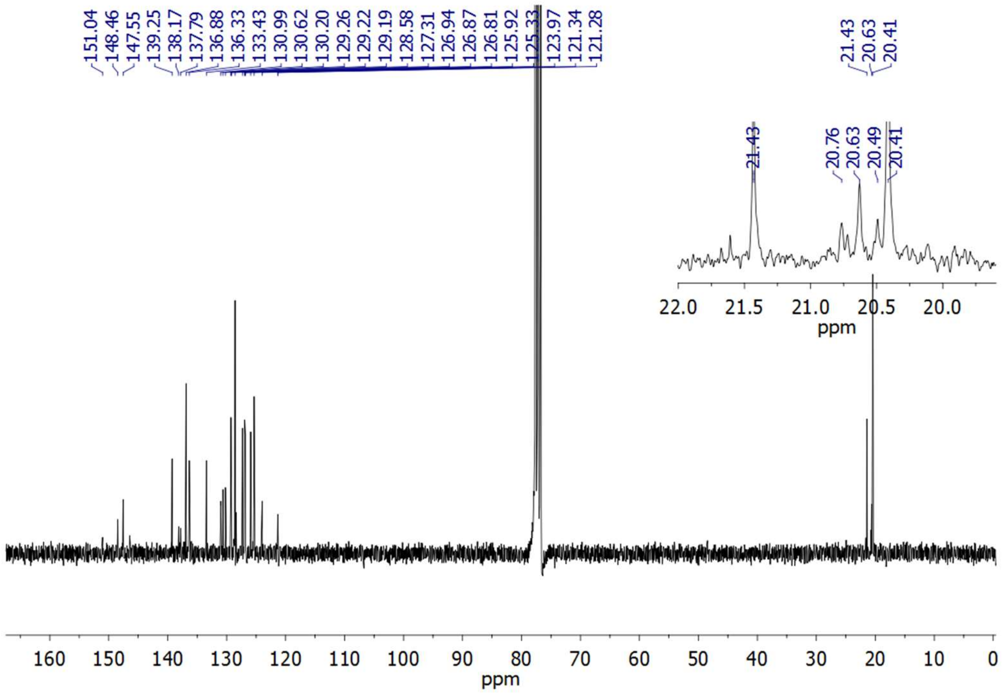

Figure S5.39. ^{13}C-NMR spectrum ($CDCl_3$) of **2f**

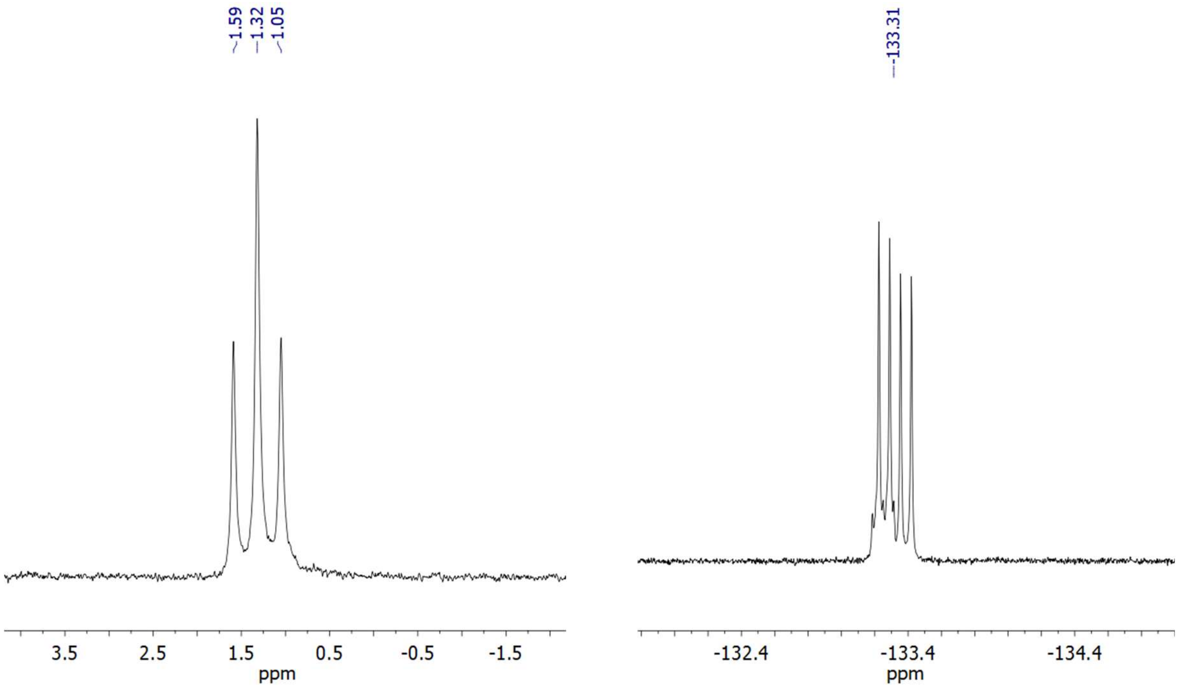

Figure S5.40. ^{11}B-NMR (left) and ^{19}F-NMR (right) spectra ($CDCl_3$) of **2f**

<u>BODIPY **2g**</u>

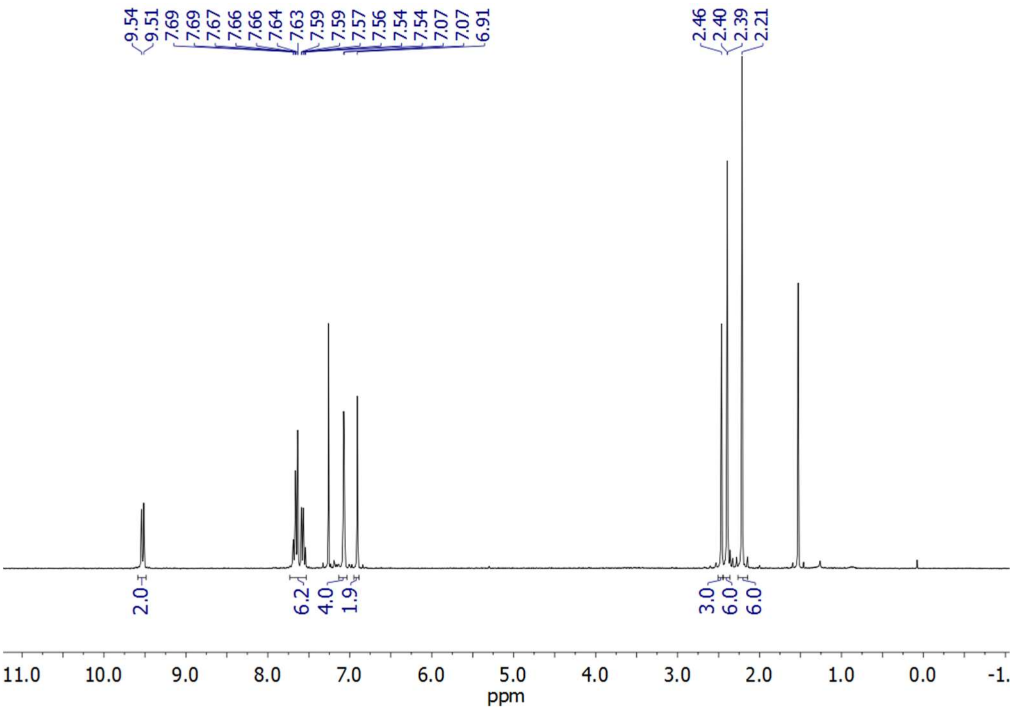

Figure S5.41. ^{1}H-NMR spectrum ($CDCl_3$) of **2g**

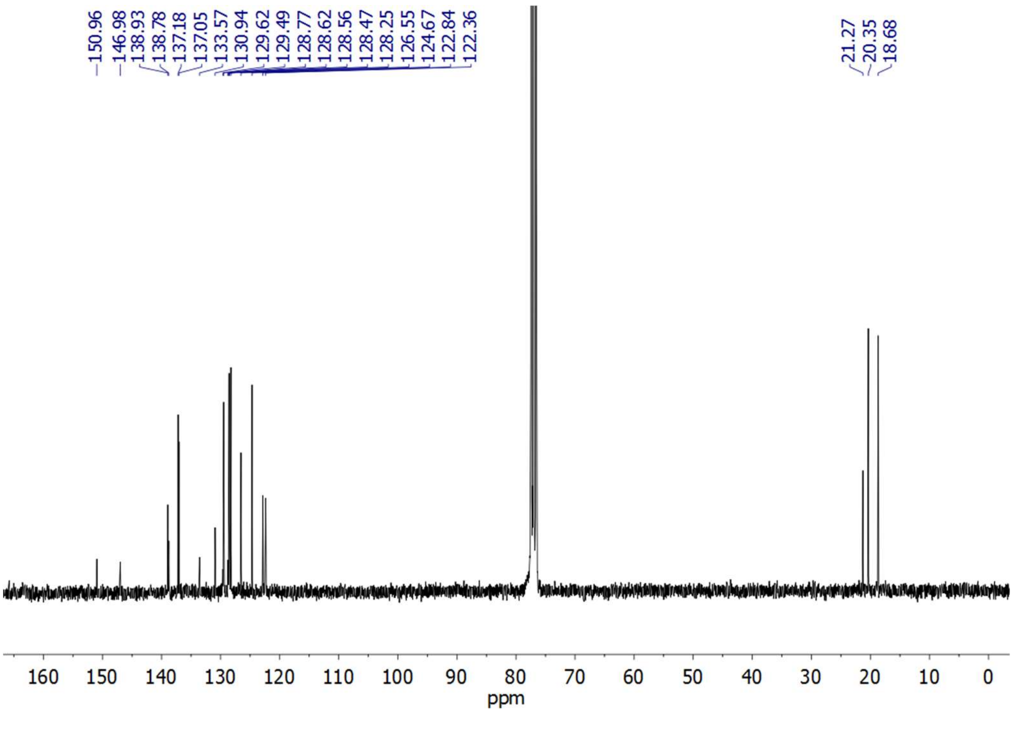

Figure S5.42. ^{13}C-NMR spectrum ($CDCl_3$) of **2g**

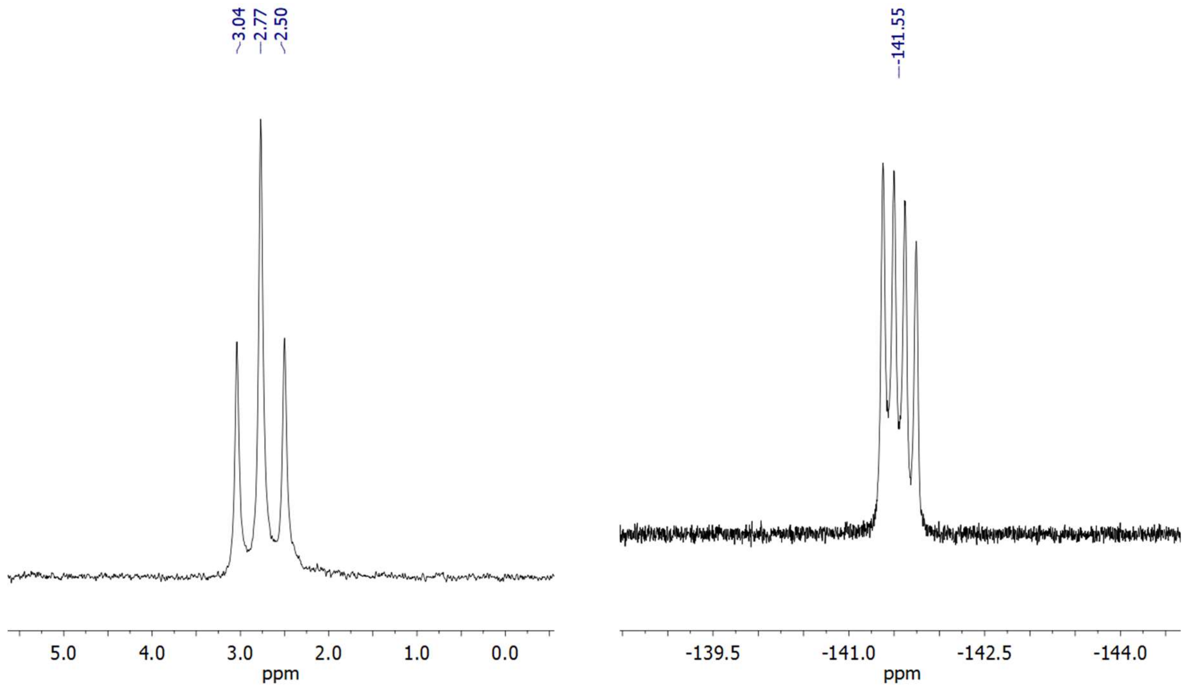

Figure S5.43. ^{11}B-NMR (left) and ^{19}F-NMR (right) spectra ($CDCl_3$) of **2g**

6. Computational Studies

All reported structures were optimized at DFT level using the B3LYP [6] functional and the standard 6-31G(d) basis set for C, N, B, F, P and H and LANL2DZ basis set for Au, which includes the relativistic effective core potential (ECP) of Hay and Wadt and employs a split-valence (double-ζ) basis set.[7] Analytical harmonic frequencies were computed at the same level of theory to confirm the nature of the stationary points. NICS(0) values were calculated at the GIAO-B3lyp/6-31+G(d,p) level. TD-DFT calculations were carried out at the BMK/6-31+G(d,p) and considering the solvent (DCM) through the implicit Polarizable Continuum Model (PCM). For the mechanistic studies, reported energy values correspond to Gibbs Free (G) energies, and include single point refinement of the previous structures by means of the Truhlar´s last generation M06 functional,[8] and the 6-311+G(d,p) basis set for C, N, B, F, P and H, and SDD basis set for Au. Furthermore, transition state geometries were connected with reactants and products *via* Intrinsic Reaction Coordinate (IRC) calculations. Solvent (DCE) effects were also considered through the Model (PCM). All of the calculations were carried out by the methods implemented in Gaussian 16 package.[9]

Origin of **2d** deborylation

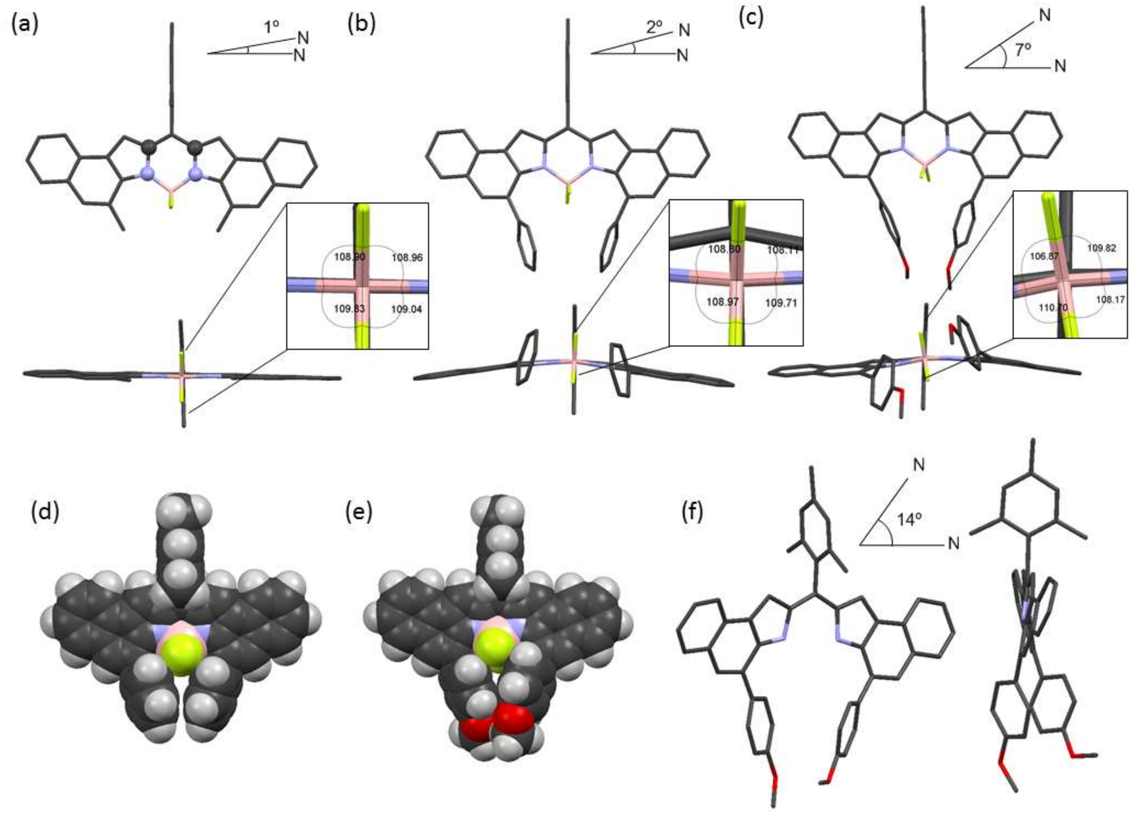

Figure S6.1. (a) X-ray structure of **2a** (a: Front-view; b: bottom-view), (b) calculated structure of **2c** (a: Front-view; b: bottom-view), (c) calculated structure of 2c (a: Front-view; b: bottom-view), van der Waals representation of (d) **2c** and (e) **2d**, and (f) X-ray structure of **2d´** (a: Front-view; b: side-view).

In order to understand de origin of the deborylation reaction of **2d**, the structural differences between **2a**, **2c** and **2d** were explored (in the last two cases, the structures were simulated by DFT calculations). This analysis is based on two structural aspects: a) the discrepancy in the dihedral angle described by the two C-N moieties of the BODIPY core represented as spheres in Figure S6.1a, and b) the N-B-F angles. Concerning the first aspect, the dihedral angles of **2a**, **2c** and **2d** are 1º, 2º, and 7º, respectively. As a result of the steric demand of the methoxy moieties in **2d** (Figure S6.1e) , the resulting bidentate

N-N ligand (dipyrromethene) has not the optimal geometry (ca. 0º) to overlap with the sp^3 orbitals of the BF_2 fragment. Thus, in contrast to its counterparts **2a** and **2c** (angles are nearly 0º), **2d** present a poorer coordinating capability and it is expected to undergo an easier substitution by other nucleophiles. In line with these results and regarding the second aspect, **2a** and **2e** display similar N-B-F angles (2a: 108.9º, 109.9º, 109.8º, 109.0º; and 2e: 108.8º, 109.0º, 109.7º, 108.1º) while those of **2d** are more irregular and oscillates between 106.9º and 110.7º. This result also points out to a more strained coordination around the B atom because of the molecular geometry imposed by the steric congestion. These structural features suggest that the deborylation observed in the compound **2d** arises from the steric hindrance between methoxy groups (see van der Waals models of **2c** and **2d**; Figure S6.1d-e).

<u>NICS(0) calculations</u>

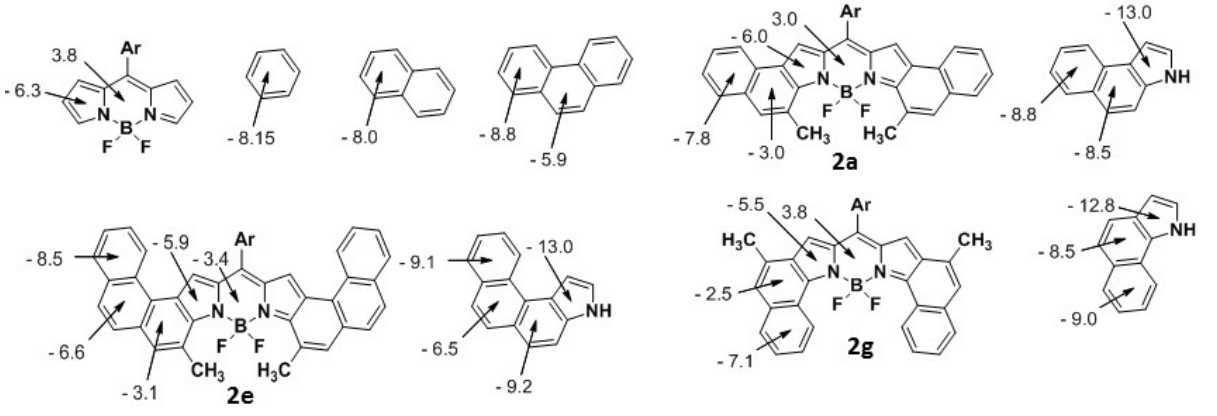

Figure S6.2. NICS(0) values of **2a**, **2e**, **2g** and unsubstituted BODIPY and their corresponding hydrocarbons.

<u>TD-DFT calculations</u>

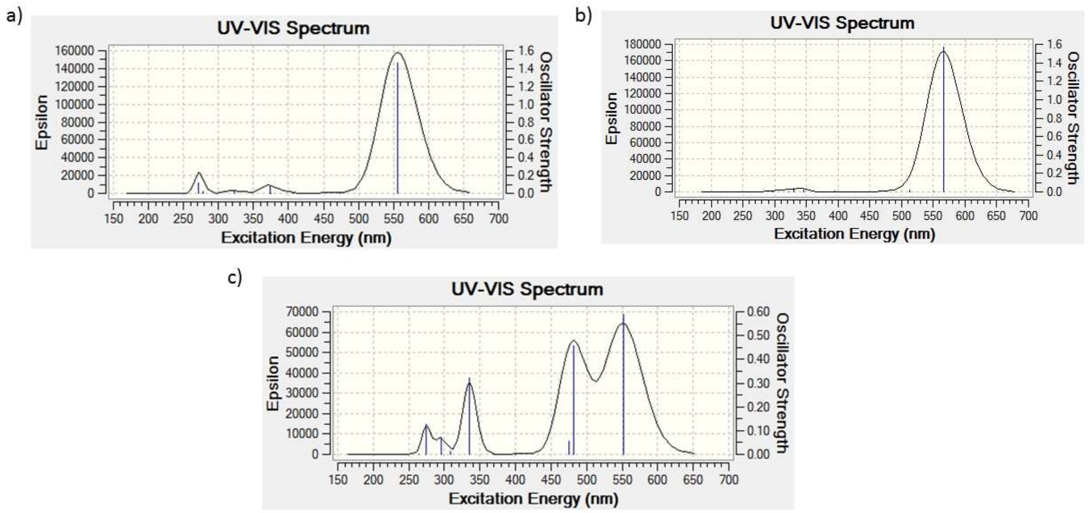

Figure S6.3. Calculated spectra of **2a**, **2e** and **2g**.

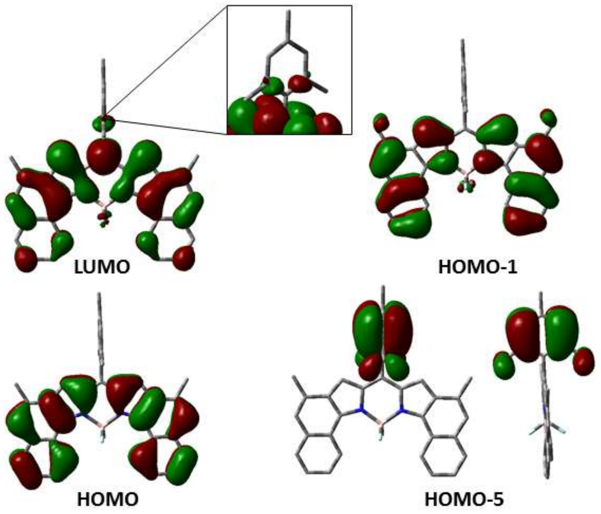

Figure S6.4. Kohn-Sham orbital representations of the orbitals involved in the electronic transitions.

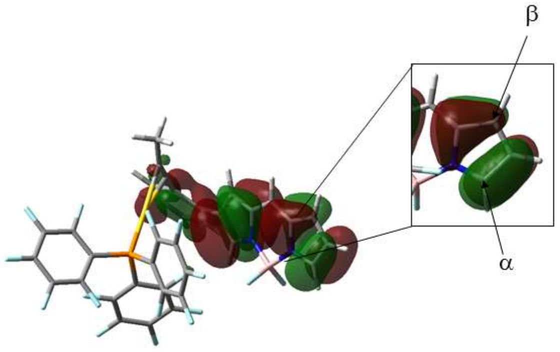

Figure S6.5. Kohn-Sham orbital representations of the HOMO of **Reac$_{\mathbf{H}}$-cat**.

Table S6.1. Energies of the structures involved in the computational study.

	Correction to **G**[a] (B3LYP/6-31G*)	Single Point Energy[a] M06/6-311+G**	Relative **G** energy[b] (M06+B3LYP correction)	Frequency
		**Structures in Figure X. R=H**		
**Reac$_{\mathbf{H}}$-cat.**	0.330815	-3686.093743	0	
**TS1$_{\alpha\text{-H}}$**	0.331811	-3686.070129	15.442	-355.3
**TS1$_{\beta\text{-H}}$**	0.331983	-3686.065611	18.386	-377.6
**IN1$_{\alpha\text{-H}}$**	0.339075	-3686.101475	0.331	
**IN1$_{\beta\text{-H}}$**	0.335744	-3686.097690	0.616	
**TS2$_{\alpha\text{-H}}$**	0.332724	-3686.081957	8.594	-1062.9
**TS2$_{\beta\text{-H}}$**	0.330786	-3686.080295	8.421	
**IN2$_{\alpha\text{-H}}$**	0.33585	-3686.127510	-18.029	
**IN2$_{\beta\text{-H}}$**	0.338387	-3686.121462	-12.642	
**TS3$_{\alpha\text{-H}}$**	0.334323	-3686.109308	-7.566	-868.1
**TS3$_{\beta\text{-H}}$**	0.338201	-3686.107400	-3.934	-807.1
**Prod$_{\alpha\text{-H}}$-cat.**	0.335422	-3686.174765	-47.950	
**Prod$_{\beta\text{-H}}$-cat.**	0.339117	-3686.170191	-42.762	

Structures in Figure X. R=Mes				
**Reac$_{Mes}$-cat.**	0.477654	-4034.351946	0	
**TS1$_{α\text{-}Mes}$**	0.484355	-4034.32564	16.506644	-354.8510
**TS1$_{β\text{-}Mes}$**	0.481395	-4034.322154	18.694323	-368.9220
**IN1$_{α\text{-}Mes}$**	0.485099	-4034.356003	-2.546112	
**IN1$_{β\text{-}Mes}$**	0.488303	-4034.341487	6.563091	
**TS2$_{α\text{-}Mes}$**	0.484800	-4034.336385	9.764207	-1066.7116
**TS2$_{β\text{-}Mes}$**	0.485996	-4034.813099	15.588587	
**IN2$_{α\text{-}Mes}$**	0.486016	-4034.380665	-18.021266	-1022.6213
**IN2$_{β\text{-}Mes}$**	0.493547	-4034.370362	-11.555964	
**TS3$_{α\text{-}Mes}$**	0.482802	-4034.365436	-8.465200	-864.5807
**TS3$_{β\text{-}Mes}$**	0.485249	-4034.352442	-0.311089	-818.3965
**Prod$_{α\text{-}Mes}$-cat.**	0.488092	-4034.426499	-46.781944	
**Prod$_{β\text{-}Mes}$-cat.**	0.492354	-4034.411951	-37.652974	

[a]Energy units: Hartrees. [b]Energy units: kcal/mol

Cartesian coordinates of **2a**, **2e** and **2g**

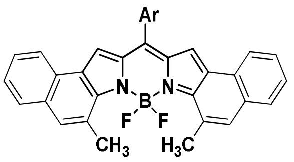

Standard orientation:

Atomic Number	Atomic Type	Coordinates (Angstroms) X	Y	Z
7	0	-1.271300	-1.111834	-0.000220
6	0	-1.213939	0.282693	0.000553
6	0	-0.000178	0.983188	0.001011
6	0	1.213844	0.283137	0.000888
7	0	1.271728	-1.111365	0.000035
6	0	2.598577	-1.463499	0.000121
6	0	3.398999	-0.265001	0.001133
6	0	2.520043	0.813488	0.001622
6	0	-2.598023	-1.464462	-0.000441
6	0	-3.398886	-0.266255	0.000290
6	0	-2.520330	0.812561	0.000823
6	0	-0.000535	2.479975	0.001472
6	0	-0.002657	3.179676	1.225434
6	0	0.003835	3.180868	-1.224909
6	0	-0.000963	4.579603	1.198825
6	0	0.001300	5.298307	0.000989
6	0	0.005435	4.578203	-1.199094
6	0	0.009096	2.451448	-2.550082
6	0	-0.004820	2.452676	2.551960
6	0	-0.012764	6.809170	-0.005605
5	0	0.000388	-2.025570	-0.000781
9	0	0.000696	-2.811719	-1.157586
9	0	0.000398	-2.813417	1.154832
6	0	-4.838420	-0.315365	0.000359
6	0	-5.435760	-1.605950	-0.000456
6	0	-4.597494	-2.771593	-0.001315
6	0	-3.220862	-2.760474	-0.001356
6	0	-5.663650	0.826818	0.001197
6	0	-7.044518	0.697752	0.001230
6	0	-7.639919	-0.578045	0.000415
6	0	-6.845195	-1.712205	-0.000422
6	0	3.221877	-2.759280	-0.000628
6	0	4.598514	-2.769903	-0.000308
6	0	5.436358	-1.603953	0.000728
6	0	4.838550	-0.313584	0.001499
6	0	5.663359	0.828903	0.002542
6	0	7.044274	0.700337	0.002802
6	0	7.640140	-0.575242	0.002018
6	0	6.845831	-1.709694	0.000995
1	0	2.756747	1.867983	0.002526
1	0	-2.757407	1.866970	0.001389
1	0	-0.000828	5.119538	2.143194
1	0	0.010838	5.117894	-2.143949
1	0	0.006188	3.160629	-3.382623
1	0	-0.867917	1.802515	-2.657152
1	0	0.893232	1.811895	-2.655468
1	0	0.873022	1.804871	2.659067
1	0	-0.888170	1.812477	2.659935
1	0	-0.001474	3.163358	3.383222
1	0	0.757636	7.212684	-0.672882
1	0	0.159008	7.214871	0.996067
1	0	-0.977151	7.195032	-0.360118
1	0	-5.099173	-3.736784	-0.001997
1	0	-5.213247	1.815459	0.001847
1	0	-7.669552	1.586341	0.001898
1	0	-8.722208	-0.671041	0.000446
1	0	-7.298734	-2.700433	-0.001053
1	0	5.100544	-3.734911	-0.000880
1	0	5.212599	1.817382	0.003158
1	0	7.668983	1.589155	0.003618
1	0	8.722463	-0.667843	0.002218
1	0	7.299732	-2.697756	0.000389
6	0	-2.472605	-4.069624	-0.002336
6	0	2.474054	-4.068677	-0.001773
1	0	1.824970	-4.156463	0.873097
1	0	1.825225	-4.155064	-0.876974
1	0	3.182189	-4.902050	-0.002338
1	0	-3.180472	-4.903225	-0.003674
1	0	-1.823080	-4.155537	-0.877057
1	0	-1.824165	-4.157463	0.873010

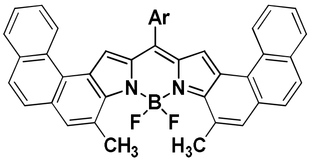

Standard orientation:

Atomic Number	Atomic Type	Coordinates (Angstroms) X	Y	Z
7	0	1.272570	-1.506210	0.016302
6	0	1.212653	-0.116900	0.008550
6	0	0.000154	0.586203	0.000076
6	0	-1.212542	-0.116589	-0.008443
7	0	-1.272869	-1.505878	-0.015965
6	0	-2.601240	-1.853675	-0.031754
6	0	-3.415140	-0.650453	-0.002262
6	0	-2.513947	0.418334	-0.011188
6	0	2.600841	-1.854386	0.031999
6	0	3.415083	-0.651410	0.002119
6	0	2.514208	0.417651	0.011011
6	0	0.000450	2.082757	0.000206
6	0	0.021338	2.782923	-1.224837
6	0	-0.025294	2.782733	1.225722
6	0	0.016360	4.181690	-1.198201
6	0	-0.004369	4.900697	0.000599
6	0	-0.027931	4.181125	1.199460
6	0	-0.050919	2.054252	2.551175
6	0	0.041694	2.055141	-2.550769
6	0	0.022115	6.411264	0.002322
5	0	-0.000277	-2.420649	0.000313
9	0	-0.024950	-3.204789	1.157446
9	0	0.024195	-3.205143	-1.156594
6	0	4.859450	-0.714480	0.027712
6	0	5.410779	-2.002045	0.222844
6	0	4.570625	-3.159343	0.240423
6	0	3.200396	-3.150806	0.117850
6	0	5.759579	0.412656	-0.117782
6	0	7.163232	0.211075	0.108332
6	0	7.660652	-1.097752	0.385703
6	0	6.818097	-2.169865	0.402371
6	0	-3.201158	-3.149943	-0.117298
6	0	-4.571376	-3.158114	-0.239993
6	0	-5.411197	-2.000564	-0.222875
6	0	-4.859527	-0.713091	-0.028088
6	0	-5.759357	0.414372	0.116810
6	0	-7.163036	0.213097	-0.109446
6	0	-7.660789	-1.095696	-0.386367
6	0	-6.818537	-2.168053	-0.402531
1	0	-2.712935	1.475156	-0.066410
1	0	2.713491	1.474427	0.066005
1	0	0.027521	4.721683	-2.142581
1	0	-0.050198	4.720820	2.143867
1	0	-0.064383	2.764140	3.382943
1	0	0.826559	1.408680	2.674033
1	0	-0.934472	1.411798	2.642324
1	0	-0.837106	1.411016	-2.671815
1	0	0.924008	1.411367	-2.644485
1	0	0.054301	2.765459	-3.382182
1	0	-0.524072	6.820089	0.858865
1	0	-0.418586	6.820275	-0.912601
1	0	1.051770	6.787776	0.064772
1	0	5.067477	-4.120948	0.347295
6	0	5.351590	1.712801	-0.519963
6	0	8.057458	1.307299	0.019165
1	0	8.727017	-1.226272	0.552005
1	0	7.202150	-3.172362	0.572042
1	0	-5.068500	-4.119606	-0.346618
6	0	-5.351075	1.714594	0.518450
6	0	-8.056968	1.309612	-0.020908
1	0	-8.727167	-1.223974	-0.552773
1	0	-7.202851	-3.170502	-0.571898
6	0	2.453160	-4.460323	0.104677
6	0	-2.454295	-4.459667	-0.103643
1	0	-1.766249	-4.537535	-0.948541
1	0	-1.847653	-4.560809	0.799689
1	0	-3.164071	-5.290496	-0.147107
1	0	3.162712	-5.291342	0.148162
1	0	1.765304	-4.537798	0.949769
1	0	1.846260	-4.561503	-0.798473
6	0	7.613187	2.566553	-0.323619
6	0	6.247987	2.759547	-0.618094
6	0	-6.247183	2.761647	0.615934
6	0	-7.612394	2.568901	0.321351
1	0	9.112651	1.129063	0.211559
1	0	8.310175	3.396832	-0.393045
1	0	5.895980	3.736185	-0.938511
1	0	4.327133	1.890983	-0.811183
1	0	-8.309150	3.399417	0.390264
1	0	-5.894922	3.738343	0.935886
1	0	-4.326623	1.892672	0.809726
1	0	-9.112181	1.131585	-0.213390

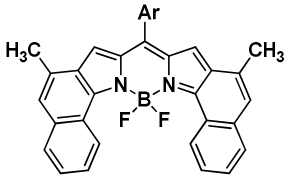

Standard orientation:

Atomic Number	Atomic Type	Coordinates (Angstroms) X	Y	Z
7	0	1.269426	-0.576325	0.050904
6	0	1.218084	0.819218	0.008685
6	0	0.006708	1.523363	-0.000138
6	0	-1.210914	0.830058	-0.008782
7	0	-1.274724	-0.564970	-0.051309
6	0	-2.601124	-0.913753	-0.038289
6	0	-3.395435	0.284830	0.029772
6	0	-2.514156	1.363214	0.035762
6	0	2.592655	-0.936949	0.038447
6	0	3.397681	0.254533	-0.028668
6	0	2.526056	1.340725	-0.035059
6	0	0.013622	3.020154	-0.000357
6	0	0.056864	3.720234	1.224201
6	0	-0.028128	3.720185	-1.224959
6	0	0.057287	5.118821	1.197821
6	0	0.022004	5.838130	-0.000694
6	0	-0.023103	5.118792	-1.198829
6	0	-0.077464	2.992053	-2.549954
6	0	0.093872	2.992127	2.549605
6	0	0.056456	7.348578	-0.001798
5	0	-0.006715	-1.480323	-0.000305
9	0	-0.076965	-2.278523	1.145324
9	0	0.056493	-2.278672	-1.146339
6	0	4.832159	0.210543	-0.094598
6	0	5.414595	-1.021532	-0.098022
6	0	4.655076	-2.243061	-0.010303
6	0	3.221584	-2.235116	0.081265
6	0	-3.241635	-2.206210	-0.081537
6	0	-4.675047	-2.201448	0.011417

6	0	-5.423557	-0.973250	0.100577
6	0	-4.830184	0.253595	0.097008
1	0	2.774720	2.391431	-0.076226
1	0	0.084611	5.658779	2.141961
1	0	-0.056677	5.658665	-2.142825
1	0	0.782274	2.323544	-2.675242
1	0	-0.977828	2.372810	-2.637495
1	0	-0.075833	3.701639	-3.382149
1	0	0.987219	2.363192	2.639835
1	0	0.097770	3.702027	3.381525
1	0	-0.408714	7.760176	0.899924
1	0	1.089516	7.719753	-0.032855
1	0	-0.462109	7.760156	-0.873896
6	0	2.549360	-3.473895	0.218490
6	0	3.248448	-4.668916	0.231844
6	0	4.648929	-4.677867	0.113634
6	0	5.334440	-3.480824	0.001570
6	0	-2.580596	-3.450786	-0.220595
6	0	-3.290303	-4.639529	-0.234128
6	0	-4.690681	-4.636114	-0.114360
6	0	-5.365409	-3.433108	-0.000696
6	0	5.622911	1.489293	-0.167850
6	0	-5.609437	1.539305	0.171471
1	0	6.497063	-1.108596	-0.157815
1	0	-6.506687	-1.050683	0.161507
1	0	1.478576	-3.491213	0.345398
1	0	2.706583	-5.604082	0.341043
1	0	5.192164	-5.618761	0.121484
1	0	6.418915	-3.477378	-0.073977
1	0	-1.510170	-3.477416	-0.349018
1	0	-2.756920	-5.579393	-0.344757
1	0	-5.242275	-5.572129	-0.122360
1	0	-6.449743	-3.420070	0.075842
1	0	5.436240	2.123585	0.708179
1	0	5.346882	2.078566	-1.051738
1	0	6.696424	1.285889	-0.217144
1	0	-5.418090	2.172130	-0.704609
1	0	-5.327163	2.125892	1.055169
1	0	-6.684666	1.345449	0.221923
1	0	-0.773409	2.333028	2.672802
1	0	-2.753474	2.416085	0.077289

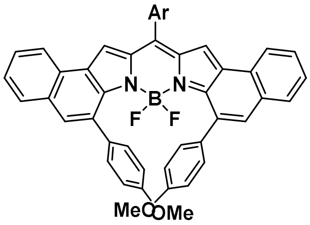

Standard orientation:

Atomic Number	Atomic Type	Coordinates (Angstroms) X	Y	Z
1	0	0.628225	-1.245092	-0.031283
2	0	2.012423	-1.110273	-0.130914
3	0	2.647758	0.137633	-0.123021
4	0	1.888301	1.312992	-0.067096
5	0	0.497974	1.306011	-0.150120
6	0	0.080174	2.601520	-0.006399
7	0	1.230391	3.444447	0.222106
8	0	2.352013	2.627451	0.148779
9	0	0.356092	-2.586373	0.006688
10	0	1.598746	-3.320173	-0.087744
11	0	2.619365	-2.381988	-0.168798
12	0	4.142517	0.215599	-0.139600
13	0	4.817277	0.385593	-1.367210
14	0	4.867637	0.126069	1.067477
15	0	6.213659	0.457795	-1.362632
16	0	6.956555	0.369791	-0.182016
17	0	6.263146	0.209255	1.020571
18	0	4.168174	-0.046465	2.397665
19	0	4.060602	0.488103	-2.672841
20	0	8.466336	0.421883	-0.208446
21	0	-0.361412	-0.023136	-0.248594
22	0	-1.317090	0.010010	0.739281
23	0	-0.885929	-0.151548	-1.529510
24	0	1.647972	-4.758240	-0.066480
25	0	0.409635	-5.439839	0.071114
26	0	-0.798689	-4.677380	0.192831
27	0	-0.884209	-3.300506	0.169133
28	0	2.836727	-5.506512	-0.174304
29	0	2.801913	-6.891875	-0.149438
30	0	1.576307	-7.570551	-0.014524
31	0	0.398284	-6.852359	0.094713
32	0	-1.236585	3.180660	-0.062979
33	0	-1.326120	4.521999	0.245924
34	0	-0.216351	5.377649	0.552971
35	0	1.102548	4.849674	0.507340
36	0	2.194620	5.703810	0.755771
37	0	1.990199	7.043366	1.047884
38	0	0.685773	7.569036	1.097208
39	0	-0.399805	6.746506	0.850549
40	0	3.390431	2.903199	0.266303
41	0	3.684218	-2.555354	-0.232564
42	0	6.733072	0.588297	-2.309940
43	0	6.821518	0.147819	1.952571
44	0	4.888845	-0.025799	3.220466
45	0	3.627015	-0.998381	2.448348
46	0	3.431944	0.746213	2.573112
47	0	3.411969	1.371544	-2.694564
48	0	3.414851	-0.382642	-2.834681
49	0	4.751627	0.557560	-3.518069
50	0	8.870378	0.820739	0.727977
51	0	8.831805	1.047254	-1.029932
52	0	8.895559	-0.579457	-0.347366
53	0	-1.724313	-5.233859	0.315913
54	0	1.556918	-8.656669	0.003647
55	0	-0.552830	-7.369015	0.199900
56	0	-2.310252	4.982004	0.205076
57	0	0.532798	8.619985	1.326352
58	0	-1.410397	7.147060	0.880532
59	0	2.841871	7.690383	1.239766
60	0	3.205198	5.305828	0.721511
61	0	3.787441	-4.991340	-0.281300
62	0	3.726155	-7.456788	-0.235802
63	0	-2.486184	2.504664	-0.515772
64	0	-2.623506	2.074864	-1.846364
65	0	-3.608445	2.443028	0.314885
66	0	-3.838851	1.609569	-2.324873
67	0	-1.769169	2.113114	-2.513484
68	0	-4.837136	1.967802	-0.149467
69	0	-3.522247	2.763972	1.349540
70	0	-4.955816	1.552461	-1.479573
71	0	-3.949643	1.285063	-3.354955
72	0	-5.679581	1.914783	0.530178
73	0	-6.102297	1.069377	-2.047001
74	0	-7.275853	1.018707	-1.246276
75	0	-8.064594	0.641327	-1.900595
76	0	-7.147946	0.337343	-0.397466
77	0	-7.555550	2.017316	-0.883753
78	0	-2.245615	-2.723723	0.394217
79	0	-3.011355	-2.157225	-0.637173

80	0	-2.845413	-2.892069	1.645743
81	0	-4.329670	-1.780530	-0.419905
82	0	-2.565573	-1.996919	-1.612082
83	0	-4.166061	-2.504617	1.884206
84	0	-2.270727	-3.332559	2.456102
85	0	-4.914042	-1.951207	0.842511
86	0	-4.922795	-1.343214	-1.216126
87	0	-4.590066	-2.643354	2.872187
88	0	-6.221647	-1.549492	0.959180
89	0	-6.864362	-1.730288	2.209527
90	0	-6.372545	-1.155591	3.005832
91	0	-7.885275	-1.364950	2.080131
92	0	-6.894173	-2.789273	2.498837

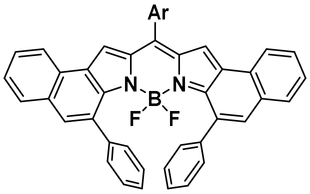

Standard orientation:

Atomic Number	Atomic Type	Coordinates (Angstroms) X	Y	Z
1	0	-1.277959	-0.100632	-0.082726
2	0	-1.262550	1.291621	-0.146161
3	0	-0.073983	2.030154	-0.189114
4	0	1.163461	1.376856	-0.137777
5	0	1.276036	-0.011568	-0.123606
6	0	2.605113	-0.305918	0.020523
7	0	3.350199	0.927820	0.130699
8	0	2.436462	1.966060	0.004653
9	0	-2.586574	-0.482603	0.044807
10	0	-3.420076	0.698730	0.075052
11	0	-2.577351	1.793574	-0.061156
12	0	-0.126267	3.525535	-0.241809
13	0	-0.182941	4.274354	0.952565
14	0	-0.112816	4.176757	-1.493493
15	0	-0.226405	5.670095	0.868524
16	0	-0.219402	6.340003	-0.357752
17	0	-0.159149	5.573789	-1.525225
18	0	-0.047301	3.393486	-2.785676
19	0	-0.191935	3.601070	2.307172
20	0	-0.299670	7.847444	-0.421406
21	0	0.029411	-0.982235	-0.267004
22	0	0.017543	-1.509074	-1.553001
23	0	0.089018	-1.935449	0.721816
24	0	-4.846212	0.630793	0.256132
25	0	-5.411156	-0.662160	0.418318
26	0	-4.555242	-1.811051	0.364272
27	0	-3.189976	-1.783318	0.168634
28	0	-5.689534	1.758751	0.288782
29	0	-7.054647	1.612459	0.478878
30	0	-7.617518	0.332752	0.641543
31	0	-6.805229	-0.787308	0.610494
32	0	3.297640	-1.565957	0.080370
33	0	4.651441	-1.509447	0.339879
34	0	5.416767	-0.307783	0.501445
35	0	4.769197	0.949738	0.369794
36	0	5.528230	2.130254	0.491021
37	0	6.890303	2.069664	0.740343
38	0	7.534600	0.825535	0.874217
39	0	6.806293	-0.345163	0.754378
40	0	2.622586	3.029978	0.042030
41	0	-2.838468	2.842216	-0.070725
42	0	-0.265478	6.247594	1.789986
43	0	-0.146302	6.075125	-2.490994
44	0	-0.063579	4.065247	-3.648911
45	0	-0.891690	2.700983	-2.880742
46	0	0.865465	2.789371	-2.845467
47	0	0.695741	2.974987	2.453273
48	0	-1.063751	2.947632	2.427300
49	0	-0.215762	4.344141	3.109652
50	0	0.240088	8.241580	-1.289100
51	0	0.119674	8.309765	0.478326
52	0	-1.340944	8.186169	-0.506224
53	0	-5.027390	-2.785256	0.462409
54	0	-8.688446	0.226159	0.790004
55	0	-7.232435	-1.779851	0.732539
56	0	5.192332	-2.451050	0.389208
57	0	8.602592	0.786081	1.069492
58	0	7.297427	-1.310488	0.851772
59	0	7.463550	2.988138	0.832860
60	0	5.039359	3.095399	0.390063
61	0	-5.264245	2.750819	0.163871
62	0	-7.693637	2.491052	0.502048
63	0	2.734557	-2.926957	-0.172528
64	0	2.775562	-3.897353	0.838066
65	0	2.341749	-3.309091	-1.462392
66	0	2.440340	-5.223215	0.565620
67	0	3.074371	-3.606286	1.841446
68	0	2.017165	-4.636532	-1.736131
69	0	2.300488	-2.566881	-2.252144
70	0	2.070903	-5.598498	-0.726173
71	0	2.474128	-5.962484	1.361738
72	0	1.728927	-4.920448	-2.744989
73	0	1.822342	-6.633959	-0.944168
74	0	-2.512557	-3.113652	0.091555
75	0	-2.496677	-3.944790	1.220169
76	0	-2.049767	-3.619823	-1.129917
77	0	-2.028034	-5.255213	1.132212
78	0	-2.854430	-3.556941	2.170184
79	0	-1.591169	-4.932788	-1.218518
80	0	-2.049602	-2.983964	-2.008349
81	0	-1.581367	-5.755197	-0.090894
82	0	-2.017685	-5.885192	2.018041
83	0	-1.241491	-5.314535	-2.173720
84	0	-1.223263	-6.778688	-0.164663

Cartesian coordinates of the mechanistic study

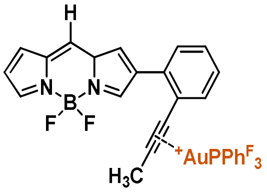

Standard orientation:

Atomic Number	Atomic Type	Coordinates (Angstroms) X	Y	Z
6	0	6.750723	3.670888	-0.475179
6	0	6.213556	3.007154	0.652414
7	0	5.509090	1.931873	0.271847
6	0	6.343008	2.956177	-1.591218
6	0	5.565325	1.860538	-1.124330
6	0	4.962149	0.810513	-1.802678
6	0	4.310989	-0.222806	-1.121797
7	0	4.247615	-0.217766	0.272794
6	0	3.648692	-1.349791	0.660875

6	0	3.281194	-2.131661	-0.465042
6	0	3.701228	-1.406780	-1.589259
5	0	4.650652	0.992892	1.183174
9	0	3.504529	1.662761	1.599776
9	0	5.392446	0.553527	2.266665
6	0	2.628545	-3.446101	-0.390360
6	0	1.571606	-3.834557	-1.262886
6	0	3.029402	-4.364527	0.592683
6	0	2.431986	-5.618661	0.707499
6	0	1.406657	-5.999164	-0.165023
6	0	0.975582	-5.108801	-1.139553
6	0	1.057555	-2.958264	-2.262967
6	0	0.583851	-2.259312	-3.173502
6	0	0.403909	-1.767402	-4.556155
1	0	6.308889	3.265328	1.698924
1	0	6.570201	3.160133	-2.629117
1	0	3.458679	-1.548036	1.706974
1	0	3.632628	-1.704264	-2.626236
1	0	3.841334	-4.095252	1.261088
1	0	2.775312	-6.305810	1.475067
1	0	0.946806	-6.978537	-0.081523
1	0	0.170741	-5.378055	-1.815911
1	0	0.787797	-0.747609	-4.655760
1	0	-0.652699	-1.761469	-4.837623
1	0	0.949228	-2.419593	-5.246229
1	0	7.365178	4.559809	-0.447686
79	0	-0.438325	-1.166636	-1.505400
15	0	-1.583317	0.202106	0.020364
6	0	-1.214692	-0.430685	1.699577
6	0	0.124741	-0.406755	2.109661
6	0	-2.125979	-1.059801	2.554119
6	0	0.549182	-0.960791	3.308241
6	0	-1.723290	-1.618864	3.765419
6	0	-0.384656	-1.571950	4.143269
6	0	-3.392676	0.323433	-0.188894
6	0	-4.155544	1.136863	0.660876
6	0	-4.069494	-0.317037	-1.234785
6	0	-5.521220	1.311401	0.491420
6	0	-5.439427	-0.154934	-1.425356
6	0	-6.164479	0.659996	-0.561138
6	0	-1.014487	1.945760	-0.055635
6	0	-0.751837	2.769564	1.046546
6	0	-0.817763	2.506879	-1.325439
6	0	-0.297074	4.076227	0.891337
6	0	-0.369246	3.809016	-1.503838
6	0	-0.104304	4.596352	-0.385442
9	0	-1.081456	1.782888	-2.426260
9	0	-0.925917	2.331364	2.297137
9	0	-0.051915	4.831718	1.962535
9	0	0.323940	5.845356	-0.538162
9	0	-0.196589	4.304423	-2.730313
9	0	-3.561250	1.759203	1.686945
9	0	-6.216959	2.086163	1.323533
9	0	-7.471682	0.816795	-0.736450
9	0	-6.055134	-0.775802	-2.432309
9	0	-3.426264	-1.107151	-2.103251
9	0	1.041993	0.169525	1.317506
9	0	-3.421377	-1.169368	2.235830
9	0	-2.619246	-2.209381	4.557907
9	0	0.002651	-2.110586	5.295260
9	0	1.839210	-0.923242	3.654323
1	0	5.018097	0.776370	-2.885973

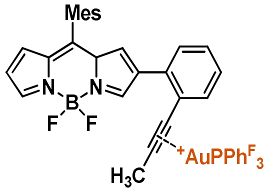

Standard orientation:

Atomic Number	Atomic Type	Coordinates (Angstroms) X	Y	Z
6	0	9.042510	-0.512287	2.592023
6	0	8.257946	-1.687427	2.604110
7	0	7.191428	-1.547352	1.802881
6	0	8.410431	0.380470	1.739429
6	0	7.247084	-0.267971	1.238770
6	0	6.292887	0.159143	0.312121
6	0	5.268265	-0.720726	-0.089305
7	0	5.174102	-2.012480	0.429668
6	0	4.168621	-2.638121	-0.197399
6	0	3.556734	-1.774593	-1.141244
6	0	4.258162	-0.563100	-1.061033
5	0	6.021513	-2.565513	1.622566
9	0	5.242535	-2.601701	2.774652
9	0	6.512974	-3.825418	1.318688
6	0	2.465540	-2.146204	-2.050394
6	0	1.422811	-1.246542	-2.413534
6	0	2.443616	-3.430571	-2.620258
6	0	1.449804	-3.816172	-3.516615
6	0	0.445452	-2.915551	-3.890501
6	0	0.436210	-1.638998	-3.344633
6	0	1.323146	0.049473	-1.821388
6	0	1.267819	1.169763	-1.288231
6	0	1.688722	2.547652	-0.958860
1	0	8.422079	-2.606280	3.151545
1	0	8.726359	1.380255	1.475074
1	0	3.902299	-3.655977	0.055994
1	0	4.119367	0.317700	-1.671142
1	0	3.238970	-4.126459	-2.371259
1	0	1.468176	-4.816948	-3.938149
1	0	-0.320997	-3.208575	-4.601005
1	0	-0.341870	-0.929978	-3.610424
1	0	1.601226	2.744343	0.112989
1	0	1.067150	3.275015	-1.490858
1	0	2.733043	2.688867	-1.256785
1	0	9.960257	-0.362009	3.143241
79	0	-0.779731	0.505127	-0.664832
15	0	-2.964410	0.168368	0.118404
6	0	-3.533028	-1.479282	-0.444021
6	0	-2.798559	-2.591059	-0.010121
6	0	-4.566988	-1.721399	-1.355270
6	0	-3.077199	-3.883120	-0.431522
6	0	-4.864154	-3.009733	-1.794618
6	0	-4.122004	-4.091750	-1.331147
6	0	-4.185059	1.426197	-0.392820
6	0	-5.500421	1.377359	0.090946
6	0	-3.849110	2.508617	-1.216250
6	0	-6.440188	2.350431	-0.216470
6	0	-4.776020	3.497155	-1.537964
6	0	-6.071748	3.417477	-1.036291
6	0	-3.048255	0.198136	1.950947
6	0	-3.759282	-0.699211	2.757658
6	0	-2.328483	1.206342	2.607119
6	0	-3.732043	-0.611622	4.147314
6	0	-2.291552	1.316563	3.990926
6	0	-2.996023	0.396320	4.764680

9	0	-1.654970	2.120161	1.888367
9	0	-4.495747	-1.678525	2.223343
9	0	-4.415599	-1.485972	4.886870
9	0	-2.973002	0.487015	6.090578
9	0	-1.595441	2.292505	4.575806
9	0	-5.883155	0.349255	0.859320
9	0	-7.684138	2.268746	0.255079
9	0	-6.960749	4.355583	-1.341497
9	0	-4.424992	4.516334	-2.323243
9	0	-2.619549	2.642620	-1.729397
9	0	-1.783133	-2.409201	0.849987
9	0	-5.304357	-0.724598	-1.859424
9	0	-5.857644	-3.205338	-2.662888
9	0	-4.406080	-5.322103	-1.747137
9	0	-2.357144	-4.913677	0.013278
6	0	6.357820	1.530263	-0.280836
6	0	7.043026	1.737065	-1.498760
6	0	5.710077	2.599912	0.370734
6	0	7.061369	3.023357	-2.045725
6	0	5.756205	3.870013	-0.218497
6	0	6.424062	4.103994	-1.424186
1	0	7.588048	3.184193	-2.984221
1	0	5.257869	4.696689	0.283126
6	0	6.469923	5.483460	-2.040048
1	0	7.491311	5.884667	-2.037251
1	0	6.136930	5.464778	-3.084811
1	0	5.833614	6.186524	-1.493630
6	0	7.749588	0.604231	-2.210197
1	0	8.522806	0.151923	-1.578256
1	0	7.054482	-0.197054	-2.486706
1	0	8.229969	0.962131	-3.125106
6	0	4.973628	2.400474	1.677422
1	0	5.642072	2.035184	2.465207
1	0	4.532739	3.340335	2.021075
1	0	4.166399	1.664730	1.578581

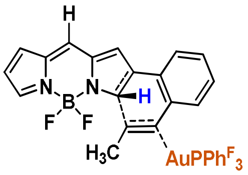

Standard orientation:

Atomic Number	Atomic Type	Coordinates (Angstroms) X	Y	Z
6	0	-9.216490	-2.852861	0.941700
6	0	-8.315732	-2.730429	-0.161012
7	0	-7.602589	-1.612591	-0.066015
6	0	-9.030727	-1.743782	1.730286
6	0	-8.005905	-0.951092	1.107573
6	0	-7.411920	0.221718	1.489216
6	0	-6.350525	0.790849	0.728717
7	0	-5.880769	0.162946	-0.398432
6	0	-4.835917	0.904558	-0.888641
6	0	-4.696978	2.086043	-0.085196
6	0	-5.627835	1.981554	0.949241
5	0	-6.588747	-1.034333	-1.120854
9	0	-7.277546	-0.567792	-2.227217
9	0	-5.673360	-2.012069	-1.476146
6	0	-3.683314	3.107064	-0.357087
6	0	-2.347003	2.672628	-0.562915
6	0	-3.998258	4.465020	-0.441055
6	0	-3.004347	5.402600	-0.738027
6	0	-1.692565	4.981009	-0.954270
6	0	-1.366169	3.625573	-0.862301
6	0	-2.063285	1.244096	-0.368075
6	0	-2.942023	0.297134	-0.198816
6	0	-3.174706	-1.096050	0.227046
1	0	-8.181592	-3.408582	-0.994069
1	0	-9.531024	-1.489613	2.654892
1	0	-4.540590	0.781207	-1.922861
1	0	-5.764962	2.661970	1.778282
1	0	-5.024717	4.786191	-0.291748
1	0	-3.258884	6.456321	-0.799566
1	0	-0.917258	5.705589	-1.184670
1	0	-0.339190	3.305630	-1.010075
1	0	-2.216026	-1.530206	0.531328
1	0	-3.617847	-1.703320	-0.565362
1	0	-3.845910	-1.129040	1.093759
1	0	-9.899052	-3.675477	1.100199
79	0	-0.098614	0.508236	-0.258640
15	0	2.154115	-0.201527	-0.042945
6	0	3.192618	1.283567	0.243968
6	0	2.913047	2.047949	1.384058
6	0	4.149104	1.796292	-0.637931
6	0	3.557106	3.245536	1.659762
6	0	4.807663	2.998096	-0.384812
6	0	4.513642	3.723012	0.765477
6	0	2.884616	-1.162355	-1.417076
6	0	4.186220	-1.675078	-1.331333
6	0	2.146885	-1.484996	-2.562623
6	0	4.736615	-2.470579	-2.325769
6	0	2.677877	-2.283084	-3.573261
6	0	3.973408	-2.776139	-3.452782
6	0	2.379732	-1.316399	1.404160
6	0	3.406468	-1.243827	2.353343
6	0	1.425136	-2.327102	1.586015
6	0	3.464281	-2.113306	3.439458
6	0	1.465432	-3.209365	2.657981
6	0	2.492075	-3.097418	3.592692
9	0	0.432102	-2.480577	0.692596
9	0	4.378712	-0.330421	2.256608
9	0	4.454106	-2.010332	4.328565
9	0	2.547829	-3.934973	4.624415
9	0	0.536648	-4.158345	2.790843
9	0	4.945175	-1.379650	-0.267422
9	0	5.981831	-2.935828	-2.215722
9	0	4.487327	-3.536759	-4.413973
9	0	1.949151	-2.576074	-4.651766
9	0	0.894669	-1.043442	-2.732113
9	0	1.987147	1.611849	2.254974
9	0	4.460833	1.164337	-1.776795
9	0	5.716432	3.456075	-1.248371
9	0	5.140909	4.870474	1.010571
9	0	3.264714	3.936616	2.763075
1	0	-7.746345	0.730338	2.386663

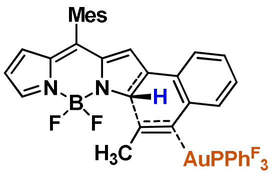

Standard orientation:

Atomic Number	Atomic Type	Coordinates (Angstroms) X	Y	Z
6	0	-7.862602	-3.924682	0.480202
6	0	-6.772669	-4.039868	-0.432848
7	0	-6.227127	-2.849494	-0.667443
6	0	-7.972208	-2.592220	0.800020

6	0	-6.937950	-1.897295	0.083850
6	0	-6.579833	-0.565807	0.088609
6	0	-5.424109	-0.151035	-0.659910
7	0	-4.664835	-1.051336	-1.366229
6	0	-3.616231	-0.370961	-1.931078
6	0	-3.761538	1.023407	-1.630544
6	0	-4.879967	1.142474	-0.803846
5	0	-5.091859	-2.518961	-1.696375
9	0	-5.589862	-2.579739	-2.987900
9	0	-4.032135	-3.397036	-1.528696
6	0	-2.814511	2.037426	-2.097085
6	0	-1.428615	1.789308	-1.909722
6	0	-3.234609	3.212311	-2.725084
6	0	-2.297414	4.144098	-3.181115
6	0	-0.934811	3.900976	-3.009515
6	0	-0.505094	2.733524	-2.373878
6	0	-1.047922	0.582208	-1.163772
6	0	-1.837834	-0.381349	-0.785981
6	0	-2.010025	-1.568511	0.071794
1	0	-6.388021	-4.931811	-0.910601
1	0	-8.677525	-2.124636	1.472493
1	0	-3.100658	-0.806595	-2.777742
1	0	-5.254178	2.042059	-0.336575
1	0	-4.295769	3.388693	-2.872848
1	0	-2.635232	5.054316	-3.667252
1	0	-0.202835	4.622741	-3.359447
1	0	0.555640	2.559122	-2.221527
1	0	-1.095568	-1.709355	0.658439
1	0	-2.218129	-2.471491	-0.506804
1	0	-2.837542	-1.420428	0.775741
1	0	-8.465057	-4.746293	0.840390
79	0	0.934709	0.231063	-0.558497
15	0	3.189766	-0.070940	0.113998
6	0	4.110755	1.478838	-0.226401
6	0	3.665012	2.639303	0.418989
6	0	5.133003	1.635950	-1.167890
6	0	4.207535	3.891642	0.169338
6	0	5.692289	2.883488	-1.438325
6	0	5.231517	4.012282	-0.768506
6	0	4.111964	-1.462434	-0.634913
6	0	5.413124	-1.776001	-0.218228
6	0	3.533366	-2.299224	-1.597296
6	0	6.109551	-2.866903	-0.718081
6	0	4.213041	-3.400203	-2.113144
6	0	5.501538	-3.683811	-1.671219
6	0	3.323089	-0.404964	1.918887
6	0	4.234963	0.182075	2.804493
6	0	2.413840	-1.320356	2.467406
6	0	4.224886	-0.106632	4.166588
6	0	2.388540	-1.628492	3.821604
6	0	3.299277	-1.012323	4.676658
9	0	1.535557	-1.953259	1.670318
9	0	5.158473	1.049626	2.376395
9	0	5.104977	0.477364	4.982212
9	0	3.290602	-1.296479	5.975954
9	0	1.507419	-2.508903	4.300678
9	0	6.028959	-0.992645	0.677458
9	0	7.349115	-3.132519	-0.303174
9	0	6.156964	-4.731468	-2.160402
9	0	3.633459	-4.180042	-3.027487
9	0	2.298582	-2.076493	-2.063564
9	0	2.674099	2.546968	1.321975
9	0	5.607797	0.597058	-1.866780
9	0	6.667111	2.996013	-2.342990
9	0	5.764284	5.204391	-1.023757
9	0	3.756174	4.969274	0.813779
6	0	-7.371043	0.446857	0.848744
6	0	-8.502065	1.040024	0.243194
6	0	-6.977484	0.807108	2.154093
6	0	-9.219346	1.995756	0.966907
6	0	-7.734125	1.765991	2.837973
6	0	-8.854645	2.373156	2.265076
1	0	-10.087021	2.458364	0.501544
1	0	-7.435975	2.042685	3.846520
6	0	-5.780177	0.171432	2.825322
1	0	-5.895566	-0.915531	2.912294
1	0	-4.855041	0.352774	2.264904
1	0	-5.642956	0.574031	3.832540
6	0	-8.939971	0.666581	-1.156125
1	0	-8.128856	0.788931	-1.883689
1	0	-9.266542	-0.378575	-1.213429
1	0	-9.775453	1.294065	-1.478113
6	0	-9.656119	3.408005	3.019302
1	0	-9.688015	4.359216	2.474314
1	0	-10.694715	3.082544	3.155402
1	0	-9.230854	3.599972	4.008874

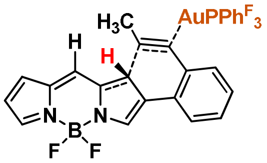

Standard orientation:

Atomic Number	Atomic Type	Coordinates (Angstroms) X	Y	Z
6	0	-9.389388	2.989789	-0.722579
6	0	-9.279420	2.020959	0.313097
7	0	-8.180102	1.280854	0.150206
6	0	-8.295153	2.819568	-1.544083
6	0	-7.527074	1.740625	-1.001769
6	0	-6.372187	1.134351	-1.445831
6	0	-5.825833	0.017153	-0.780945
7	0	-6.433584	-0.497206	0.337748
6	0	-5.799919	-1.638222	0.701888
6	0	-4.746087	-1.900687	-0.179949
6	0	-4.713015	-0.817605	-1.113714
5	0	-7.623679	0.183575	1.120420
9	0	-8.592861	-0.758467	1.397268
9	0	-7.132598	0.774289	2.271874
6	0	-3.746411	-2.959982	-0.113213
6	0	-2.374607	-2.596262	-0.218165
6	0	-4.097274	-4.302234	0.068902
6	0	-3.114969	-5.292954	0.126880
6	0	-1.769560	-4.943740	0.008223
6	0	-1.405026	-3.605739	-0.155696
6	0	-2.028171	-1.171477	-0.302814
6	0	-2.797681	-0.141870	-0.468958
6	0	-2.926761	1.327313	-0.541092
1	0	-9.948349	1.847968	1.146144
1	0	-8.042844	3.369700	-2.440416
1	0	-6.107378	-2.191575	1.578094
1	0	-4.294688	-0.864289	-2.112587
1	0	-5.146938	-4.571192	0.139793
1	0	-3.402925	-6.330911	0.263054
1	0	-0.999927	-5.708240	0.054323
1	0	-0.354751	-3.338683	-0.222919
1	0	-3.219313	1.661888	-1.542190
1	0	-1.958097	1.776692	-0.296723
1	0	-3.661796	1.697871	0.180244
1	0	-10.192415	3.705472	-0.826511
79	0	-0.031384	-0.515941	-0.070307
15	0	2.232876	0.159016	0.078639

6	0	3.229661	-0.937835	-1.002327
6	0	2.925773	-0.936528	-2.369523
6	0	4.175244	-1.872630	-0.568457
6	0	3.535885	-1.794008	-3.273663
6	0	4.799177	-2.746972	-1.456350
6	0	4.481644	-2.707102	-2.810187
6	0	2.998849	0.174382	1.739050
6	0	4.297523	0.664215	1.935498
6	0	2.299048	-0.245491	2.877240
6	0	4.876204	0.748684	3.193680
6	0	2.859316	-0.169979	4.150156
6	0	4.148679	0.329074	4.307207
6	0	2.467520	1.882941	-0.521721
6	0	3.498668	2.328355	-1.358683
6	0	1.521729	2.840306	-0.126986
6	0	3.573170	3.648524	-1.794547
6	0	1.578663	4.163811	-0.545070
6	0	2.611388	4.568514	-1.387435
9	0	0.517305	2.496790	0.698524
9	0	4.459740	1.498386	-1.777725
9	0	4.567842	4.034570	-2.595711
9	0	2.681761	5.832010	-1.795889
9	0	0.657224	5.041200	-0.142806
9	0	5.027828	1.049143	0.880530
9	0	6.116290	1.216370	3.342753
9	0	4.690873	0.401593	5.518280
9	0	2.165673	-0.575902	5.214900
9	0	1.058197	-0.739977	2.789706
9	0	2.009421	-0.071262	-2.835666
9	0	4.509319	-1.982441	0.723628
9	0	5.698406	-3.624951	-1.007369
9	0	5.076833	-3.539228	-3.660513
9	0	3.221808	-1.751462	-4.569667
1	0	-5.885364	1.506571	-2.340880

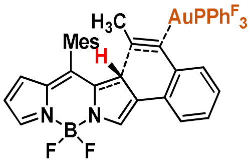

Standard orientation:

Atomic Number	Atomic Type	Coordinates (Angstroms) X	Y	Z
6	0	9.134509	0.570071	-2.023546
6	0	8.643756	-0.743235	-2.250409
7	0	7.473581	-0.919282	-1.630956
6	0	8.207287	1.207881	-1.225889
6	0	7.150167	0.275161	-0.974050
6	0	6.013048	0.394039	-0.184166
6	0	5.185518	-0.751533	0.024151
7	0	5.516321	-1.964367	-0.525925
6	0	4.728817	-2.933060	0.001050
6	0	3.841378	-2.371947	0.920721
6	0	4.060845	-0.954459	0.894175
5	0	6.557693	-2.178828	-1.687814
9	0	7.285175	-3.327665	-1.454261
9	0	5.881429	-2.229859	-2.895902
6	0	2.785721	-3.024297	1.685639
6	0	1.485424	-2.451829	1.649385
6	0	3.018172	-4.181049	2.437402
6	0	1.986242	-4.766960	3.173749
6	0	0.712354	-4.198210	3.157529
6	0	0.464201	-3.054638	2.395015
6	0	1.257973	-1.305687	0.760140
6	0	2.136361	-0.588921	0.123624
6	0	2.352694	0.495791	-0.854156
1	0	9.095071	-1.543965	-2.821772
1	0	8.253491	2.213088	-0.831315
1	0	4.822715	-3.961493	-0.317975
1	0	3.823155	-0.275351	1.703647
1	0	4.017023	-4.606072	2.464777
1	0	2.180549	-5.661723	3.757273
1	0	-0.094492	-4.648808	3.727594
1	0	-0.535751	-2.632225	2.368886
1	0	2.861307	1.352839	-0.402489
1	0	1.379455	0.829456	-1.230332
1	0	2.941414	0.145960	-1.708336
1	0	10.067454	0.964557	-2.400080
79	0	-0.696626	-0.689832	0.264993
15	0	-2.910905	0.026652	-0.183566
6	0	-3.883346	-0.082419	1.368635
6	0	-3.411742	0.651072	2.465171
6	0	-4.983053	-0.916142	1.595642
6	0	-3.998912	0.584309	3.720430
6	0	-5.588344	-1.002823	2.847993
6	0	-5.098143	-0.251300	3.911130
6	0	-3.824902	-0.849231	-1.503256
6	0	-5.080552	-0.403451	-1.939111
6	0	-3.276430	-1.953402	-2.168149
6	0	-5.758799	-1.007254	-2.988043
6	0	-3.938923	-2.575558	-3.223457
6	0	-5.180211	-2.099578	-3.634151
6	0	-2.950004	1.786637	-0.720379
6	0	-3.847288	2.763605	-0.271070
6	0	-1.978008	2.202813	-1.641100
6	0	-3.765591	4.087041	-0.695939
6	0	-1.879282	3.516137	-2.082403
6	0	-2.779001	4.464870	-1.602109
9	0	-1.103820	1.313329	-2.143875
9	0	-4.825421	2.464121	0.590191
9	0	-4.635197	4.991554	-0.241986
9	0	-2.700325	5.726643	-2.014732
9	0	-0.937898	3.867348	-2.960619
9	0	-5.671847	0.625853	-1.318381
9	0	-6.954594	-0.559417	-3.373411
9	0	-5.819248	-2.689168	-4.639195
9	0	-3.388542	-3.623920	-3.838105
9	0	-2.088563	-2.460037	-1.815636
9	0	-2.352358	1.462371	2.303739
9	0	-5.493803	-1.682543	0.623749
9	0	-6.636539	-1.809032	3.028209
9	0	-5.675453	-0.330201	5.107109
9	0	-3.520561	1.307641	4.734436
6	0	5.702173	1.689599	0.489808
6	0	5.927367	1.823183	1.882571
6	0	5.220419	2.782712	-0.265524
6	0	5.619239	3.039878	2.495220
6	0	4.909996	3.973269	0.403828
6	0	5.092451	4.122377	1.780500
1	0	5.806902	3.148388	3.561077
1	0	4.523140	4.808996	-0.174846
6	0	5.052005	2.730522	-1.770584
1	0	4.274704	3.429377	-2.093929
1	0	5.979798	3.019851	-2.279648
1	0	4.787174	1.735367	-2.136461
6	0	6.552128	0.717243	2.707851
1	0	5.861536	-0.113946	2.890045
1	0	7.436648	0.296944	2.216401
1	0	6.862154	1.101903	3.683306
6	0	4.730126	5.408844	2.483188
1	0	3.750261	5.325681	2.972799
1	0	5.459205	5.656688	3.261580

1	0	4.677360	6.247549	1.781789

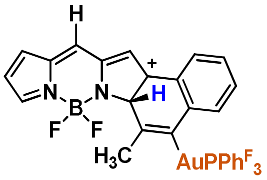

Standard orientation:

Atomic Number	Atomic Type	Coordinates (Angstroms) X	Y	Z
6	0	9.732841	-2.369127	0.097391
6	0	8.367464	-2.574828	0.513229
7	0	7.619261	-1.529907	0.211017
6	0	9.786808	-1.139026	-0.490235
6	0	8.450496	-0.581731	-0.420609
6	0	7.951420	0.624114	-0.782603
6	0	6.578368	0.975494	-0.479314
7	0	5.700900	0.087498	0.007515
6	0	4.501614	0.841718	0.416185
6	0	4.764045	2.232011	-0.059946
6	0	6.046403	2.286666	-0.559924
5	0	6.045457	-1.405978	0.336162
9	0	5.666084	-1.713441	1.627997
9	0	5.493230	-2.264657	-0.599153
6	0	3.724380	3.210123	-0.003366
6	0	2.381134	2.726783	0.105430
6	0	3.996516	4.590081	-0.119011
6	0	2.964824	5.512029	-0.096891
6	0	1.640668	5.056887	0.021777
6	0	1.359231	3.699249	0.113724
6	0	2.074914	1.287502	0.150009
6	0	3.064856	0.351976	0.240532
6	0	2.834330	-1.126139	0.372121
1	0	7.946568	-3.443162	1.004861
1	0	10.642612	-0.635135	-0.918033
1	0	4.597664	0.890998	1.522137
1	0	6.570234	3.139733	-0.968119
1	0	5.026925	4.924457	-0.195081
1	0	3.176119	6.574296	-0.166813
1	0	0.824339	5.773368	0.036624
1	0	0.326248	3.374958	0.187094
1	0	3.310023	-1.671852	-0.449449
1	0	1.767321	-1.356392	0.380210
1	0	3.282602	-1.513585	1.294738
1	0	10.537009	-3.075679	0.244644
79	0	0.091277	0.692093	0.050158
15	0	-2.158700	-0.141734	-0.071980
6	0	-2.145428	-1.619612	-1.166786
6	0	-1.316319	-2.683475	-0.788968
6	0	-2.773497	-1.728178	-2.411173
6	0	-1.126255	-3.806739	-1.581018
6	0	-2.597217	-2.844467	-3.227053
6	0	-1.774677	-3.886286	-2.811985
6	0	-3.489808	0.976508	-0.653057
6	0	-4.834793	0.583115	-0.654705
6	0	-3.213701	2.290287	-1.050001
6	0	-5.860218	1.444519	-1.018507
6	0	-4.225398	3.173457	-1.419594
6	0	-5.550214	2.749287	-1.401180
6	0	-2.788258	-0.703986	1.567100
6	0	-3.459616	-1.902554	1.836275
6	0	-2.548070	0.139555	2.660509
6	0	-3.848543	-2.253663	3.126372
6	0	-2.932033	-0.185914	3.955537
6	0	-3.583328	-1.394552	4.188612
9	0	-1.940426	1.322526	2.472527
9	0	-3.758218	-2.767102	0.859576
9	0	-4.482238	-3.408460	3.345386
9	0	-3.959608	-1.722497	5.422313
9	0	-2.685646	0.649983	4.966960
9	0	-5.160775	-0.671764	-0.316623
9	0	-7.129966	1.034049	-1.012696
9	0	-6.522153	3.585555	-1.753792
9	0	-3.927858	4.420709	-1.790741
9	0	-1.958143	2.754842	-1.094095
9	0	-0.676030	-2.629521	0.392921
9	0	-3.561811	-0.754155	-2.885832
9	0	-3.215130	-2.912264	-4.408896
9	0	-1.605089	-4.954769	-3.587864
9	0	-0.332637	-4.800031	-1.174250
1	0	8.583649	1.367941	-1.253088

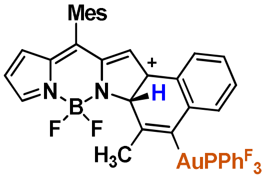

Standard orientation:

Atomic Number	Atomic Type	Coordinates (Angstroms) X	Y	Z
6	0	8.088686	-3.959660	0.553238
6	0	6.683459	-4.002217	0.857714
7	0	6.124928	-2.818694	0.672821
6	0	8.367161	-2.684204	0.151869
6	0	7.130544	-1.934619	0.233015
6	0	6.862674	-0.616220	0.007142
6	0	5.506359	-0.125248	0.284372
7	0	4.476449	-0.937036	0.555177
6	0	3.356615	-0.091619	1.009417
6	0	3.847800	1.301634	0.807049
6	0	5.163439	1.246704	0.405779
6	0	7.913104	0.338266	-0.445269
6	0	8.864488	0.833806	0.474147
6	0	7.939400	0.736597	-1.801597
6	0	9.833875	1.727837	0.007691
6	0	9.889988	2.134785	-1.328947
6	0	8.935873	1.623608	-2.215997
6	0	8.849844	0.444075	1.936842
6	0	6.940287	0.200078	-2.802534
6	0	10.933615	3.120584	-1.797284
5	0	4.582627	-2.493333	0.681045
9	0	4.028081	-2.914783	1.874879
9	0	4.021697	-3.126664	-0.416220
6	0	2.949724	2.402163	0.967521
6	0	1.550162	2.115295	0.884505
6	0	3.415788	3.723800	1.130474
6	0	2.520652	4.773462	1.245563
6	0	1.142631	4.509906	1.175928
6	0	0.673128	3.214401	0.993111
6	0	1.050776	0.750787	0.641830
6	0	1.891003	-0.323949	0.637912
6	0	1.453561	-1.752340	0.480911
1	0	6.097630	-4.852301	1.184310
1	0	9.316592	-2.272391	-0.159305
1	0	3.351459	-0.247709	2.108858
1	0	5.830641	2.068473	0.190635
1	0	10.563428	2.118623	0.713137
1	0	8.967058	1.920651	-3.261656

1	0	9.176839	-0.591270	2.089994
1	0	7.849329	0.533659	2.374996
1	0	9.523488	1.087411	2.509171
1	0	6.980023	-0.893767	-2.869161
1	0	7.138809	0.602775	-3.799310
1	0	5.910345	0.469107	-2.537292
1	0	11.828210	3.085199	-1.167661
1	0	10.546632	4.147441	-1.759068
1	0	11.231998	2.925160	-2.832430
1	0	4.484273	3.906640	1.196258
1	0	2.879433	5.787529	1.390612
1	0	0.432845	5.327867	1.260569
1	0	-0.396188	3.041915	0.925078
1	0	1.940780	-2.219882	-0.381131
1	0	0.371064	-1.818643	0.356210
1	0	1.741306	-2.349478	1.354467
1	0	8.766797	-4.796970	0.635209
79	0	-0.974308	0.486568	0.283675
15	0	-3.292770	0.028454	-0.146013
6	0	-3.369002	-1.275879	-1.439883
6	0	-2.745677	-2.496188	-1.150256
6	0	-3.863792	-1.118919	-2.737968
6	0	-2.630543	-3.520591	-2.078895
6	0	-3.759267	-2.131419	-3.690125
6	0	-3.144125	-3.334552	-3.361056
6	0	-4.390517	1.400550	-0.668291
6	0	-5.767353	1.214955	-0.851372
6	0	-3.899032	2.700866	-0.833388
6	0	-6.621350	2.260398	-1.173275
6	0	-4.735914	3.766428	-1.156432
6	0	-6.099043	3.544725	-1.324351
6	0	-4.165573	-0.640791	1.333494
6	0	-5.018863	-1.749879	1.368816
6	0	-3.935766	0.013512	2.551574
6	0	-5.592212	-2.198364	2.555875
6	0	-4.501491	-0.410979	3.747482
6	0	-5.333016	-1.528128	3.747748
9	0	-3.155969	1.106639	2.586975
9	0	-5.322117	-2.429937	0.256875
9	0	-6.397085	-3.263886	2.551895
9	0	-5.884987	-1.948559	4.883485
9	0	-4.257687	0.244871	4.884541
9	0	-6.293572	-0.012111	-0.737337
9	0	-7.927125	2.045751	-1.345975
9	0	-6.905019	4.556056	-1.634316
9	0	-4.235068	4.994909	-1.304815
9	0	-2.596346	2.975960	-0.687710
9	0	-2.237085	-2.698226	0.078779
9	0	-4.445740	0.021649	-3.132985
9	0	-4.245373	-1.944701	-4.919802
9	0	-3.042945	-4.304305	-4.267616
9	0	-2.035470	-4.670404	-1.753813

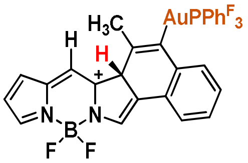

Standard orientation:

Atomic Number	Atomic Type	Coordinates (Angstroms) X	Y	Z
6	0	8.930178	-3.413784	0.536918
6	0	9.218272	-2.034084	0.731377
7	0	8.176540	-1.280999	0.362780
6	0	7.654309	-3.485607	0.021316
6	0	7.169484	-2.141482	-0.088422
6	0	5.944603	-1.653494	-0.493645
6	0	5.637286	-0.279508	-0.407291
7	0	6.577396	0.611390	-0.030644
6	0	6.091870	1.886839	-0.023814
6	0	4.766378	1.892994	-0.421710
6	0	4.406169	0.495265	-0.790763
5	0	8.106935	0.272744	0.298027
9	0	8.878075	0.759061	-0.736500
9	0	8.427560	0.840093	1.509395
6	0	3.784291	2.919684	-0.441117
6	0	2.407846	2.506704	-0.489841
6	0	4.130198	4.289129	-0.358292
6	0	3.147984	5.259923	-0.354087
6	0	1.795553	4.874145	-0.418729
6	0	1.439423	3.534232	-0.478646
6	0	2.016547	1.090807	-0.490941
6	0	2.956249	0.102593	-0.591403
6	0	2.661971	-1.368112	-0.644398
1	0	10.125439	-1.582938	1.111960
1	0	7.091414	-4.369606	-0.245343
1	0	6.723873	2.701110	0.299781
1	0	4.500138	0.486656	-1.905304
1	0	5.177937	4.571994	-0.326050
1	0	3.415587	6.310618	-0.305941
1	0	1.019283	5.633723	-0.415866
1	0	0.389028	3.265491	-0.515698
1	0	3.019938	-1.816588	-1.581967
1	0	1.591213	-1.565145	-0.565496
1	0	3.167045	-1.895565	0.178204
1	0	9.601456	-4.229466	0.763968
79	0	0.013866	0.599077	-0.284171
15	0	-2.265115	-0.120865	-0.030391
6	0	-2.546828	-1.533860	-1.172968
6	0	-1.716153	-2.651297	-1.022295
6	0	-3.420929	-1.547447	-2.264383
6	0	-1.750554	-3.735930	-1.886589
6	0	-3.473715	-2.623755	-3.148489
6	0	-2.639440	-3.720316	-2.959783
6	0	-3.615050	1.089417	-0.295456
6	0	-4.951767	0.776880	-0.013562
6	0	-3.347019	2.393803	-0.726775
6	0	-5.972834	1.709460	-0.131616
6	0	-4.354001	3.347501	-0.852711
6	0	-5.668248	3.003852	-0.551829
6	0	-2.597134	-0.740208	1.673153
6	0	-3.261295	-1.924233	2.014583
6	0	-2.112117	0.036148	2.734547
6	0	-3.411242	-2.326941	3.338949
6	0	-2.255741	-0.340945	4.064049
6	0	-2.906827	-1.534458	4.365874
9	0	-1.495396	1.202615	2.483242
9	0	-3.784286	-2.722666	1.076756
9	0	-4.045627	-3.466072	3.626517
9	0	-3.054413	-1.911584	5.633437
9	0	-1.780196	0.430924	5.043806
9	0	-5.279775	-0.466390	0.362848
9	0	-7.235294	1.375774	0.142315
9	0	-6.636617	3.906884	-0.672274
9	0	-4.063358	4.583673	-1.263482
9	0	-2.103842	2.778416	-1.045560
9	0	-0.847101	-2.690874	0.004377
9	0	-4.237242	-0.516977	-2.522173
9	0	-4.322191	-2.600460	-4.178964
9	0	-2.687264	-4.750451	-3.801396
9	0	-0.943834	-4.782656	-1.697321
1	0	5.197602	-2.348146	-0.854430

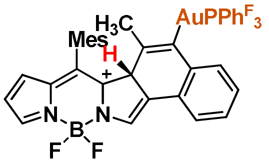

Standard orientation:

Atomic Number	Atomic Type	Coordinates (Angstroms) X	Y	Z
6	0	-9.212139	1.402304	0.577601
6	0	-9.131031	0.009036	0.840250
7	0	-7.873305	-0.420372	0.691340
6	0	-7.939593	1.819159	0.252014
6	0	-7.081276	0.670757	0.324249
6	0	-5.725797	0.555601	0.024110
6	0	-5.104229	-0.738175	0.048770
7	0	-5.863717	-1.827798	0.300720
6	0	-5.211763	-2.992790	0.033541
6	0	-3.933181	-2.729834	-0.411808
6	0	-3.763164	-1.245838	-0.459221
6	0	-5.002753	1.805092	-0.362576
6	0	-4.693400	2.062932	-1.714292
6	0	-4.670117	2.746861	0.643521
6	0	-4.001001	3.239579	-2.031098
6	0	-3.611819	4.158130	-1.054409
6	0	-3.972065	3.897227	0.274590
6	0	-5.091257	1.133063	-2.841992
6	0	-5.017416	2.530597	2.101980
6	0	-2.812333	5.389759	-1.407165
5	0	-7.329518	-1.843304	0.935349
9	0	-8.085375	-2.793956	0.287886
9	0	-7.189272	-2.108258	2.282539
6	0	-2.861856	-3.602472	-0.744351
6	0	-1.545295	-3.034654	-0.778137
6	0	-3.067438	-4.978137	-1.003901
6	0	-2.002895	-5.791042	-1.342265
6	0	-0.708288	-5.241824	-1.411179
6	0	-0.487588	-3.901100	-1.126991
6	0	-1.305068	-1.647154	-0.371161
6	0	-2.339154	-0.785849	-0.123911
6	0	-2.152516	0.619571	0.358737
1	0	-9.923144	-0.675599	1.113992
1	0	-7.620428	2.812282	-0.029279
1	0	-5.709480	-3.936677	0.201324
1	0	-3.831277	-1.001111	-1.543563
1	0	-3.765494	3.439394	-3.074142
1	0	-3.694225	4.609026	1.048613
1	0	-5.938594	0.492133	-2.583254
1	0	-4.258071	0.485653	-3.147589
1	0	-5.372917	1.711208	-3.727670
1	0	-6.080551	2.713391	2.297672
1	0	-4.445385	3.214935	2.734376
1	0	-4.805304	1.508533	2.435278
1	0	-2.811467	5.571895	-2.486033
1	0	-1.769014	5.279883	-1.084768
1	0	-3.210650	6.280552	-0.908600
1	0	-4.074247	-5.383290	-0.970845
1	0	-2.163564	-6.842086	-1.559926
1	0	0.131019	-5.876428	-1.680474
1	0	0.523124	-3.507576	-1.164698
1	0	-2.415496	1.358609	-0.405170
1	0	-1.115131	0.791965	0.650786
1	0	-2.792375	0.814014	1.227273
1	0	-10.114775	1.995051	0.613742
79	0	0.647840	-0.980688	-0.163741
15	0	2.816048	0.032708	0.041790
6	0	2.649962	1.739496	-0.624756
6	0	1.703928	2.571876	-0.012976
6	0	3.255625	2.229490	-1.785602
6	0	1.369877	3.822396	-0.511820
6	0	2.939562	3.484044	-2.305270
6	0	1.993521	4.281403	-1.670171
6	0	4.257997	-0.753002	-0.769892
6	0	5.555435	-0.245785	-0.618702
6	0	4.120494	-1.933982	-1.508629
6	0	6.666977	-0.878422	-1.157414
6	0	5.220478	-2.587564	-2.058769
6	0	6.495033	-2.059205	-1.879619
6	0	3.369645	0.190508	1.790832
6	0	3.904127	1.333040	2.397183
6	0	3.216448	-0.940860	2.603571
6	0	4.245623	1.355709	3.746797
6	0	3.556226	-0.944639	3.950744
6	0	4.070260	0.215333	4.525360
9	0	2.740945	-2.081358	2.077198
9	0	4.110437	2.456740	1.700570
9	0	4.747583	2.465458	4.293908
9	0	4.401696	0.230401	5.814069
9	0	3.396879	-2.045524	4.688599
9	0	5.746688	0.899526	0.049724
9	0	7.888267	-0.365489	-0.997137
9	0	7.550539	-2.675884	-2.402701
9	0	5.055456	-3.713928	-2.755584
9	0	2.918363	-2.485758	-1.720199
9	0	1.084893	2.151231	1.104796
9	0	4.153041	1.504492	-2.466259
9	0	3.538862	3.916568	-3.417030
9	0	1.682141	5.477007	-2.165592
9	0	0.454787	4.579607	0.102525

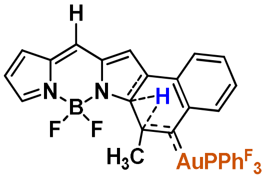

Standard orientation:

Atomic Number	Atomic Type	Coordinates (Angstroms) X	Y	Z
6	0	9.593222	-2.535779	0.027481
6	0	8.186391	-2.750628	0.145977
7	0	7.527257	-1.597324	0.072646
6	0	9.777450	-1.182504	-0.126242
6	0	8.475580	-0.577873	-0.097666
6	0	8.070108	0.724359	-0.194319
6	0	6.695805	1.080612	-0.121712
7	0	5.697883	0.134016	0.035384
6	0	4.494641	0.839699	0.084098
6	0	4.778833	2.254321	-0.071731
6	0	6.167427	2.372983	-0.191696
5	0	5.969608	-1.409814	0.135144
9	0	5.515594	-1.906064	1.353244
9	0	5.398593	-2.083560	-0.933076
6	0	3.729271	3.212737	-0.093238
6	0	2.372532	2.734100	-0.049927
6	0	3.992538	4.601208	-0.172344
6	0	2.956441	5.509116	-0.213337
6	0	1.615981	5.059478	-0.177939
6	0	1.335633	3.714174	-0.096869
6	0	2.058002	1.336903	0.026134

6	0	3.082973	0.358220	0.103765
6	0	2.800544	-1.123277	0.162852
1	0	7.660871	-3.687582	0.277578
1	0	10.707275	-0.643317	-0.244726
1	0	3.872023	0.733412	1.175011
1	0	6.747832	3.275209	-0.323098
1	0	5.020881	4.947189	-0.193713
1	0	3.168642	6.572295	-0.272276
1	0	0.805365	5.780811	-0.213684
1	0	0.303979	3.379620	-0.071145
1	0	3.033747	-1.580440	-0.803853
1	0	1.749654	-1.297814	0.395086
1	0	3.416366	-1.625147	0.909902
1	0	10.346116	-3.310466	0.056957
79	0	0.086146	0.712007	-0.015269
15	0	-2.151611	-0.147615	-0.063446
6	0	-2.143099	-1.669194	-1.093170
6	0	-1.304659	-2.712812	-0.681255
6	0	-2.792257	-1.833498	-2.320581
6	0	-1.125344	-3.870260	-1.424861
6	0	-2.627258	-2.985470	-3.087655
6	0	-1.795187	-4.006066	-2.639570
6	0	-3.490247	0.943991	-0.670745
6	0	-4.835681	0.554722	-0.621377
6	0	-3.220534	2.235401	-1.139307
6	0	-5.867438	1.401568	-1.001151
6	0	-4.238459	3.103507	-1.525937
6	0	-5.563560	2.685359	-1.453727
6	0	-2.740854	-0.638783	1.610756
6	0	-3.409582	-1.824009	1.940134
6	0	-2.477276	0.247716	2.664121
6	0	-3.774663	-2.121459	3.250414
6	0	-2.837023	-0.024465	3.978143
6	0	-3.487150	-1.220677	4.271711
9	0	-1.869884	1.420057	2.416817
9	0	-3.727474	-2.725550	1.004317
9	0	-4.406039	-3.264368	3.527849
9	0	-3.840266	-1.497334	5.524143
9	0	-2.568805	0.849927	4.950211
9	0	-5.155115	-0.681060	-0.214848
9	0	-7.137219	0.996728	-0.943815
9	0	-6.541507	3.506778	-1.822473
9	0	-3.947075	4.329377	-1.965462
9	0	-1.964530	2.690607	-1.240196
9	0	-0.644645	-2.602296	0.485870
9	0	-3.589200	-0.881828	-2.824162
9	0	-3.264506	-3.107862	-4.254208
9	0	-1.636496	-5.107857	-3.368872
9	0	-0.322332	-4.842370	-0.987986
1	0	8.801395	1.513684	-0.327566

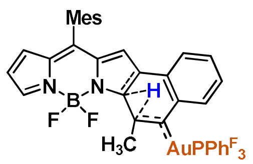

Standard orientation:

Atomic Number	Atomic Type	Coordinates (Angstroms) X	Y	Z
6	0	-7.906020	4.113551	0.095752
6	0	-6.481176	4.112236	0.155037
7	0	-6.011541	2.867022	0.131234
6	0	-8.302045	2.798309	0.033006
6	0	-7.109649	1.998221	0.055118
6	0	-6.933789	0.633897	0.016771
6	0	-5.603141	0.093814	0.056892
7	0	-4.471166	0.888374	0.139401
6	0	-3.379649	0.018299	0.168669
6	0	-3.870353	-1.342857	0.068050
6	0	-5.265660	-1.262002	0.011717
5	0	-4.502341	2.454314	0.172065
9	0	-3.946836	2.932014	1.356520
9	0	-3.862258	2.986011	-0.937406
6	0	-2.973675	-2.444675	0.028390
6	0	-1.561287	-2.168851	0.011187
6	0	-3.438805	-3.781245	-0.013926
6	0	-2.548058	-4.830908	-0.075232
6	0	-1.156015	-4.581269	-0.098850
6	0	-0.680271	-3.290377	-0.055323
6	0	-1.044734	-0.831582	0.049994
6	0	-1.912098	0.288359	0.132621
6	0	-1.409262	1.711491	0.147367
1	0	-5.811396	4.960438	0.212610
1	0	-9.307833	2.406421	-0.021613
1	0	-2.710645	0.057055	1.236937
1	0	-5.974210	-2.073319	-0.061786
1	0	-4.506393	-3.974654	0.005172
1	0	-2.914787	-5.852397	-0.106435
1	0	-0.460920	-5.413614	-0.151593
1	0	0.389285	-3.109547	-0.077959
1	0	-1.572919	2.168455	-0.833629
1	0	-0.343155	1.731795	0.376334
1	0	-1.939808	2.323300	0.877176
1	0	-8.530055	4.995826	0.100810
79	0	0.997467	-0.510599	-0.030132
15	0	3.343180	-0.017640	-0.082251
6	0	3.575031	1.529171	-1.046182
6	0	2.923081	2.676326	-0.576360
6	0	4.230853	1.640402	-2.276091
6	0	2.929136	3.879670	-1.266774
6	0	4.249420	2.836862	-2.990731
6	0	3.600657	3.958667	-2.485515
6	0	4.484478	-1.282977	-0.751939
6	0	5.874446	-1.106005	-0.723441
6	0	4.010522	-2.502860	-1.248949
6	0	6.756411	-2.087090	-1.153937
6	0	4.875516	-3.502801	-1.686443
6	0	6.250188	-3.293794	-1.636571
6	0	4.021373	0.294043	1.601657
6	0	4.867936	1.344447	1.975745
6	0	3.639742	-0.596760	2.614365
6	0	5.290517	1.513766	3.291651
6	0	4.053397	-0.452196	3.932692
6	0	4.882208	0.614401	4.272277
9	0	2.857191	-1.648144	2.320154
9	0	5.308704	2.234532	1.079596
9	0	6.092829	2.531132	3.612897
9	0	5.289264	0.768176	5.529498
9	0	3.666886	-1.325679	4.864957
9	0	6.387336	0.052482	-0.287758
9	0	8.074180	-1.882773	-1.116807
9	0	7.082886	-4.242174	-2.054096
9	0	4.390892	-4.655659	-2.152210
9	0	2.697836	-2.759506	-1.327106
9	0	2.264200	2.623432	0.595600
9	0	4.857083	0.595254	-2.832759
9	0	4.886776	2.905475	-4.161643
9	0	3.617514	5.103154	-3.164401
9	0	2.300626	4.949387	-0.775397
6	0	-8.105143	-0.290292	-0.066493
6	0	-8.691064	-0.780285	1.121138
6	0	-8.610798	-0.664224	-1.330040

6	0	-9.784676	-1.645374	1.016964
6	0	-9.705925	-1.533338	-1.378995
6	0	-10.309995	-2.032332	-0.220598
1	0	-10.236561	-2.026667	1.929936
1	0	-10.094521	-1.827614	-2.351328
6	0	-8.164964	-0.388362	2.484306
1	0	-7.108243	-0.655107	2.604519
1	0	-8.243969	0.692157	2.653504
1	0	-8.728231	-0.891642	3.274817
6	0	-11.511130	-2.944895	-0.301030
1	0	-11.523745	-3.663038	0.525496
1	0	-12.445002	-2.370008	-0.245803
1	0	-11.527447	-3.503213	-1.242465
6	0	-7.996611	-0.150504	-2.613608
1	0	-8.070773	0.940719	-2.689729
1	0	-6.932505	-0.405073	-2.685984
1	0	-8.502607	-0.580141	-3.482348

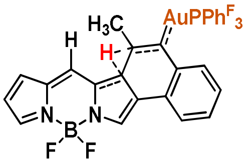

Standard orientation:

Atomic Number	Atomic Type	Coordinates (Angstroms) X	Y	Z
6	0	-9.112593	3.347557	0.446766
6	0	-9.431028	1.971548	0.376459
7	0	-8.319316	1.243249	0.185628
6	0	-7.739740	3.449069	0.288249
6	0	-7.239309	2.126616	0.125950
6	0	-5.939404	1.664570	-0.024948
6	0	-5.648965	0.300268	-0.102556
7	0	-6.673347	-0.630726	-0.045028
6	0	-6.185950	-1.877732	-0.072092
6	0	-4.781762	-1.846854	-0.164581
6	0	-4.411276	-0.453913	-0.196213
5	0	-8.228485	-0.291031	-0.040253
9	0	-8.752126	-0.639317	-1.273387
9	0	-8.834366	-0.987197	0.986561
6	0	-3.803190	-2.872729	-0.211345
6	0	-2.414911	-2.477019	-0.234514
6	0	-4.152183	-4.244766	-0.229978
6	0	-3.173646	-5.214001	-0.269055
6	0	-1.807782	-4.847575	-0.292778
6	0	-1.442928	-3.519997	-0.277154
6	0	-2.007076	-1.099302	-0.197018
6	0	-2.973505	-0.064547	-0.184190
6	0	-2.641306	1.405549	-0.080956
1	0	-10.399961	1.496108	0.453323
1	0	-7.134120	4.345188	0.291826
1	0	-6.849052	-2.729460	-0.023086
1	0	-3.760827	-0.275373	-1.264689
1	0	-5.199416	-4.529261	-0.218621
1	0	-3.451217	-6.263514	-0.282638
1	0	-1.044690	-5.618971	-0.323807
1	0	-0.392404	-3.250888	-0.298058
1	0	-3.123416	1.994390	-0.868244
1	0	-1.565024	1.563238	-0.158589
1	0	-2.976148	1.797223	0.887604
1	0	-9.821486	4.148665	0.600906
79	0	-0.002149	-0.597977	-0.125120
15	0	2.282434	0.125898	-0.049214
6	0	2.462850	1.558049	-1.186229
6	0	1.661589	2.679169	-0.936264
6	0	3.229778	1.585007	-2.355424
6	0	1.626556	3.780946	-1.778496
6	0	3.211309	2.678931	-3.218990
6	0	2.411177	3.779345	-2.930398
6	0	3.598046	-1.081764	-0.449289
6	0	4.954563	-0.760227	-0.305400
6	0	3.294347	-2.388612	-0.848115
6	0	5.963800	-1.685837	-0.529858
6	0	4.289048	-3.335414	-1.078783
6	0	5.625234	-2.982351	-0.917098
6	0	2.755354	0.707725	1.632737
6	0	3.448083	1.884610	1.940784
6	0	2.368249	-0.095791	2.714216
6	0	3.717143	2.255327	3.255654
6	0	2.631734	0.249330	4.033839
6	0	3.307253	1.437025	4.304327
9	0	1.732177	-1.257730	2.490312
9	0	3.883900	2.705747	0.978770
9	0	4.374308	3.388451	3.512109
9	0	3.568218	1.783732	5.561786
9	0	2.247117	-0.546999	5.033289
9	0	5.308684	0.484999	0.039064
9	0	7.245307	-1.344315	-0.388095
9	0	6.581482	-3.878725	-1.137764
9	0	3.965358	-4.573823	-1.455614
9	0	2.026781	-2.782772	-1.030317
9	0	0.891636	2.703822	0.167064
9	0	4.003418	0.550782	-2.709948
9	0	3.957688	2.668352	-4.325361
9	0	2.391116	4.825630	-3.752193
9	0	0.852053	4.830003	-1.494251
1	0	-5.140058	2.392878	-0.057982

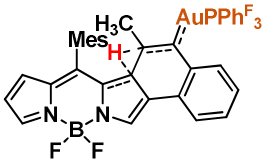

Standard orientation:

Atomic Number	Atomic Type	Coordinates (Angstroms) X	Y	Z
6	0	-9.155622	1.511040	0.642852
6	0	-9.209009	0.122502	0.403387
7	0	-7.982561	-0.344051	0.115000
6	0	-7.830452	1.892238	0.489584
6	0	-7.087236	0.723350	0.154549
6	0	-5.702002	0.573531	-0.044458
6	0	-5.149395	-0.715528	-0.231950
7	0	-6.013683	-1.808408	-0.160217
6	0	-5.351015	-2.958345	-0.082782
6	0	-3.967496	-2.723077	-0.142520
6	0	-3.793535	-1.290774	-0.305821
5	0	-7.590627	-1.764804	-0.318505
9	0	-7.892912	-1.986300	-1.654131
9	0	-8.150721	-2.728763	0.497238
6	0	-2.883082	-3.629995	-0.076947
6	0	-1.550410	-3.087358	-0.121318
6	0	-3.076270	-5.028855	0.050658
6	0	-1.994389	-5.874255	0.150967
6	0	-0.675914	-5.357796	0.120414
6	0	-0.462521	-4.005736	-0.014935
6	0	-1.319531	-1.687127	-0.287125
6	0	-2.391587	-0.770300	-0.440601
6	0	-2.114105	0.681702	-0.750094
1	0	-10.061599	-0.543044	0.430135

1	0	-7.413130	2.881275	0.612081
1	0	-5.884690	-3.893381	0.005713
1	0	-3.216774	-1.216271	-1.424764
1	0	-4.083205	-5.431852	0.074160
1	0	-2.151547	-6.943597	0.253357
1	0	0.168335	-6.035598	0.198735
1	0	0.550247	-3.618627	-0.057667
1	0	-2.906150	1.132453	-1.345626
1	0	-1.177837	0.762609	-1.306157
1	0	-2.018547	1.265500	0.169809
1	0	-9.995273	2.138128	0.907229
79	0	0.610536	-0.941384	-0.217112
15	0	2.795259	0.029464	-0.085280
6	0	2.715663	1.680902	-0.885728
6	0	1.844377	2.621914	-0.322350
6	0	3.340250	2.041585	-2.083483
6	0	1.619552	3.867822	-0.889898
6	0	3.124177	3.283903	-2.677327
6	0	2.264833	4.200022	-2.079586
6	0	4.196141	-0.896441	-0.814165
6	0	5.509940	-0.414581	-0.737446
6	0	4.014350	-2.153682	-1.403007
6	0	6.594278	-1.136262	-1.215779
6	0	5.086862	-2.897198	-1.889415
6	0	6.377809	-2.386771	-1.794556
6	0	3.345003	0.298243	1.651532
6	0	3.953910	1.451217	2.161535
6	0	3.123374	-0.748965	2.556765
6	0	4.301422	1.564440	3.505135
6	0	3.467595	-0.661799	3.899850
6	0	4.057821	0.506436	4.376069
9	0	2.573629	-1.897961	2.129606
9	0	4.227002	2.498621	1.375535
9	0	4.874350	2.681768	3.957783
9	0	4.394794	0.607771	5.658890
9	0	3.241190	-1.684963	4.726215
9	0	5.742862	0.791684	-0.203203
9	0	7.831176	-0.644063	-1.131751
9	0	7.406609	-3.089184	-2.258097
9	0	4.880089	-4.093271	-2.443690
9	0	2.796024	-2.698073	-1.521516
9	0	1.200636	2.319905	0.818217
9	0	4.160914	1.199665	-2.724882
9	0	3.738543	3.593358	-3.821092
9	0	2.058196	5.388466	-2.641099
9	0	0.795523	4.743655	-0.306529
6	0	-4.903622	1.834231	0.062562
6	0	-4.304096	2.171235	1.294825
6	0	-4.841203	2.719084	-1.035708
6	0	-3.607609	3.381244	1.389783
6	0	-4.123716	3.911089	-0.892949
6	0	-3.491153	4.258136	0.305938
1	0	-3.145381	3.643976	2.338684
1	0	-4.059700	4.586863	-1.742834
6	0	-2.686113	5.531044	0.418768
6	0	-5.514073	2.401286	-2.353777
6	0	-4.409545	1.269071	2.504985
1	0	-1.639580	5.354883	0.138914
1	0	-3.074814	6.310694	-0.244446
1	0	-2.689099	5.917253	1.443259
1	0	-5.263717	1.395515	-2.711507
1	0	-6.606226	2.444694	-2.268630
1	0	-5.211376	3.116666	-3.123359
1	0	-3.952027	1.741806	3.378374
1	0	-5.453877	1.045515	2.751702
1	0	-3.905527	0.307771	2.346891

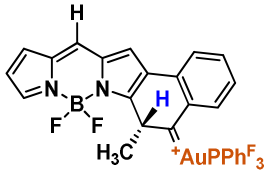

Standard orientation:

Atomic Number	Atomic Type	Coordinates (Angstroms) X	Y	Z
6	0	9.031078	-2.873550	0.874087
6	0	7.648490	-2.965411	0.572642
7	0	7.142768	-1.753778	0.315369
6	0	9.370128	-1.535822	0.788467
6	0	8.184170	-0.824767	0.439236
6	0	7.944727	0.525550	0.275550
6	0	6.658586	1.017613	0.001928
7	0	5.575875	0.137609	-0.167898
6	0	4.470183	0.886347	-0.309967
6	0	4.800745	2.262513	-0.235931
6	0	6.193554	2.337960	-0.051710
5	0	5.693683	-1.428733	-0.168257
9	0	4.754230	-1.970907	0.698488
9	0	5.519854	-1.926041	-1.458743
6	0	3.768125	3.256377	-0.282700
6	0	2.380769	2.822797	-0.311535
6	0	4.052550	4.632272	-0.255711
6	0	3.028721	5.566996	-0.244447
6	0	1.672488	5.170724	-0.254175
6	0	1.360872	3.832417	-0.283974
6	0	2.026394	1.461520	-0.366456
6	0	3.079677	0.406386	-0.543660
6	0	2.928459	-0.140646	-2.014908
1	0	7.023692	-3.848135	0.534402
1	0	10.337251	-1.082681	0.960068
1	0	6.808481	3.218895	0.069916
1	0	5.084802	4.965709	-0.241174
1	0	3.275482	6.624559	-0.224460
1	0	0.888306	5.920065	-0.242361
1	0	0.323828	3.514610	-0.303096
1	0	3.073212	0.665094	-2.739835
1	0	1.931673	-0.567310	-2.149589
1	0	3.681760	-0.912305	-2.175619
1	0	9.674164	-3.704987	1.125689
79	0	0.097986	0.789403	-0.230720
15	0	-2.112571	-0.145256	-0.023027
6	0	-2.278307	-1.507982	-1.241035
6	0	-1.352405	-2.554962	-1.149282
6	0	-3.161014	-1.542018	-2.325318
6	0	-1.303938	-3.596349	-2.064847
6	0	-3.130130	-2.574955	-3.260296
6	0	-2.202807	-3.603884	-3.130215
6	0	-3.540040	0.981095	-0.225440
6	0	-4.850905	0.555551	0.029895
6	0	-3.366490	2.325652	-0.574405
6	0	-5.936793	1.417076	-0.038132
6	0	-4.439923	3.209435	-0.649069
6	0	-5.726275	2.753375	-0.377679
6	0	-2.377552	-0.875847	1.644723
6	0	-2.961829	-2.118190	1.919948
6	0	-1.931254	-0.132233	2.745834
6	0	-3.073255	-2.604464	3.219764
6	0	-2.036692	-0.593934	4.051715
6	0	-2.609048	-1.841704	4.287604
9	0	-1.390677	1.083486	2.557894
9	0	-3.439798	-2.892371	0.939717

9	0	-3.631228	-3.795749	3.444627
9	0	-2.720182	-2.299097	5.531503
9	0	-1.600268	0.148238	5.071189
9	0	-5.085551	-0.728269	0.330745
9	0	-7.171355	0.977196	0.208932
9	0	-6.757159	3.589227	-0.448633
9	0	-4.238832	4.485891	-0.981321
9	0	-2.153323	2.819687	-0.857867
9	0	-0.473639	-2.563173	-0.131879
9	0	-4.065494	-0.574451	-2.523401
9	0	-3.988090	-2.575866	-4.282804
9	0	-2.172639	-4.591967	-4.020547
9	0	-0.408706	-4.576844	-1.933773
1	0	2.864923	-0.442595	0.115911
1	0	8.760564	1.231386	0.391255

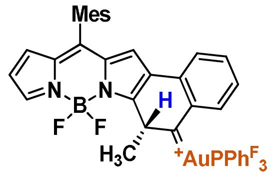

Standard orientation:

Atomic Number	Atomic Type	Coordinates (Angstroms) X	Y	Z
6	0	7.347854	-4.142943	0.823345
6	0	5.961059	-4.047030	0.553028
7	0	5.629917	-2.787605	0.243261
6	0	7.874214	-2.873841	0.661018
6	0	6.795998	-2.013006	0.299070
6	0	6.768390	-0.640048	0.076470
6	0	5.535116	0.000262	-0.194906
7	0	4.337364	-0.729450	-0.299765
6	0	3.338997	0.154305	-0.455783
6	0	3.852036	1.474016	-0.453462
6	0	5.247338	1.367713	-0.302448
5	0	4.235525	-2.291501	-0.240045
9	0	3.242365	-2.667953	0.655577
9	0	3.974260	-2.808210	-1.508823
6	0	2.959860	2.594379	-0.522677
6	0	1.526461	2.349928	-0.496478
6	0	3.424631	3.919944	-0.568117
6	0	2.534567	4.982742	-0.575691
6	0	1.137837	4.771679	-0.532052
6	0	0.650640	3.487069	-0.490037
6	0	0.993259	1.047315	-0.481884
6	0	1.892577	-0.142878	-0.648521
6	0	1.643132	-0.689355	-2.107874
1	0	5.213219	-4.829125	0.569799
1	0	8.900588	-2.559827	0.790352
1	0	5.976544	2.162651	-0.237256
1	0	4.492042	4.112073	-0.594741
1	0	2.920168	5.997597	-0.612276
1	0	0.460923	5.619271	-0.536241
1	0	-0.419620	3.309785	-0.468267
1	0	1.900266	0.071146	-2.850419
1	0	0.592107	-0.962591	-2.226946
1	0	2.270440	-1.568634	-2.257380
1	0	7.871709	-5.045209	1.105600
79	0	-1.000425	0.638319	-0.265627
15	0	-3.307934	0.005790	0.000770
6	0	-3.647320	-1.395376	-1.135406
6	0	-2.863269	-2.545302	-0.977110
6	0	-4.527007	-1.378906	-2.222678
6	0	-2.949277	-3.635376	-1.831135
6	0	-4.629297	-2.459616	-3.096735
6	0	-3.842027	-3.589638	-2.900740
6	0	-4.581805	1.289811	-0.274875
6	0	-5.933905	1.052149	0.008168
6	0	-4.241957	2.574775	-0.714313
6	0	-6.902318	2.038262	-0.118388
6	0	-5.195483	3.580362	-0.849702
6	0	-6.527016	3.310715	-0.548435
6	0	-3.655560	-0.580834	1.709297
6	0	-4.380343	-1.726025	2.060336
6	0	-3.123804	0.175692	2.762452
6	0	-4.544766	-2.111255	3.388119
6	0	-3.280299	-0.184649	4.094808
6	0	-3.993240	-1.339805	4.407142
9	0	-2.446821	1.305783	2.498467
9	0	-4.948104	-2.499959	1.128964
9	0	-5.237018	-3.212592	3.686423
9	0	-4.154059	-1.700105	5.677298
9	0	-2.759055	0.566559	5.066743
9	0	-6.327351	-0.168164	0.395190
9	0	-8.180879	1.776477	0.156671
9	0	-7.444328	4.263826	-0.677019
9	0	-4.837285	4.795345	-1.268555
9	0	-2.978283	2.889863	-1.030577
9	0	-1.994050	-2.608835	0.046334
9	0	-5.298566	-0.315902	-2.483867
9	0	-5.480105	-2.409262	-4.124114
9	0	-3.938773	-4.623566	-3.732531
9	0	-2.188196	-4.713776	-1.636798
1	0	1.580412	-0.943835	0.030937
6	0	8.025297	0.166357	0.151205
6	0	8.771860	0.395378	-1.024777
6	0	8.451051	0.691244	1.390194
6	0	9.944475	1.152383	-0.934830
6	0	9.630574	1.443187	1.426336
6	0	10.394038	1.682577	0.279187
1	0	10.520464	1.331514	-1.840134
1	0	9.958787	1.852916	2.379131
6	0	7.663351	0.463004	2.661856
1	0	6.636514	0.838527	2.576102
1	0	7.594649	-0.602704	2.909682
1	0	8.136891	0.972765	3.505690
6	0	8.331214	-0.159692	-2.361536
1	0	8.258685	-1.253820	-2.340247
1	0	7.344757	0.220272	-2.652466
1	0	9.041312	0.113680	-3.147205
6	0	11.680910	2.471082	0.352430
1	0	12.539823	1.807459	0.519035
1	0	11.868921	3.016900	-0.578186

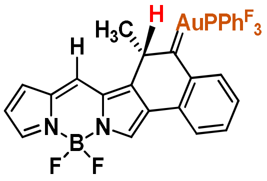

Standard orientation:

Atomic Number	Atomic Type	Coordinates (Angstroms) X	Y	Z
6	0	-8.987606	3.431840	-0.485817
6	0	-9.344461	2.063830	-0.402688
7	0	-8.253217	1.302834	-0.244810
6	0	-7.609466	3.492512	-0.371202
6	0	-7.142964	2.155108	-0.220761

6	0	-5.859562	1.649881	-0.099048
6	0	-5.609818	0.274345	-0.013379
7	0	-6.682331	-0.632316	-0.057240
6	0	-6.197844	-1.873321	-0.043122
6	0	-4.779977	-1.827811	0.019093
6	0	-4.413005	-0.472538	0.044901
5	0	-8.209682	-0.243966	-0.033209
9	0	-8.874382	-0.897174	-1.056173
9	0	-8.744302	-0.573301	1.203688
6	0	-3.804070	-2.875179	0.014596
6	0	-2.399069	-2.505543	-0.018793
6	0	-4.154274	-4.235888	0.018087
6	0	-3.175805	-5.216891	-0.024404
6	0	-1.803149	-4.884840	-0.073286
6	0	-1.428473	-3.562231	-0.070583
6	0	-1.983032	-1.161636	0.007220
6	0	-2.984904	-0.045670	0.143749
6	0	-2.712192	0.695621	1.501316
1	0	-10.329635	1.618234	-0.449301
1	0	-6.978231	4.371044	-0.393459
1	0	-6.858685	-2.728090	-0.082671
1	0	-5.199941	-4.522644	0.052697
1	0	-3.471824	-6.261946	-0.021504
1	0	-1.056071	-5.669944	-0.108268
1	0	-0.377494	-3.293769	-0.099948
1	0	-3.458989	1.480365	1.643021
1	0	-1.717109	1.147007	1.497460
1	0	-2.783427	-0.003492	2.338490
1	0	-9.677957	4.253155	-0.618032
79	0	-0.011112	-0.610562	-0.086594
15	0	2.263373	0.181185	-0.106487
6	0	2.231975	1.978876	-0.477656
6	0	1.471336	2.786456	0.377922
6	0	2.794153	2.603501	-1.595202
6	0	1.281787	4.142885	0.158241
6	0	2.617177	3.964207	-1.838990
6	0	1.862274	4.735743	-0.961637
6	0	3.441302	-0.634053	-1.242764
6	0	4.805234	-0.312164	-1.237821
6	0	3.035890	-1.672371	-2.090625
6	0	5.728493	-0.984958	-2.024896
6	0	3.944490	-2.363462	-2.888282
6	0	5.291933	-2.018989	-2.852818
6	0	3.068363	-0.012804	1.536912
6	0	3.841825	0.948639	2.198828
6	0	2.884338	-1.235929	2.196410
6	0	4.381836	0.712990	3.460496
6	0	3.417876	-1.495965	3.451959
6	0	4.168738	-0.510691	4.088863
9	0	2.182629	-2.216150	1.602773
9	0	4.095976	2.138404	1.643027
9	0	5.110257	1.654244	4.064477
9	0	4.688185	-0.742108	5.291027
9	0	3.219257	-2.675821	4.042597
9	0	5.245950	0.689600	-0.465378
9	0	7.019269	-0.648618	-1.999816
9	0	6.165537	-2.672828	-3.612359
9	0	3.526967	-3.350862	-3.683110
9	0	1.752667	-2.047663	-2.165870
9	0	0.899460	2.235386	1.464202
9	0	3.514464	1.918443	-2.492264
9	0	3.168915	4.526560	-2.916118
9	0	1.691887	6.034917	-1.191542
9	0	0.552684	4.873813	1.003700
1	0	-2.753805	0.684242	-0.647282
1	0	-5.027202	2.346098	-0.097249

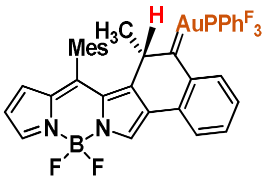

Standard orientation:

Atomic Number	Atomic Type	Coordinates (Angstroms) X	Y	Z
6	0	8.834031	1.948040	1.002326
6	0	9.012620	0.553680	0.848290
7	0	7.857871	-0.029009	0.498014
6	0	7.502641	2.211814	0.727075
6	0	6.881281	0.967767	0.408920
6	0	5.544799	0.681011	0.115204
6	0	5.129514	-0.661113	-0.059945
7	0	6.078839	-1.695223	0.053626
6	0	5.460142	-2.870943	-0.013694
6	0	4.069839	-2.663797	-0.182846
6	0	3.859897	-1.272933	-0.226643
5	0	7.637807	-1.517661	0.117043
9	0	8.166720	-2.361121	1.079477
9	0	8.179757	-1.782754	-1.134744
6	0	2.991641	-3.605999	-0.238568
6	0	1.633731	-3.096341	-0.243585
6	0	3.199417	-4.995736	-0.244790
6	0	2.123419	-5.870685	-0.244939
6	0	0.791795	-5.398596	-0.236949
6	0	0.556760	-4.044050	-0.233352
6	0	1.369213	-1.714142	-0.265763
6	0	2.485941	-0.728277	-0.463140
6	0	2.345763	-0.245127	-1.957789
1	0	9.912702	-0.033486	0.974922
1	0	6.999643	3.168131	0.756533
1	0	6.017616	-3.794057	0.064887
1	0	4.209632	-5.390723	-0.248486
1	0	2.310167	-6.940683	-0.250070
1	0	-0.033543	-6.102624	-0.239318
1	0	-0.460252	-3.665910	-0.246388
1	0	3.098687	0.521218	-2.146317
1	0	1.354004	0.180557	-2.127748
1	0	2.501616	-1.079619	-2.646229
1	0	9.601591	2.653317	1.289174
79	0	-0.522124	-0.947660	-0.092686
15	0	-2.700657	0.057738	0.098715
6	0	-2.480846	1.879426	0.122923
6	0	-1.813904	2.446479	-0.970155
6	0	-2.793180	2.735417	1.183547
6	0	-1.486734	3.792531	-1.029454
6	0	-2.465473	4.089788	1.151215
6	0	-1.810240	4.618873	0.044628
6	0	-3.737984	-0.421724	1.525812
6	0	-5.062242	0.021940	1.645925
6	0	-3.272651	-1.308426	2.504054
6	0	-5.892619	-0.394592	2.676529
6	0	-4.087122	-1.740084	3.547926
6	0	-5.398732	-1.282942	3.631835
6	0	-3.763731	-0.346198	-1.346794
6	0	-4.545350	0.555196	-2.078729
6	0	-3.781157	-1.682764	-1.768478
6	0	-5.285052	0.151562	-3.187238
6	0	-4.516547	-2.110575	-2.866155
6	0	-5.270273	-1.183233	-3.582143
9	0	-3.083264	-2.607563	-1.086955
9	0	-4.615370	1.847112	-1.739227

9	0	-6.014405	1.039321	-3.865835
9	0	-5.981962	-1.574793	-4.635034
9	0	-4.507661	-3.394137	-3.230752
9	0	-5.552109	0.893845	0.755101
9	0	-7.146600	0.050984	2.764030
9	0	-6.181652	-1.688312	4.626101
9	0	-3.614186	-2.586532	4.464001
9	0	-2.020128	-1.781440	2.474863
9	0	-1.474409	1.663304	-2.008534
9	0	-3.400820	2.288209	2.289040
9	0	-2.771697	4.875793	2.184751
9	0	-1.479293	5.907271	0.014474
9	0	-0.846796	4.292480	-2.090136
1	0	2.295461	0.158166	0.150060
6	0	4.583655	1.826225	0.048479
6	0	4.512337	2.605038	-1.129523
6	0	3.814919	2.165165	1.182369
6	0	3.642023	3.698467	-1.156899
6	0	2.964665	3.275769	1.105851
6	0	2.861712	4.054747	-0.050621
1	0	3.582219	4.295293	-2.064644
1	0	2.381040	3.546406	1.982897
6	0	3.902302	1.376034	2.471344
6	0	5.371040	2.301955	-2.339694
6	0	1.953059	5.260404	-0.107200
1	0	3.404544	0.401004	2.391474
1	0	4.941574	1.181704	2.757605
1	0	3.424381	1.922980	3.289610
1	0	4.996104	2.830758	-3.221005
1	0	6.408761	2.619382	-2.178891
1	0	5.399588	1.231915	-2.573512
1	0	1.377163	5.372672	0.816584
1	0	2.530269	6.181828	-0.254485
1	0	1.247632	5.189448	-0.942484

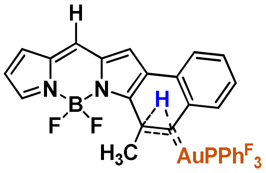

Standard orientation:

Atomic Number	Atomic Type	Coordinates (Angstroms) X	Y	Z
6	0	9.591420	-2.543814	0.147232
6	0	8.184638	-2.755267	0.217897
7	0	7.531076	-1.597403	0.123161
6	0	9.783206	-1.187329	0.002403
6	0	8.485388	-0.582715	-0.013052
6	0	8.080027	0.721891	-0.124806
6	0	6.711943	1.083533	-0.104206
7	0	5.694726	0.141531	0.028476
6	0	4.513867	0.839680	0.005753
6	0	4.782672	2.228285	-0.138819
6	0	6.175121	2.370471	-0.205146
5	0	5.974240	-1.406650	0.143187
9	0	5.490497	-1.904036	1.348703
9	0	5.432919	-2.080992	-0.941523
6	0	3.729014	3.206320	-0.180791
6	0	2.382681	2.752265	-0.058700
6	0	3.988980	4.583321	-0.325851
6	0	2.949211	5.498052	-0.342818
6	0	1.618096	5.063053	-0.211211
6	0	1.343971	3.714356	-0.069383
6	0	2.068555	1.330970	0.038623
6	0	3.141982	0.355575	0.106037
6	0	2.830634	-1.124341	0.077177
1	0	7.651271	-3.690063	0.331500
1	0	10.718273	-0.650787	-0.083858
1	0	6.751694	3.278846	-0.310163
1	0	5.013692	4.926622	-0.424816
1	0	3.164344	6.556446	-0.456106
1	0	0.806824	5.784127	-0.220800
1	0	0.315694	3.382261	0.027370
1	0	3.091598	-1.514338	-0.910470
1	0	1.768599	-1.301274	0.252215
1	0	3.419817	-1.668479	0.813660
1	0	10.342666	-3.318891	0.200531
79	0	0.081091	0.698561	0.010255
15	0	-2.151431	-0.144040	-0.057319
6	0	-2.201397	-1.524548	-1.266339
6	0	-1.349701	-2.610734	-1.028765
6	0	-2.918966	-1.539055	-2.466448
6	0	-1.219362	-3.669994	-1.915032
6	0	-2.803437	-2.589916	-3.374623
6	0	-1.955609	-3.657611	-3.098583
6	0	-3.502687	1.021273	-0.461197
6	0	-4.848039	0.635576	-0.383781
6	0	-3.243622	2.356253	-0.793605
6	0	-5.888585	1.526605	-0.605535
6	0	-4.270361	3.268928	-1.020721
6	0	-5.594525	2.852448	-0.923509
6	0	-2.661878	-0.832512	1.571507
6	0	-3.317904	-2.050854	1.785761
6	0	-2.350418	-0.076772	2.710355
6	0	-3.624265	-2.503974	3.066110
6	0	-2.652337	-0.504749	3.997018
6	0	-3.290759	-1.730007	4.173920
9	0	-1.751248	1.118302	2.577228
9	0	-3.680069	-2.833619	0.763515
9	0	-4.244532	-3.673817	3.232785
9	0	-3.588179	-2.155520	5.398381
9	0	-2.339776	0.248365	5.053351
9	0	-5.159217	-0.637569	-0.107472
9	0	-7.157770	1.124640	-0.526239
9	0	-6.581025	3.716033	-1.140611
9	0	-3.988364	4.535104	-1.332617
9	0	-1.989202	2.811863	-0.912324
9	0	-0.628280	-2.641988	0.106327
9	0	-3.737139	-0.534465	-2.805333
9	0	-3.503862	-2.570556	-4.510444
9	0	-1.844628	-4.663187	-3.962568
9	0	-0.401493	-4.688327	-1.643091
1	0	2.587524	0.824409	1.177650
1	0	8.818440	1.509703	-0.228394

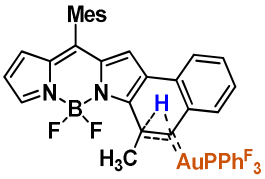

Standard orientation:

Atomic Number	Atomic Type	Coordinates (Angstroms) X	Y	Z
6	0	7.876661	-4.114654	0.254941
6	0	6.454920	-4.103434	0.298180
7	0	5.993565	-2.854802	0.217147
6	0	8.281031	-2.802015	0.141495

6	0	7.095600	-1.998455	0.118008
6	0	6.921704	-0.632555	0.029747
6	0	5.602505	-0.082417	0.043254
7	0	4.452516	-0.864108	0.135649
6	0	3.387529	0.001583	0.120189
6	0	3.861679	1.337077	0.023754
6	0	5.260549	1.271630	-0.024488
5	0	4.487321	-2.434698	0.209387
9	0	3.901618	-2.886423	1.388527
9	0	3.876359	-2.990445	-0.906582
6	0	2.968142	2.463783	0.005999
6	0	1.567091	2.213525	0.095469
6	0	3.433781	3.790765	-0.082044
6	0	2.542997	4.851154	-0.076323
6	0	1.159485	4.616816	0.022102
6	0	0.684140	3.320505	0.109298
6	0	1.043253	0.852895	0.136760
6	0	1.956986	-0.274877	0.183660
6	0	1.425652	-1.688518	0.093710
1	0	5.778002	-4.943543	0.383176
1	0	9.290024	-2.418833	0.082153
1	0	5.965929	2.086074	-0.097261
1	0	4.499879	3.980696	-0.152418
1	0	2.916327	5.868621	-0.145328
1	0	0.465696	5.451561	0.030492
1	0	-0.384056	3.143628	0.181765
1	0	1.650645	-2.080167	-0.902043
1	0	0.344779	-1.707686	0.240456
1	0	1.906602	-2.340230	0.821315
1	0	8.497135	-4.998130	0.304445
79	0	-1.014858	0.527843	0.056639
15	0	-3.344084	0.029248	-0.069536
6	0	-3.554409	-1.355100	-1.257265
6	0	-2.886955	-2.551452	-0.965376
6	0	-4.210192	-1.286880	-2.490482
6	0	-2.877441	-3.636323	-1.829925
6	0	-4.213752	-2.361985	-3.377429
6	0	-3.549162	-3.538545	-3.047291
6	0	-4.492557	1.371845	-0.543730
6	0	-5.880925	1.179717	-0.540177
6	0	-4.030322	2.654623	-0.861598
6	0	-6.772222	2.204070	-0.826272
6	0	-4.904909	3.698815	-1.151044
6	0	-6.277659	3.471971	-1.131425
6	0	-4.006877	-0.542806	1.549114
6	0	-4.856481	-1.637110	1.753752
6	0	-3.611056	0.168268	2.690677
6	0	-5.271928	-2.015506	3.027627
6	0	-4.016610	-0.187460	3.970884
6	0	-4.850760	-1.290218	4.138442
9	0	-2.822168	1.248553	2.567545
9	0	-5.305218	-2.369313	0.728465
9	0	-6.077847	-3.067500	3.185049
9	0	-5.250559	-1.644158	5.356525
9	0	-3.617297	0.519739	5.029679
9	0	-6.382055	-0.033787	-0.275248
9	0	-8.087597	1.983960	-0.819112
9	0	-7.119386	4.462105	-1.409452
9	0	-4.431554	4.910276	-1.448399
9	0	-2.720196	2.931482	-0.905531
9	0	-2.226958	-2.666285	0.201359
9	0	-4.849241	-0.177494	-2.883696
9	0	-4.851012	-2.261296	-4.545743
9	0	-3.551371	-4.566735	-3.891578
9	0	-2.233768	-4.759017	-1.505582
1	0	1.459970	0.234730	1.263980
6	0	8.099860	0.281594	-0.073970
6	0	8.597613	0.633490	-1.346568
6	0	8.696993	0.787087	1.101269
6	0	9.697760	1.494993	-1.417544
6	0	9.793710	1.645440	0.975474
6	0	10.312351	2.009742	-0.271837
1	0	10.081351	1.770811	-2.397283
1	0	10.253183	2.040157	1.878998
6	0	8.177757	0.420666	2.474202
1	0	7.117315	0.674018	2.588452
1	0	8.273119	-0.654172	2.668802
1	0	8.734352	0.950754	3.251783
6	0	7.969885	0.103480	-2.616897
1	0	8.024046	-0.990281	-2.669461
1	0	6.910134	0.375348	-2.689647
1	0	8.479639	0.505668	-3.496571
6	0	11.516530	2.916236	-0.374714
1	0	12.448710	2.343569	-0.281308
1	0	11.546666	3.434011	-1.338621
1	0	11.519025	3.669222	0.420477

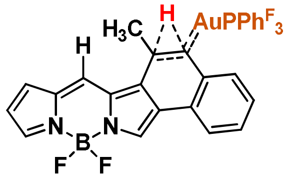

Standard orientation:

Atomic Number	Atomic Type	Coordinates (Angstroms) X	Y	Z
6	0	9.222375	3.351845	0.344099
6	0	9.517169	1.978657	0.166865
7	0	8.389040	1.254486	0.157143
6	0	7.845449	3.454131	0.444762
6	0	7.318254	2.135750	0.327137
6	0	6.009633	1.683331	0.352934
6	0	5.685516	0.325590	0.213620
7	0	6.710506	-0.599679	0.029806
6	0	6.192194	-1.824309	-0.109275
6	0	4.784131	-1.756243	-0.025389
6	0	4.455219	-0.398294	0.186317
5	0	8.262101	-0.289183	-0.004268
9	0	8.875623	-0.943294	1.050837
9	0	8.780324	-0.699288	-1.220484
6	0	3.787297	-2.784195	-0.127000
6	0	2.416625	-2.395028	-0.009118
6	0	4.111861	-4.138978	-0.336755
6	0	3.113450	-5.094306	-0.430078
6	0	1.761595	-4.723607	-0.316395
6	0	1.423300	-3.397851	-0.107265
6	0	2.033692	-0.996678	0.179092
6	0	3.066762	0.007162	0.320027
6	0	2.717296	1.477443	0.412002
1	0	10.482896	1.504959	0.048797
1	0	7.253872	4.348570	0.586172
1	0	6.831742	-2.681822	-0.262599
1	0	5.152351	-4.434102	-0.427024
1	0	3.377462	-6.135005	-0.591644
1	0	0.983555	-5.476751	-0.390773
1	0	0.378178	-3.117423	-0.026169
1	0	3.271269	1.973357	1.212742
1	0	1.649998	1.618670	0.591949
1	0	2.967481	1.959957	-0.539224
1	0	9.950465	4.149414	0.387931
79	0	0.020821	-0.453020	0.194533
15	0	-2.267067	0.212929	0.083924

6	0	-2.328099	2.008687	-0.289017
6	0	-1.691506	2.430343	-1.463488
6	0	-2.845328	3.009244	0.539653
6	0	-1.579909	3.766700	-1.818623
6	0	-2.743106	4.358825	0.206881
6	0	-2.112344	4.738767	-0.973388
6	0	-3.317270	-0.115526	1.544361
6	0	-4.694178	0.146261	1.520589
6	0	-2.803812	-0.719457	2.698491
6	0	-5.527975	-0.174826	2.581930
6	0	-3.621276	-1.052539	3.775709
6	0	-4.984660	-0.780770	3.715004
6	0	-3.159640	-0.655727	-1.270544
6	0	-4.008814	-0.067466	-2.215122
6	0	-2.959783	-2.038788	-1.378483
6	0	-4.602978	-0.811992	-3.230877
6	0	-3.545105	-2.803329	-2.379164
6	0	-4.369463	-2.181520	-3.314512
9	0	-2.195459	-2.675386	-0.473198
9	0	-4.287532	1.240160	-2.179457
9	0	-5.401873	-0.216630	-4.118433
9	0	-4.940631	-2.897240	-4.278816
9	0	-3.329614	-4.118450	-2.442977
9	0	-5.235510	0.744404	0.451110
9	0	-6.832963	0.094928	2.529517
9	0	-5.770900	-1.093776	4.739596
9	0	-3.102514	-1.630692	4.860386
9	0	-1.500989	-1.008558	2.812236
9	0	-1.167933	1.508298	-2.289839
9	0	-3.446957	2.717727	1.699708
9	0	-3.248168	5.287044	1.021842
9	0	-2.014868	6.026133	-1.294151
9	0	-0.969694	4.121496	-2.950809
1	0	2.537263	-0.550300	1.355334
1	0	5.224767	2.417615	0.476765

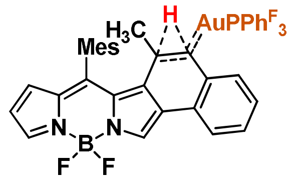

Standard orientation:

Atomic Number	Atomic Type	Coordinates (Angstroms) X	Y	Z
6	0	9.300240	1.400153	-0.300529
6	0	9.291471	-0.008023	-0.194444
7	0	8.039257	-0.450182	-0.011457
6	0	7.985512	1.817040	-0.171541
6	0	7.182676	0.649959	0.010447
6	0	5.787773	0.542945	0.130391
6	0	5.162756	-0.736834	0.188525
7	0	6.009681	-1.849178	0.089363
6	0	5.304482	-2.967632	-0.039068
6	0	3.931075	-2.664997	-0.008119
6	0	3.810822	-1.262027	0.170461
5	0	7.574551	-1.887081	0.279013
9	0	7.851222	-2.241562	1.593140
9	0	8.121820	-2.792977	-0.611830
6	0	2.821031	-3.563293	-0.141827
6	0	1.508575	-3.017174	-0.055831
6	0	2.987274	-4.946524	-0.359686
6	0	1.883978	-5.770676	-0.501108
6	0	0.582996	-5.239931	-0.423266
6	0	0.401176	-3.887175	-0.197430
6	0	1.313306	-1.593330	0.172662
6	0	2.458552	-0.715684	0.341244
6	0	2.200764	0.761864	0.532906
1	0	10.121331	-0.700693	-0.243368
1	0	7.611336	2.829603	-0.213529
1	0	5.800066	-3.922325	-0.139605
1	0	3.985073	-5.367730	-0.423595
1	0	2.025003	-6.833543	-0.672755
1	0	-0.277184	-5.892419	-0.534175
1	0	-0.603528	-3.482782	-0.131014
1	0	2.884611	1.200839	1.255609
1	0	1.176327	0.926949	0.873398
1	0	2.328471	1.284076	-0.418627
1	0	10.174420	2.014821	-0.462119
79	0	-0.622986	-0.818137	0.211718
15	0	-2.818627	0.106730	0.080674
6	0	-2.672781	1.826007	-0.545500
6	0	-2.049463	1.995074	-1.788520
6	0	-3.012787	2.990377	0.150557
6	0	-1.786553	3.243897	-2.332637
6	0	-2.757283	4.256378	-0.373444
6	0	-2.145835	4.384416	-1.616360
6	0	-3.827512	0.130664	1.605487
6	0	-5.164442	0.551643	1.585261
6	0	-3.326838	-0.340444	2.825107
6	0	-5.973799	0.503205	2.711041
6	0	-4.120061	-0.398385	3.968442
6	0	-5.444999	0.022762	3.909201
6	0	-3.877948	-0.817979	-1.106527
6	0	-4.696342	-0.260047	-2.095787
6	0	-3.853530	-2.216958	-1.025859
6	0	-5.428556	-1.053478	-2.975285
6	0	-4.578239	-3.029278	-1.888120
6	0	-5.368385	-2.440301	-2.872770
9	0	-3.123733	-2.818645	-0.069490
9	0	-4.810427	1.065099	-2.236469
9	0	-6.193676	-0.486977	-3.910187
9	0	-6.071052	-3.201637	-3.706438
9	0	-4.526520	-4.357605	-1.773516
9	0	-5.688045	1.039788	0.452891
9	0	-7.240561	0.916759	2.658005
9	0	-6.207811	-0.027689	4.996215
9	0	-3.614444	-0.855008	5.115534
9	0	-2.060607	-0.760332	2.942237
9	0	-1.690734	0.908020	-2.493282
9	0	-3.581470	2.942314	1.361810
9	0	-3.095536	5.346068	0.318840
9	0	-1.901627	5.591552	-2.119416
9	0	-1.196029	3.356870	-3.523745
1	0	1.909477	-1.263376	1.358673
6	0	5.070388	1.856636	0.112022
6	0	4.632317	2.392825	-1.117141
6	0	4.948343	2.597918	1.306928
6	0	4.032364	3.657285	-1.117619
6	0	4.337417	3.855091	1.254323
6	0	3.866253	4.401390	0.055285
1	0	3.694296	4.073775	-2.063812
1	0	4.233833	4.424293	2.175422
6	0	3.188231	5.751150	0.030562
6	0	5.457168	2.062026	2.627826
6	0	4.817823	1.648929	-2.421882
1	0	3.308532	6.241264	-0.940892
1	0	2.110246	5.654229	0.215901
1	0	3.590800	6.414672	0.802860
1	0	6.552133	2.006533	2.642873
1	0	5.144905	2.707788	3.453143
1	0	5.085813	1.050664	2.831354

1	0	5.880393	1.506835	-2.652852
1	0	4.362264	0.652477	-2.398342
1	0	4.367937	2.203738	-3.2497

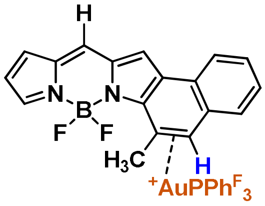

Standard orientation:

Atomic Number	Atomic Type	Coordinates (Angstroms) X	Y	Z
6	0	6.901706	-3.222820	1.196362
6	0	5.960651	-3.035701	0.146109
7	0	5.485082	-1.788842	0.159109
6	0	6.992058	-2.022073	1.868814
6	0	6.100105	-1.112355	1.218927
6	0	5.781467	0.200034	1.471822
6	0	4.836459	0.898383	0.688128
7	0	4.202618	0.308317	-0.398750
6	0	3.329095	1.240890	-0.901535
6	0	3.412056	2.440065	-0.122016
6	0	4.371364	2.208374	0.870785
5	0	4.458433	-1.171456	-0.853315
9	0	3.273522	-1.904025	-0.797447
9	0	5.013897	-1.212456	-2.124938
6	0	2.605226	3.599046	-0.409481
6	0	1.681831	3.502857	-1.486710
6	0	2.692347	4.806419	0.313709
6	0	1.892392	5.886418	-0.024090
6	0	0.984729	5.794615	-1.097366
6	0	0.880010	4.617296	-1.817948
6	0	1.597573	2.266595	-2.249292
6	0	2.442281	1.150903	-2.031625
6	0	2.498184	0.049981	-3.062780
1	0	5.628574	-3.751848	-0.594214
1	0	7.606705	-1.782106	2.725744
1	0	4.704999	2.882901	1.647205
1	0	3.394777	4.888914	1.137491
1	0	1.969951	6.811637	0.539160
1	0	0.368590	6.649064	-1.360408
1	0	0.181332	4.540476	-2.646904
1	0	3.487120	0.042857	-3.531702
1	0	1.741301	0.215639	-3.833347
1	0	2.349781	-0.933770	-2.616697
1	0	7.430374	-4.141975	1.405536
79	0	0.106789	0.976379	-1.054930
15	0	-1.594035	-0.207733	0.032237
6	0	-2.418057	-1.282187	-1.200055
6	0	-1.609529	-2.202371	-1.881054
6	0	-3.755835	-1.198649	-1.600425
6	0	-2.092462	-3.013478	-2.897583
6	0	-4.263042	-2.001135	-2.620380
6	0	-3.432579	-2.910216	-3.268211
6	0	-2.852539	0.799114	0.887727
6	0	-3.827853	0.196229	1.694915
6	0	-2.847666	2.198653	0.830951
6	0	-4.746553	0.936859	2.424255
6	0	-3.759723	2.962332	1.554673
6	0	-4.707949	2.329698	2.352942
6	0	-0.919769	-1.291224	1.351524
6	0	-1.277358	-2.626680	1.575665
6	0	0.052788	-0.745816	2.201537
6	0	-0.676791	-3.387394	2.574881
6	0	0.661415	-1.484350	3.207960
6	0	0.295463	-2.816010	3.391179
9	0	0.413067	0.540815	2.067567
9	0	-2.216817	-3.226305	0.836988
9	0	-1.037668	-4.658499	2.757485
9	0	0.866582	-3.536844	4.351054
9	0	1.582350	-0.926595	3.995900
9	0	-3.900935	-1.139617	1.757879
9	0	-5.663835	0.331731	3.178953
9	0	-5.582837	3.051946	3.043794
9	0	-3.727437	4.293634	1.482694
9	0	-1.963007	2.863119	0.077550
9	0	-0.315583	-2.316332	-1.537455
9	0	-4.603970	-0.332561	-1.033208
9	0	-5.544071	-1.895700	-2.977426
9	0	-3.914784	-3.678077	-4.240612
9	0	-1.289851	-3.881055	-3.515097
1	0	1.066726	2.309784	-3.199339
1	0	6.251315	0.718941	2.300426

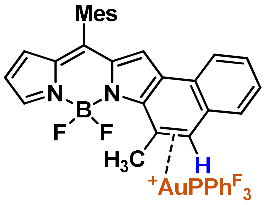

Standard orientation:

Atomic Number	Atomic Type	Coordinates (Angstroms) X	Y	Z
6	0	5.501068	-4.283017	0.346285
6	0	4.359394	-4.133373	-0.486359
7	0	4.098741	-2.841150	-0.696370
6	0	5.945147	-3.011328	0.644259
6	0	5.063085	-2.094318	-0.010426
6	0	5.040766	-0.711783	-0.035345
6	0	4.019542	-0.036036	-0.766923
7	0	3.051561	-0.708834	-1.503510
6	0	2.219602	0.246182	-2.036852
6	0	2.666759	1.543773	-1.630715
6	0	3.803854	1.347585	-0.838296
5	0	2.957070	-2.267796	-1.598376
9	0	1.727379	-2.708457	-1.109490
9	0	3.161970	-2.703685	-2.901610
6	0	1.988341	2.750267	-2.030234
6	0	0.820316	2.609573	-2.828627
6	0	2.427454	4.043355	-1.677845
6	0	1.732760	5.162128	-2.108856
6	0	0.581992	5.023289	-2.909591
6	0	0.131428	3.763065	-3.263398
6	0	0.369926	1.279334	-3.211007
6	0	1.075137	0.091603	-2.894521
6	0	0.706351	-1.196447	-3.589607
1	0	3.740930	-4.904811	-0.926337
1	0	6.791525	-2.727984	1.254045
1	0	4.413838	2.095269	-0.352738
1	0	3.318201	4.159626	-1.067965
1	0	2.081539	6.152801	-1.832667
1	0	0.050071	5.906008	-3.251368
1	0	-0.755373	3.648736	-3.881319
1	0	1.537372	-1.517832	-4.224892
1	0	-0.180292	-1.048886	-4.211221
1	0	0.518547	-2.001650	-2.878604
1	0	5.921219	-5.224784	0.669813

79	0	-0.963210	0.614042	-1.462842
15	0	-2.471518	0.006848	0.221920
6	0	-3.555563	-1.314139	-0.435856
6	0	-2.925822	-2.466064	-0.926457
6	0	-4.944638	-1.241144	-0.585949
6	0	-3.625350	-3.503051	-1.526487
6	0	-5.668209	-2.268900	-1.187265
6	0	-5.009682	-3.401253	-1.656271
6	0	-3.507007	1.346157	0.903123
6	0	-4.312116	1.132181	2.030930
6	0	-3.485740	2.642177	0.371851
6	0	-5.048270	2.150437	2.619128
6	0	-4.216402	3.679512	0.945339
6	0	-4.996021	3.432307	2.071429
6	0	-1.593700	-0.675869	1.682799
6	0	-1.962064	-1.827657	2.389907
6	0	-0.440795	-0.008013	2.119755
6	0	-1.208117	-2.303892	3.458858
6	0	0.322279	-0.461998	3.187559
6	0	-0.061845	-1.621539	3.856908
9	0	-0.048615	1.122797	1.511289
9	0	-3.060342	-2.517551	2.066211
9	0	-1.585786	-3.407055	4.106571
9	0	0.658065	-2.068606	4.880922
9	0	1.412834	0.205688	3.573118
9	0	-4.403266	-0.096038	2.556704
9	0	-5.806611	1.911419	3.689199
9	0	-5.698430	4.416119	2.622005
9	0	-4.173463	4.903498	0.417628
9	0	-2.761899	2.938004	-0.714794
9	0	-1.592629	-2.584001	-0.810185
9	0	-5.638507	-0.173585	-0.173791
9	0	-6.992161	-2.166242	-1.315187
9	0	-5.698387	-4.384032	-2.228550
9	0	-2.986479	-4.584383	-1.974499
1	0	-0.362706	1.222690	-4.015076
6	0	6.068343	0.079558	0.707543
6	0	7.241041	0.495169	0.040566
6	0	5.855047	0.404484	2.064313
6	0	8.189293	1.233613	0.755744
6	0	6.833605	1.147207	2.733622
6	0	8.008474	1.567571	2.101982
1	0	9.093235	1.556278	0.243910
1	0	6.669580	1.403991	3.777830
6	0	4.601004	-0.022065	2.794636
1	0	3.694377	0.348369	2.303506
1	0	4.513885	-1.113946	2.845717
1	0	4.602543	0.359361	3.819452
6	0	7.486831	0.156896	-1.413363
1	0	7.506034	-0.926816	-1.578924
1	0	6.703845	0.565949	-2.062859
1	0	8.445106	0.563426	-1.748115
6	0	9.063666	2.340293	2.858249
1	0	9.798694	1.661694	3.311017
1	0	9.612911	3.019058	2.197609
1	0	8.623733	2.930264	3.668866

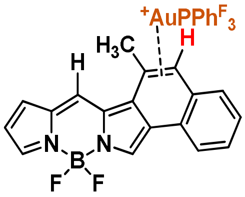

Standard orientation:

Atomic Number	Atomic Type	Coordinates (Angstroms) X	Y	Z
6	0	7.247342	-3.427288	0.057951
6	0	7.352455	-2.257523	0.843940
7	0	6.401208	-1.374578	0.498496
6	0	6.176868	-3.235691	-0.802884
6	0	5.646047	-1.945133	-0.528048
6	0	4.598691	-1.250482	-1.119733
6	0	4.255470	0.047139	-0.730025
7	0	4.968973	0.667923	0.291233
6	0	4.500612	1.907511	0.479643
6	0	3.439328	2.164228	-0.416659
6	0	3.271924	0.981905	-1.190974
5	0	6.128617	0.014683	1.145719
9	0	7.252131	0.820005	1.078898
9	0	5.694307	-0.133283	2.453981
6	0	2.646005	3.343626	-0.616041
6	0	1.646811	3.284870	-1.632662
6	0	2.817980	4.540836	0.113249
6	0	2.032653	5.646822	-0.161855
6	0	1.054330	5.594282	-1.176408
6	0	0.862800	4.429976	-1.899311
6	0	1.460244	2.054717	-2.392153
6	0	2.288552	0.919662	-2.227645
6	0	2.212812	-0.230683	-3.200926
1	0	8.063837	-2.029363	1.626729
1	0	5.799611	-3.915572	-1.555048
1	0	4.934346	2.552149	1.231432
1	0	3.574579	4.593057	0.890559
1	0	2.174884	6.563319	0.403247
1	0	0.451141	6.471228	-1.391230
1	0	0.108367	4.384972	-2.680701
1	0	3.168418	-0.333829	-3.730443
1	0	1.427768	-0.061499	-3.942557
1	0	2.004931	-1.178768	-2.695645
1	0	7.891943	-4.292031	0.129106
79	0	-0.083254	0.928945	-1.131282
15	0	-1.757147	-0.236061	0.014624
6	0	-2.098499	-1.772470	-0.921538
6	0	-1.000949	-2.596414	-1.206748
6	0	-3.331503	-2.154887	-1.462736
6	0	-1.105619	-3.742752	-1.980615
6	0	-3.460143	-3.300199	-2.246206
6	0	-2.348239	-4.095958	-2.504311
6	0	-3.317398	0.669719	0.278409
6	0	-4.321812	0.153347	1.109533
6	0	-3.537364	1.937642	-0.275728
6	0	-5.481140	0.859608	1.395901
6	0	-4.692464	2.664879	-0.001923
6	0	-5.663090	2.125170	0.837412
6	0	-1.213575	-0.721489	1.696821
6	0	-1.394290	-1.982870	2.278472
6	0	-0.544850	0.241278	2.466470
6	0	-0.912560	-2.277238	3.550914
6	0	-0.060626	-0.028517	3.740036
6	0	-0.244260	-1.298872	4.282537
9	0	-0.369492	1.480948	1.981120
9	0	-2.043355	-2.956878	1.631771
9	0	-1.098657	-3.490142	4.073825
9	0	0.209546	-1.573230	5.501453
9	0	0.568147	0.916088	4.440844
9	0	-4.184572	-1.071994	1.631993
9	0	-6.419266	0.336904	2.186058
9	0	-6.768969	2.811578	1.101615
9	0	-4.870541	3.871745	-0.540903
9	0	-2.641682	2.504739	-1.094215
9	0	0.209119	-2.275125	-0.713173
9	0	-4.439706	-1.430352	-1.264680

9	0	-4.649736	-3.633246	-2.750654
9	0	-2.469925	-5.190390	-3.249002
9	0	-0.034875	-4.499103	-2.222598
1	0	0.872632	2.123486	-3.307872
1	0	4.049946	-1.736625	-1.916328

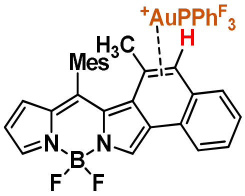

Standard orientation:

Atomic Number	Atomic Type	Coordinates (Angstroms) X	Y	Z
6	0	-7.805660	0.685838	1.994347
6	0	-7.440393	-0.655929	2.221858
7	0	-6.315272	-0.949150	1.547940
6	0	-6.845755	1.224158	1.150418
6	0	-5.906185	0.192547	0.861394
6	0	-4.781978	0.220962	0.015558
6	0	-4.017684	-0.953297	-0.207759
7	0	-4.402130	-2.103287	0.489452
6	0	-3.640534	-3.139929	0.146683
6	0	-2.683566	-2.739463	-0.802168
6	0	-2.893150	-1.345658	-1.049038
5	0	-5.465285	-2.226309	1.648274
9	0	-6.225998	-3.366414	1.451814
9	0	-4.788252	-2.279200	2.861928
6	0	-1.692386	-3.548918	-1.449797
6	0	-0.841738	-2.899311	-2.383159
6	0	-1.536881	-4.934932	-1.225207
6	0	-0.573177	-5.649000	-1.914770
6	0	0.262996	-5.004856	-2.851584
6	0	0.132006	-3.647937	-3.081629
6	0	-1.020328	-1.477250	-2.614850
6	0	-2.054679	-0.702493	-2.030806
6	0	-2.281965	0.665298	-2.619181
1	0	-7.927524	-1.402769	2.835524
1	0	-6.805948	2.230705	0.757111
1	0	-3.804858	-4.111851	0.588886
1	0	-2.179445	-5.444154	-0.513422
1	0	-0.462858	-6.714034	-1.738463
1	0	1.008797	-5.578510	-3.393737
1	0	0.775447	-3.144243	-3.798203
1	0	-3.256894	0.693824	-3.113850
1	0	-1.517327	0.879759	-3.369958
1	0	-2.272563	1.459926	-1.874854
1	0	-8.675629	1.181535	2.404686
79	0	0.463185	-0.582797	-1.129621
15	0	2.265443	0.230724	0.127941
6	0	2.191999	2.061035	0.086750
6	0	1.091960	2.666974	0.705544
6	0	3.053238	2.898026	-0.629365
6	0	0.855074	4.032075	0.642697
6	0	2.838578	4.273234	-0.703702
6	0	1.738011	4.841523	-0.069308
6	0	3.913504	-0.306285	-0.451374
6	0	5.075337	0.126468	0.203589
6	0	4.078306	-1.215509	-1.504651
6	0	6.340279	-0.310790	-0.161742
6	0	5.337868	-1.671441	-1.886636
6	0	6.469058	-1.217832	-1.214226
6	0	2.240495	-0.290500	1.889005
6	0	2.542501	0.523003	2.988973
6	0	1.899872	-1.623316	2.164969
6	0	2.490958	0.041120	4.293407
6	0	1.845167	-2.126180	3.458025
6	0	2.141313	-1.286858	4.529272
9	0	1.632167	-2.468785	1.156889
9	0	2.892699	1.803937	2.829865
9	0	2.781146	0.845033	5.317025
9	0	2.098462	-1.752678	5.772777
9	0	1.520301	-3.401875	3.675395
9	0	4.977795	1.008020	1.207366
9	0	7.424052	0.129663	0.476983
9	0	7.672923	-1.646183	-1.575120
9	0	5.460630	-2.539698	-2.890713
9	0	3.030554	-1.693047	-2.188076
9	0	0.227029	1.900700	1.389884
9	0	4.109713	2.410559	-1.290993
9	0	3.681410	5.043596	-1.393384
9	0	1.528100	6.152539	-0.143801
9	0	-0.211917	4.566086	1.246521
1	0	-0.554953	-1.073166	-3.512075
6	0	-4.486055	1.557951	-0.587182
6	0	-3.596608	2.425335	0.081611
6	0	-5.158630	1.975549	-1.755312
6	0	-3.346756	3.686923	-0.472117
6	0	-4.882521	3.248665	-2.264969
6	0	-3.976857	4.117362	-1.644831
1	0	-2.640759	4.345943	0.029156
1	0	-5.387201	3.567317	-3.174631
6	0	-6.150733	1.079085	-2.464618
1	0	-5.719882	0.100921	-2.708435
1	0	-7.032923	0.888865	-1.842828
1	0	-6.491278	1.538211	-3.396668
6	0	-2.932299	2.027560	1.380875
1	0	-2.214417	2.786222	1.695605
1	0	-3.671999	1.909587	2.179996
1	0	-2.398219	1.075556	1.296719
6	0	-3.714404	5.494019	-2.209484
1	0	-3.741422	5.488940	-3.304192
1	0	-4.475423	6.209194	-1.870465
1	0	-2.740620	5.878474	-1.890351

7. References

[1] N. R. Babij, E. O. McCusker, G. T. Whiteker, B. Canturk, N. Choy, L. C. Creemer, C. V. D. Amicis, N. M. Hewlett, P. L. Johnson, J. A. Knobelsdorf, F. Li, B. A. Lorsbach, B. M. Nugent, S. J. Ryan, M. R. Smith and Q. Yang, *Org. Process Res. Dev.*, 2016, **20**, 661.

[2] J. R. Lakowicz, *Principles of fluorescence spectroscopy*, 3rd ed. Springer, New York, 2006.

[3] A. M. Brouwer, *Pure Appl. Chem.*, 2011, **83**, 2213.
[4] The authors thank X-Ray laboratory for the crystallographic support.
[5] (a) C. Korner, P. Starkov and T. D. Sheppard, *J. Am. Chem. Soc.,* 2010, **132**, 5968. (b) Y. Hayashi, S. Yamaguchi, W. Y. Cha, D. Kim, H. Shinokubo, *Org. Lett*., 2011, **13**, 2992. (c) T. Sakida, S. Yamaguchi, H. Shinokubo, *Angew. Chem. Int. Ed.*, 2011, **50**, 2280.
[6] (a) C. Lee, W. Yang, R. G. Parr, *Phys. Rev. B* 1988, **37**, 785–789. (b) A. D. Becke, *J. Chem. Phys.* 1993, **98**, 5648. (c) W. Kohn, A. D. Becke, R. G. Parr, *J. Phys. Chem.* 1996, **100**, 12974–12980.
[7] (a) P. J. Hay, W. R. Wadt, *J. Chem. Phys.,* 1985, **82**, 270-283. (b) P. J. Hay, W. R. Wadt, *J. Chem. Phys.* 1985, **82**, 284. (c) P. J. Hay, W. R. Wadt, *J. Chem. Phys.*, 1985, **82**, 299-310.
[8] Y. Zhao, D. G. Truhlar, *Theor. Chem. Acc.*, 2008, **120**, 215.
[9] Gaussian 16, Revision C.01; Frisch, M. J.; Trucks, G. W.; Schlegel, H. B.; Scuseria, G. E.; Robb, M. A.; Cheeseman, J. R.; Scalmani, G.; Barone, V.; Petersson, G. A.; Nakatsuji, H.; Li, X.; Caricato, M.; Marenich, A. V.; Bloino, J.; Janesko, B. G.; Gomperts, R.; Mennucci, B.; Hratchian, H. P.; Ortiz, J. V.; Izmaylov, A. F.; Sonnenberg, J. L.; Williams-Young, D.; Ding, F.; Lipparini, F.; Egidi, F.; Goings, J.; Peng, B.; Petrone, A.; Henderson, T.; Ranasinghe, D.; Zakrzewski, V. G.; Gao, J.; Rega, N.; Zheng, G.; Liang, W.; Hada, M.; Ehara, M.; Toyota, K.; Fukuda, R.; Hasegawa, J.; Ishida, M.; Nakajima, T.; Honda, Y.; Kitao, O.; Nakai, H.; Vreven, T.; Throssell, K.; Montgomery, J. A., Jr.; Peralta, J. E.; Ogliaro, F.; Bearpark, M. J.; Heyd, J. J.; Brothers, E. N.; Kudin, K. N.; Staroverov, V. N.; Keith, T. A.; Kobayashi, R.; Normand, J.; Raghavachari, K.; Rendell, A. P.; Burant, J. C.; Iyengar, S. S.; Tomasi, J.; Cossi, M.; Millam, J. M.; Klene, M.; Adamo, C.; Cammi, R.; Ochterski, J. W.; Martin, R. L.; Morokuma, K.; Farkas, O.; Foresman, J. B.; Fox, D. J. Gaussian, Inc., Wallingford CT, 2016.
